# High-Throughput Metabolomics for Discovering Potential Biomarkers and Identifying Metabolic Mechanisms in Aging and Alzheimer’s Disease

**DOI:** 10.3389/fcell.2021.602887

**Published:** 2021-02-25

**Authors:** Kun Xie, Qi Qin, Zhiping Long, Yihui Yang, Chenghai Peng, Chunyang Xi, Liangliang Li, Zhen Wu, Volontovich Daria, Yashuang Zhao, Fan Wang, Maoqing Wang

**Affiliations:** ^1^Department of Epidemiology, School of Public Health, Harbin Medical University, Harbin, China; ^2^Department of Neurology, Innovation Center for Neurological Disorders, National Clinical Research Center for Geriatric Diseases, Xuan Wu Hospital, Capital Medical University, Beijing, China; ^3^The Forth Affiliated Hospital of Harbin Medical University, Harbin, China; ^4^Department of Orthopedic Surgery, The Second Affiliated Hospital of Harbin Medical University, Harbin, China; ^5^National Key Disciplines of Nutrition and Food Hygiene, Department of Nutrition and Food Hygiene, School of Public Health, Harbin Medical University, Harbin, China

**Keywords:** aging, Alzheimer’s disease, mild cognitive impairment, metabolic biomarkers, metabolomics

## Abstract

Alzheimer’s disease (AD) is an aging-related neurodegenerative disease. We aimed to investigate the metabolic mechanisms of aging and AD and to identify potential biomarkers for the early screening of AD in a natural aging population. To analyze the plasma metabolites related to aging, we conducted an untargeted metabolomics analysis using ultra-high-performance liquid chromatography/quadrupole time-of-flight mass spectrometry in a two-stage cross-sectional study. Spearman’s correlation analysis and random forest were applied to model the relationship between age and each metabolite. Moreover, a systematic review of metabolomics studies of AD in the PubMed, Cochrane and Embase databases were searched to extract the differential metabolites and altered pathways from original studies. Pathway enrichment analysis was conducted using Mummichog. In total, 669 metabolites were significantly altered with aging, and 12 pathways were enriched and correlated with aging. Three pathways (purine metabolism, arginine and proline metabolism, and the TCA cycle) were shared between aging and AD. Arginine and proline metabolism play a key role in the progression from healthy to mild cognitive impairment and to AD in the natural aging population. Three metabolites, 16-a-hydroxypregnenolone, stearic acid and PC[16:0/22:5(4Z,7Z,10Z,13Z,16Z)] were finally proposed as potential markers of AD in the natural aging population. The underlying mechanism shared between aging and AD and the potential biomarkers for AD diagnosis were proposed based on multistep comparative analysis.

## Introduction

Alzheimer’s disease (AD) is an aging-related neurodegenerative disease characterized by senile plaques caused by amyloid beta (Aβ) and neurofibrillary tangles containing hyperphosphorylated tau-protein. Current estimates suggest that 44 million people live with dementia worldwide. This figure is predicted to more than triple by 2050 as the population ages; at this time, the annual cost of dementia in the United States alone may exceed US$600 billion (Lane et al., 2017; Nichols et al., 2019). Mild cognitive impairment (MCI, “amnestic MCI” is seen as a prodromal stage of Alzheimer’s disease) represents the clinically diagnosed pre-dementia stage. Several studies have shown that the pathological changes of AD begin several years before the onset of evident memory impairment (Jack et al., 2010; Fleisher et al., 2012). However, the current diagnosis is based on clinical symptoms of AD combined with pathological alterations, such as a decrease of cerebrospinal fluid Aβ42 or an increase in p-tau or t-tau protein, which may not identify a substantial number of asymptomatic individuals who will develop AD later. Thus, it is urgent to investigate the physiology of AD and/or MCI and detect early biomarkers to improve the quality of life of those affected by this disease.

Although genetic factors involved in the development of AD have been identified, studies have also suggested that aging is the major risk factor. After the age of 60 years, the incidence of AD was found to double with every 5 years increase in age (Mayeux and Stern, 2012). Although the mechanisms triggering alterations associated with both aging and AD are not completely understood, they have been simultaneously divided into at least two aspects: oxidative stress and inflammation. First, increasing oxidative stress is observed in the contexts of aging, AD and/or MCI in terms of membrane lipids (Mielke and Lyketsos, 2006; Zhu et al., 2006; Markesbery and Lovell, 2007), proteins (Poon et al., 2006; Greilberger et al., 2008), and mitochondrial DNA (Bender et al., 2006; Wang et al., 2006). Second, inflammation is found to be a considerable driving force of aging and AD. In addition, recent studies have identified common histological changes (Ohm et al., 1995; Naslund et al., 2000) and modulation of neurotransmission (Kern and Behl, 2009), including hypofunction of the cholinergic system, in AD and aging (Bartus, 2000; Gron et al., 2006). Thus, the relationship between aging and AD can be revealed.

Metabolomics has been widely used to provide an overall description of metabolic profiles in pathological or physiological processes (Psychogios et al., 2011). Metabolomics offers quantitative measurement of final products downstream of interactions among genes, proteins and various influences. Compared with genomics and proteomics, metabolomics is regarded as an optimal platform to describe a dynamic physiological process and integral disease response. Evidence has shown that changes in metabolites are significant early indicators of diseases (Nicholson et al., 2008). A growing body of literature has already reported distinct perturbed sets of metabolites in the contexts of aging and AD (Yu et al., 2012; Hertel et al., 2016; Rist et al., 2017; Wilkins and Trushina, 2017). Thus, metabolomics is also a promising tool to systematically assess changes of small molecules in both natural aging and AD populations and provides clues essential for the early diagnosis of AD. However, little effort to date has been made to detect and compare the metabolites and metabolic pathways between AD and natural aging.

Therefore, we carried out a metabolomics analysis of aging-related metabolites and a systematic review of differential metabolites related to MCI and AD. Based on these findings, we aimed to explore similar metabolomic signatures among AD, MCI and aging, which could help explain the high incidence of AD in older populations and suggest novel markers to identify the earliest phase of AD in the natural aging population.

## Materials and Methods

### Global Plasma Metabolic Profiling Analysis of Aging in a Two-Stage Cross-Sectional Study

#### Study Sample Collection and Detection

Subjects were enrolled from community-dwelling individuals from the Xiangfang community and surrounding villages in the city of Harbin and orthopedic and ophthalmic patients attending the Second Affiliated Hospital of Harbin Medical University. The training set consisted of 119 participants enrolled in the first half year of 2010, while the testing set of 64 individuals was enrolled in the next half year. All subjects completed a comprehensive questionnaire to obtain information about sociodemographic characteristics, lifestyle, and history of some diseases, such as diabetes, hypertension and heart disease. Fasting peripheral venous blood (5 ml) was collected using an EDTA tube and treated with centrifugation for 10 min at 3,000 rpm and 4°C. Then, samples were frozen at −80°C prior to measurement.

We carried out this study after obtaining written informed consent from all the study subjects and approval from the Human Research and Ethics Committee of Harbin Medical University. All experiments, including relevant details, were performed in accordance with relevant guidelines and regulations.

A detailed description of the experimental protocol of metabolic profiling analysis for plasma by UPLC/Q-TOF-MS/MS and the method of metabolites identification were provided in Supplementary Materials 1.1.

#### Statistical Analysis

The metabolites detected in both the training and testing sets were considered the stably detected metabolites. Correlations between ion intensities of metabolites and age were calculated by Spearman’s correlation analysis. The analytes with *P-*values less than 0.05 were selected for the following statistical analyses. The set of aging-related metabolites was chosen according to the importance score given by random forest, in which age served as the dependent variable and all Spearman-correlated features served as independent variables. A total of 30 variables were assigned variable importance scores and identified with the HMDB database. All the Spearman-correlated metabolites were analyzed for pathway enrichment analysis using Mummichog^1^. All calculations were performed with the R statistical platform, version 3.4.4.

### Systematic Review of Metabolomics Studies of AD and MCI

#### Literature Search Strategy

We conducted an English language literature search for metabolomic studies of MCI and AD. The search was conducted in PubMed, Cochrane and Embase through August 27, 2018 with the search terms “Alzheimer disease,” “Alzheimer’s disease,” and “mild cognitive impairment” combined with “metabolomics,” “metabonomics,” “GC-MS,” and “LC-MS.” Both automatic retrieval and manual retrieval were used for the literature search. In addition, we augmented the search by a snowball strategy, screening the references of original texts and reviews.

A detailed description for the criteria of literation inclusion and exclusion and data extraction was provided in Supplementary Materials 1.2.

#### Quality Assessment of Individual Studies

The QUADAS (Lumbreras et al., 2008) (Quality Assessment of Diagnostic Accuracy Studies) is a well-established tool used for the appraisal of quality issues of omics-based studies investigating new diagnostic tests. QUADOMICS included sixteen questions, where the risk of bias could be appraised with respect to four aspects: “patient selection,” “index test,” “reference standard,” and “flow and timing” (Whiting et al., 2011). Possible answers for each item were Y (criteria achieved), N (criteria not achieved), ? (unclear), and NA (not applicable).

#### Pathway Enrichment Analysis

We directly extracted the enriched pathways from original studies. Moreover, we conducted pathway enrichment analyses based on all the metabolites extracted from original studies using online analysis platform MetaboAnalyst^2^. All these pathways were combined and categorized according to different biosamples (blood, urine, tissue, or CFS) or comparisons.

## Results

### Global Plasma Metabolic Profiling Analysis of Aging in a Two-Stage Cross-Sectional Study

#### Demographical Characteristics of the Study Population

The training set involved 119 participants, aged 32–82 years, with an average age of 58.66 years and BMI of 24.10 kg m^–2^. The testing set contained 64 people, aged from 25 to 85 years, with a mean age of 58.98 years and BMI of 23.93 kg m^–2^. Other information on education, smoking, alcohol consumption, chronic diseases and exercises is shown in Supplementary Table 1. No significant difference of any characteristics was observed.

#### Quality Assessment of the Metabolomics Platform

Quality control (QC) samples were all clustered tightly in the two-stage cross-sectional detection. The relative standard deviations (RSDs;%) of the retention time and peak area ranged from 0 to 0.73 and 0.8 to 4, respectively, in the intra-batch assay and ranged from 0.1 to 2.7 and 1.5 to 6.2, respectively, in the inter-batch assay. The results showed that the stability of the UPC/Q-TOF MSMS platform was excellent throughout the run and was sufficient to ensure data quality for further global metabolic profiling analyses.

#### Identification of Aging-Related Metabolites and Pathway Enrichment Analysis

By including all duplicated metabolites in Spearman correlation analysis, we found that the ion intensities of 381 metabolites in ESI^–^ and 288 metabolites in ESI^+^ were significantly associated with age (*P* < 0.05, Excel 1&2^3^). Then, we employed an additional statistical method, random forest, to screen out the top 30 metabolites among all 669 metabolites, and 26 of them were identified successfully (Table 1 and Supplementary Figure 1). Several classes of metabolites were observed twice or more often, such as dipeptides, long-chain fatty acids, triterpenoids, steroid glucuronide conjugates, fatty acid esters and phosphatidylcholines.

**TABLE 1:** Top 30 aging-related metabolites in plasma screened by random forest.

Categories/Number	RT^a^	Mass	HMDB ID^b^	Metabolites’ name	Additive ion	ESI mode
**Dipeptides**						
1	2.81	283.0861	HMDB0028853	Glycyl-Tyrosine	M + FA-H	ESI-
2	2.66	248.1024	HMDB0029060	Threoninyl-Glutamate	M + H	ESI+
3	2.67	265.1484	HMDB0029008	Phenylalanyl-Valine	M + H	ESI+
**Long-chain fatty acids**						
4	3.71	307.1821	HMDB0000672	Hexadecanedioic acid	M + Na-2H	ESI-
5	3.81	257.1785	HMDB0000872	Tetradecanedioic acid	M-H	ESI-
**6**	**3.50**	**321.2046**	**HMDB0000827**	**Stearic acid**	**M + K-2H**	**ESI-**
**Triterpenoids**						
7	4.49	449.2562	HMDB0002385	Celastrol	M-H	ESI-
8	3.94	551.3198	HMDB0004309	Triterpenoid	M-H	ESI-
**Steroid glucuronide conjugates**						
9	3.61	597.3527	HMDB0002513	Lithocholate 3-O-glucuronide	M + FA-H	ESI-
10	3.4	565.3029	HMDB0002577	Cholic acid glucuronide	M-H20-H	ESI-
**Fatty acid esters**						
11	3.69	183.1395	HMDB0031272	Ethyl (E)-2-non-enoate	M-H	ESI-
12	3.57	465.2487	HMDB0029886	Sorbitan oleate	M + K-2H	ESI-
**Phosphatidylcholine**						
13	9.16	758.5624	HMDB0007880	PC[14:0/20:2(11Z,14Z)]	M + H	ESI+
**14**	**9.42**	**846.5454**	**HMDB0007989**	**PC[16:0/22:5(4Z,7Z,10Z,13Z,16Z)]**	**M + K**	**ESI +**
**1,2-diacylglycerol-3-phosphates**						
15	9.41	701.5504	HMDB0114824	1,2-diacylglycerol-3-phosphates	ESI+	
**1-acylglycerol-3-phosphates**						
16	4.19	485.2756	HMDB0114752	LysoPA[22:4(7Z,10Z,13Z,16Z)/0:0]	M-H	ESI-
**Gluco/mineralocorticoids, progestogins and derivatives**						
**17**	**3.71**	**369.1899**	**HMDB0000315**	**16-a-Hydroxypregnenolone**	**M + K-2H**	**ESI-**
**Phenylpropanoic acids**						
18	2.59	145.0582	HMDB0001955	3-Phenylbutyric acid	M-H20-H	ESI-
**Hydroxyindoles**						
19	2.59	263.1037	HMDB0001238	N-Acetylserotonin	M + FA-H	ESI-
**Hypoxanthines**						
20	2.67	130.0577	HMDB0000897	7-Methylguanine	M-2H2O + H	ESI +
**Acylcarnitines**						
21	3.12	304.2231	HMDB0061634	3-hydroxyoctanoyl carnitine	M + H	ESI +
**Phosphatidylglycerophosphates**						
22	4.00	735.3353	HMDB0033168	(15a,20R)-Dihydroxypregn-4-en-3-one	M + K-2H	ESI-
				20-[glucosyl-(1- > 4)-6-acetyl-glucoside]		
**Medium-chain fatty acids**						
23	3.55	167.1402	HMDB0000947	Undecanoic acid	M-H20-H	ESI-
**Oligopeptides**						
24	3.83	444.273	HMDB0012936	Dynorphin B (10–13)	M-H	ESI-
**Purine 2′-deoxyribonucleosides**						
25	2.66	275.065	HMDB0000071	Deoxyinosine	M + Na	ESI+
**Prostaglandins and related compounds**						
26	3.73	315.189	HMDB0060046	15d PGD2	M-H20-H	ESI-

*^a^Rention time. ^b^The HMDB identifier of the metabolite. Bold, 3 metabolites related to both aging and AD, including 16-a-Hydroxypregnenolone, stearic acid, and PC[16:0/22:5(4Z,7Z,10Z,13Z,16Z)].*

Table 2 summarizes the pathways enriched by all metabolites correlated with aging using Mummichog. A total of 12 pathways were significantly perturbed with a *P* < 0.05.

**TABLE 2:** Aging-related pathways enriched by spearman-correlated metabolites.

Pathways’ name	Total no.	Hits no.	*P*-value
Carnitine shuttle^a^	15	4	0.021
Omega-3 fatty acid metabolism^a^	5	2	0.035
Ascorbate (Vitamin C) and Aldarate Metabolism^b^	2	2	0.007
Glutamate metabolism^b^	6	3	0.010
Biopterin metabolism^b^	10	4	0.012
TCA cycle^b^	3	2	0.019
Vitamin B3 (nicotinate and nicotinamide) metabolism^b^	15	5	0.021
Purine metabolism^b^	23	7	0.023
Tyrosine metabolism^b^	31	9	0.026
Aspartate and asparagine metabolism^b^	31	9	0.026
Carnitine shuttle^b^	12	4	0.031
Leukotriene metabolism^b^	21	6	0.044
Arginine and Proline Metabolism^b^	17	5	0.045

*^a^Pathway enrichment conducted using negative-mode features. ^b^Pathway enrichment conducted using positive-mode features.*

### Systematic Review of Metabolomics Studies of AD and MCI

#### Literature Retrieval

In total, 494 articles from PubMed, 23 articles from Cochrane, and 64 articles from Embase were retrieved from automatic electronic searches. A total of 581 publications were examined through title and abstract screening. There were 113 records left after excluding the literatures that were duplicates, reviews, or not related to the research topic (e.g., mechanisms or drug use exploration and technology assessment). Additionally, 9 articles were manually retrieved after searching references from original articles. After careful full-text screening, 67 studies were finally included in this systematic review (Supplementary Figure 2).

#### Description of Included Studies

Supplementary Table 2 summarizes the characteristics of the 67 included studies regarding the number of subjects, sample source, sample type, platforms and outcomes. Of the 67 studies, the number of AD cases ranged from 7 to 1,356, the number of MCI cases ranged from 10 to 356, and the number of healthy controls ranged from 7 to 23,882. The detection platform includes LC-MS in 28 studies, GC-MS in 13 studies, multiple platforms in 11 studies, nuclear magnetic resonance (NMR) in 4 studies, and other platforms in 8 studies. Various biosamples used included serum (18 studies), plasma (16 studies), brain tissue (10 studies), CSF (8 studies), CSF and plasma (5 studies), brain tissue and plasma (1 study), urine (4 study), and others (5 studies). Additionally, 83.58% of the studies were case-control studies, and 41.79% of the subjects in these studies were European (Supplementary Figure 3).

#### Quality Assessment of Eligible Studies

According to the QUADOMICS tool, 30 out of 67 articles were unable to avoid overfitting due to the lack of a statistical approach such as cross-validation or an independent test set. All of the articles explored differences in biomarkers between overt cases and healthy individuals and could be categorized into preliminary phase 1 studies. Items with respect to the representative feature of the included subjects and the availability of the clinical data were not applicable for all articles. Supplementary Table 3 presents a detailed assessment for all 67 studies.

#### Metabolites Related to the Occurrence and Progression of AD and MCI

Supplementary Tables 4–8 presents the metabolites that were extracted as biological markers in original metabolomics studies. There were 830 altered analytes in the comparison of AD VS. CN, with 137 analytes reported twice or more often (Supplementary Table 4). Tryptophan was the most commonly detected metabolite, followed by palmitic acid, arginine, and L-phenylalanine. The abundance of 293 metabolites was significantly altered in the comparison of MCI VS. CN (Supplementary Table 5); tryptophan was detected 3 times, and 7 other metabolites, such as 5-hydroxytryptophan, L-phenylalanine and L-arginine, were identified twice. For AD VS. MCI, a total of 120 metabolites were affected, including tryptophan (reported 2 times) and histidine (Nichols et al., 2019; Supplementary Table 6). In the prospective studies, 9 and 26 metabolites were found to be related to the progression from CN to AD (CN_AD) and MCI to AD (MCI_AD), respectively (Supplementary Tables 7, 8).

Table 3 illustrates the duplicate metabolites between AD VS. CN and CN_AD VS. CN. Six metabolites (palmitic acid, stearic acid, linoleic acid, glutamine, oleic acid, and myristic acid) were detected in both retrospective case-control and prospective nested case-control studies, and all of these metabolites were detected in serum. In particular, 5 (all except myristic acid) of these 6 metabolites have been found in brain tissue. Additionally, three metabolites (arginine, creatine, and histidine) were detected in both AD VS. MCI and MCI_AD VS. MCI (Table 3), and two (arginine and histidine) of them were confirmed in CSF.

**TABLE 3:** Metabolic biomarkers of Alzheimer’s disease and mild cognitive impairment replicated in both case-control and nested case-control studies.

Metabolites’ Name	HMDB ID	Biosample’s type	
		AD VS. CN (reported frequency^a^)	CN_AD VS. CN (reported frequency)
Palmitic acid	HMDB0000220	Brain tissue, serum (6)	Serum (1)
Stearic acid	HMDB0000827	Brain tissue, serum (2)	Serum (1)
Linoleic acid	HMDB0000673	Brain tissue, serum (2)	Serum (1)
Glutamine	HMDB0000641	Brain tissue, serum, urine (3)	Plasma (1)
Oleic acid	HMDB0000207	Brain tissue, serum (4)	Serum (1)
Myristic acid	HMDB0000806	Serum (2)	Serum (1)

		**Biosample’s type**	
		**AD VS. MCI (reported frequency)**	**MCI_AD VS. MCI (reported frequency)**

Arginine	HMDB0000517	Plasma, CSF (1)	CSF (1)
Creatine	HMDB0000064	Serum (2)	Plasma, CSF (2)
Histidine	HMDB0000177	Serum (2)	CSF (1)

*AD VS. CN, AD VS. MCI, the comparison in case-control studies. CN_AD VS. CN, MCI_AD VS. MCI, the comparison in nested case-control studies. AD, Alzheimer’s disease; CN, healthy controls; MCI, mild cognitive impairment. ^a^Reported frequency of the metabolite in previous studies included in this systematic review.*

#### Metabolic Pathways Associated With AD and MCI

As shown in Supplementary Table 9, there are 53 pathways that exhibited significant alterations in the comparison of AD VS. CN. These pathways were mainly related to amino acid metabolism (including alanine, aspartate and glutamate metabolism and arginine and proline metabolism), tryptophan metabolism, the TCA cycle, and purine metabolism. Additional analysis for the comparison of MCI VS. CN showed 39 metabolic pathways associated with MCI, including lysine metabolism, tryptophan metabolism, polyamine metabolism, and the urea cycle. Comparison of AD VS. MCI presented 38 altered pathways.

**FIGURE 1:**
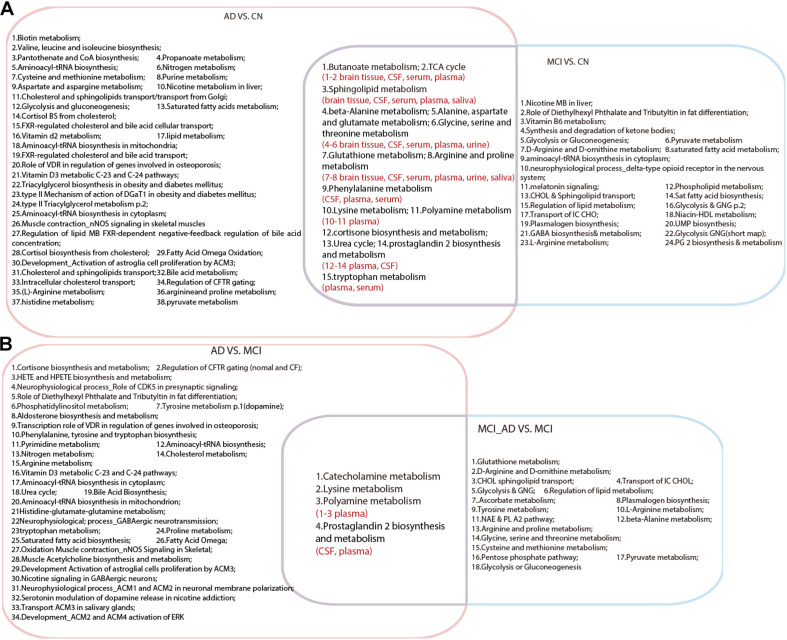
Venn diagram illustrating shared and unique pathways in Alzheimer’s disease and mild cognitive impairment in retrospective and prospective studies. **(A)** Common pathways between AD VS. CN and MCI VS. CN. **(B)** Common pathways between AD VS. MCI and MCI VS. MCI_AD (conversion from MCI to AD). AD, Alzheimer’s disease; CN, healthy controls; MCI, mild cognitive impairment.

As shown in Figure 1, a total of 15 pathways were shared in the comparison between AD VS. CN and MCI VS. CN, which indicated that the metabolic mechanisms of AD and MCI share similar pathological alterations. Moreover, four pathways were shared between AD VS. MCI and MCI_AD VS. MCI, including lysine metabolism, polyamine metabolism, catecholamine metabolism, and prostaglandin 2 biosynthesis and metabolism. These pathway alterations indicated pathological progression from MCI to AD.

#### Pathway Intersection Analysis Among Different Types of Biosamples in AD

**FIGURE 2:**
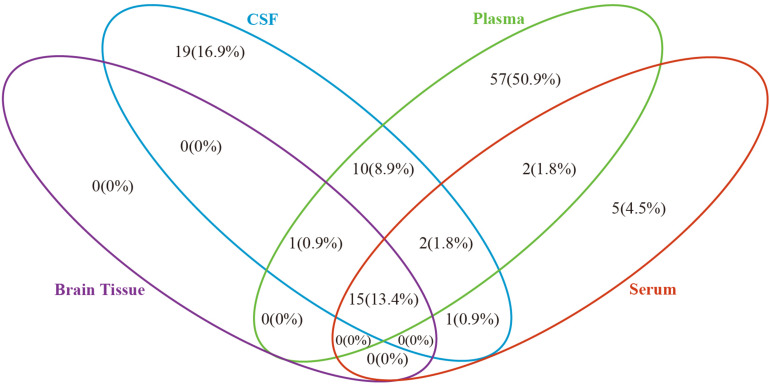
Venn diagram depicting common and unique pathways altered in Alzheimer’s disease in different biosample types. Blue oval represents cerebrospinal fluid, green oval represents plasma, purple oval represents brain tissue and red oval represents serum.

Based on the metabolites extracted from original studies, we performed pathway enrichment analyses by categorizing different types of samples (Supplementary Table 10). Supplementary Table 10 presents the 16, 48, 87, and 25 pathways of AD and MCI enriched in brain tissue, CSF, plasma, and serum, respectively. Figure 2 showed the comparison result of AD, there were 15 common pathways perturbed in all four types of samples, such as sphingolipid metabolism, butanoate metabolism, propanoate metabolism, pantothenate and CoA biosynthesis, aminoacyl-tRNA biosynthesis and some amino acid metabolism pathways. All 16 pathways in brain tissue and most pathways (28/48) in CSF could be detected in plasma.

#### Intersection Analysis of Metabolic Pathways Among AD, MCI, and Aging

**FIGURE 3:**
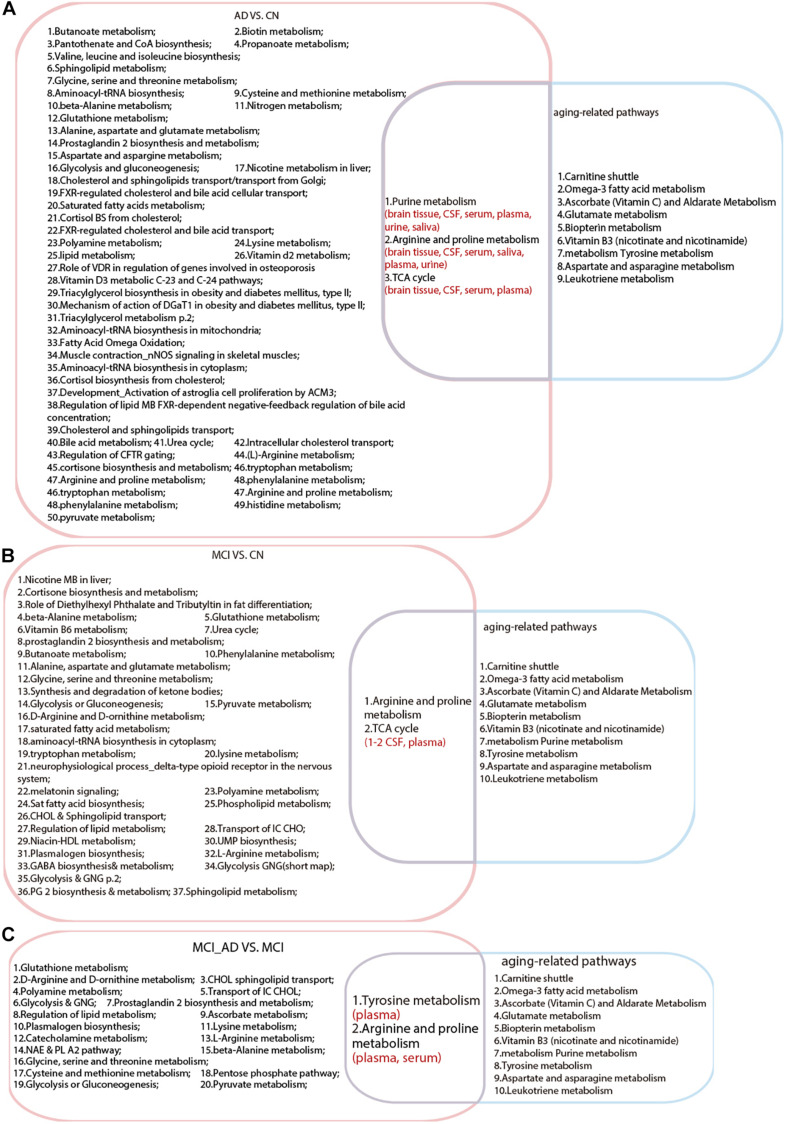
Intersection analysis among pathways in Alzheimer’s disease, mild cognitive impairment and aging. **(A)** Common pathways between AD VS. CN and aging. **(B)** Common pathways between MCI VS. CN and aging. **(C)** Common pathways between MCI VS. MCI_AD (conversion from MCI to AD) and aging. AD, Alzheimer’s disease; CN, healthy controls; MCI, mild cognitive impairment.

We compared the aging-related pathways in the two-stage metabolomics analysis with the AD and MCI pathways generated in this systematic review. As shown in Figure 3, three pathways (including purine metabolism, arginine and proline metabolism, and the TCA cycle) revealed the common metabolic changes in AD and aging and provided explanations for why natural aging is closely related to the high incidence of AD. All of these pathways have been detected in brain tissue, CSF, serum, and plasma in the AD population. Additionally, two pathways (arginine and proline metabolism and the TCA cycle) were found to be duplicated between MCI VS. CN and aging-related pathways. In particular, arginine and proline metabolism were duplicated between MCI_AD VS. MCI and aging-related pathways, which indicated that this mechanism plays an important role in the progression from healthy to MCI and to AD in the natural aging population.

#### Metabolic Biomarkers of AD in the Natural Aging Population

Furthermore, we compared the top 30 metabolites of aging with the metabolites of AD and MCI reported in previous studies. After comparing Table 1 with Supplementary Tables 4, 5, 3 metabolites were identified that were related to both AD and aging, including 16-a-hydroxypregnenolone, stearic acid, and PC[16:0/22:5(4Z,7Z,10Z,13Z,16Z)], which can be considered metabolic biomarkers of AD in the natural aging population.

## Discussion

With aging, the probability of suffering from AD increases annually. Considering the dramatic aging of populations worldwide, it is of great importance to explore common mechanisms between AD and natural aging. The key finding of this study is the systematic comparison of the metabolic mechanisms of AD, MCI, and aging. We first revealed the metabolic mechanism and potential biomarkers of AD in a natural aging population.

Based on this independent metabolomics analysis in a two-stage cross-sectional study, we found metabolites and pathways associated with aging. Strikingly, pathways enriched by aging-related metabolites were consistent with the hypotheses of aging mechanisms in previous studies, such as oxidative stress (e.g., carnitine shuttle Yu et al., 2012), and inflammation (e.g., leukotriene metabolism). Additionally, in our work, pathways altered in the elderly were also related to various amino acid pathways. Previous detection of amino acids in the serum of normal healthy Japanese people revealed that the concentrations of aspartate, asparagine, and arginine increased with age in males, whereas the levels of tyrosine asparagine, arginine and proline increased with age in females, which together suggests that aspartate and asparagine metabolism and arginine and proline metabolism are related to aging (Kouchiwa et al., 2012). Furthermore, delayed degradation of plasma tyrosine (precursor of dopamine) in the elderly may influence cognition disruption during aging (Rudman et al., 1991; Berry et al., 2016). All of these metabolites also recurred in our study.

Previous studies have proven that oxidative stress plays a vital role in the progression of AD (Perry et al., 2002; Huang et al., 2016), as well as in MCI. The shared pathways between AD and MCI in our results reflected that MCI and AD have the same mechanisms. Glutathione, made up of cysteine, glycine and glutamate, was shown to have neuroprotective effects by reducing Aβ-related oxidative stress via 4-hydroxynonenal (Mark et al., 1997) and attenuating amyloid fibrillation (Wang et al., 2009). Furthermore, lipid metabolism is one of the most extensively implicated dysfunctions in the context of AD. Consistent with that, cholesterol and sphingolipid transport, saturated fatty acid metabolism and sphingolipid metabolism were disrupted in both AD and MCI. Moreover, disrupted amino acid pathways may be related to alterations of neurotransmitters. In our results, four pathways overlapped between AD VS. MCI and MCI_AD VS. MCI, including lysine metabolism, polyamine metabolism, catecholamine metabolism, and prostaglandin 2 biosynthesis and metabolism. Specific lysine residues within the microtubule-binding motif are the major sites of tau acetylation, which can inhibit tau function as a result of impaired tau–microtubule interactions and promote pathological tau aggregation (Cohen et al., 2011). Changes of polyamine metabolism in the brain influence the progression of AD through several mechanisms, such as the regulation of cholinergic neurotransmission (Mahajan et al., 2020). These pathway alterations indicated progressive changes in the patients from MCI to AD.

As shown in Figure 2, we compared the pathway results obtained in four biological samples from previous studies, and most of the pathway enrichment results are contained in plasma. Although there are some differences in the quantity of metabolites in different biological samples, most changes in the metabolites in the brain or CSF will be reflected in the plasma, in particular, all metabolic changes in the brain tissue of AD patients can be detected in plasma. Thus, plasma metabolites will most likely be the source of non-invasive markers. This provides us with the possibility of early screening of patients with MCI or AD in the aging population. From the mechanism point of view, perhaps AD related lesions in brain tissue may also be the result of accumulation or reduction of certain metabolites in plasma. We hope that our results can provide more inspirations for the study of relevant mechanisms. Moreover, we still cannot ignore the fact that more than 50% of metabolic changes in plasma cannot be evidenced in brain tissue and may be caused by other dietary or environmental exposures. Thus, the uniqueness of plasma AD markers should be given more attention in clinical studies.

As illustrated in Table 3, some fatty acids were found to be altered in both retrospective and prospective studies. In astroglia, palmitic acid may stimulate ceramide synthesis by secreting signaling molecules such as cytokines and nitric oxide, resulting in Aβ accumulation and tau hyperphosphorylation (Patil et al., 2007). Similarly, stearic, linoleic, and oleic acids were proven to be related to the accumulation of both Aβ and tau *in vitro* (Patil and Chan, 2005; Amtul et al., 2012). Five metabolites (palmitic acid, stearic acid, linoleic acid, glutamine, and oleic acid) in serum/plasma have also been confirmed in brain tissue, which suggests their powerful potential for the non-invasive diagnosis of AD. Moreover, arginine, creatine and histidine were observed in both retrospective and prospective studies. Considering that arginine and histidine were altered in both CSF and plasma/serum, these two metabolites may act as non-invasive biomarkers for the MCI population to monitor the progression from MCI to AD.

The novel findings in our study are the metabolic pathways and biomarkers related to both aging and AD. Regarding the three shared pathways between aging and AD, the TCA cycle did not attract our attention because it is such an extensive metabolic pathway altered in diverse physiological and pathological processes, which include the preclinical stage of AD (Atamna and Frey, 2007). Purine nucleoside phosphorylase (PNP) converts guanosine to guanine and inosine to hypoxanthine and is an important enzyme involved in purine metabolism. A study on astroglia reported a marked increase in PNP with aging (Zoref-Shani et al., 1995), while another study observed increased PNP activity in patients with AD (Alonso-Andres et al., 2018). Regarding arginine and proline metabolism, arginine is the central substance and serves as the only precursor of nitric oxide (NO). NO could react with superoxide (O_2_−) to produce peroxynitrite (ONOO−), and the latter is so active that it would experience cleavage and generate reactive oxygen/nitrogen species (ROS/RNS) (Yi et al., 2009), which could occur in the process of natural aging. In addition, the brain is much more vulnerable to nitroxidative stress than other tissues due to its high oxygen demand, weakened antioxidative ability and low proliferative trait of neurons, indicating that oxidative stress is involved in the initiation of AD in healthy individuals (Kern and Behl, 2009). Arginine could be metabolized to agmatine, which is involved in memory decline processes and can be found in both elderly and AD brain tissue (Liu et al., 2014). Arginine and proline metabolism contains several metabolic pathways we mentioned in the AD-related pathways, such glutathione, glycine, and polyamine metabolism. Evidence has shown that these pathways are related to aging (Minois et al., 2011; Hashizume et al., 2015; Weschawalit et al., 2017). We can see that arginine and proline metabolism has been shown to play a role in prospective studies of the progression from no disease to MCI and eventually AD; likewise, arginine and proline metabolism appears in the pathways related to natural aging.

From the results of direct comparison of metabolite lists of aging and AD, there are three metabolites that were found to be duplicated: 16-a-hydroxypregnenolone, stearic acid and PC[16:0/22:5(4Z,7Z,10Z,13Z,16Z)]. 16-a-hydroxypregnenolone is classified as a gluco/mineralocorticoid, a progestogin or aprogestogin derivative. Although no cytological mechanism studies have confirmed the role of 16-a-hydroxypregnenolone in aging and dementia, we observed that it was significantly associated with aging in our metabolomics analysis and altered in AD patients in population studies (Vankova et al., 2016). Thus, experimental confirmation based on *in vitro* studies is urgently needed.

Strikingly, studies have demonstrated the role of stearic acid and PC[16:0/22:5(4Z,7Z,10Z,13Z,16Z)] in the pathological process of AD. Together with a recent study demonstrating a close relationship between tau protein and inflammatory signaling in astrocytes (Lemoine et al., 2017), we assume a possible pathological process in astrocytes combining aging and AD via inflammatory and oxidative responses. Patil and Chan (2005) found that astroglia-mediated oxidative stress may be related to stearic and palmitic fatty acid-induced hyperphosphorylation of tau. Investigations have shown that in astrocytes, stearic acid promotes the release of inflammatory factors such as IL-6 and TNFα (Gupta et al., 2012). PC[16:0/22:5(4Z,7Z,10Z,13Z,16Z)] can be classified as PtdCho, which can be synthesized from cytidine diphosphate choline (CDP-choline) and diacylglycerol and contains long-chain polyunsaturated fatty acids, which are important components of neuron membranes. Wurtman (1992) proposed that choline was used to synthesize both acetylcholine (Ach) and PtdCho. Therefore, PtdCho could be taken to maintain the level of Ach when the body experiences a shortage of choline. Choline deficiency could occur in the contexts of both aging and AD, resulting in depletion of PtdCho and death of cholinergic neurons. This “autocannibalism” hypothesis partially explained the selective vulnerability of the cholinergic system and provided clues regarding PtdCho as a shared metabolite of natural aging and AD.

The limitations of our study are that we did not conduct *in vitro* experiments to verify the overlapping mechanisms between aging and AD. Although we used training and testing sets to screen out the aging-related metabolites that can be stably detected, our metabolomics research was a non-targeted test. It is necessary to further verify and analyze the sensitivity and specificity of metabolic markers based on targeted quantitative detection of a larger sample in a cohort population.

In conclusion, this study is the first to comprehensively compare metabolites and pathways between aging and AD by utilizing metabolomic measurement and systematic review. We proposed potential non-invasive biomarkers for AD diagnosis and MCI monitoring based on retrospective and prospective population studies. More importantly, we revealed the key role of arginine and proline metabolism in the progression from a healthy status to MCI to AD in a natural aging population. In particular, we provided potential metabolic markers (16-a-hydroxypregnenolone, stearic acid, and PC[16:0/22:5(4Z,7Z,10Z,13Z,16Z)] of AD diagnosis for future validation in a natural aging population.

## Data Availability Statement

The datasets presented in this study can be found in online repositories. The names of the repository/repositories and accession number(s) can be found in the article/Supplementary Material.

## Ethics Statement

The studies involving human participants were reviewed and approved by the Human Research and Ethics Committee of Harbin Medical University. The patients/participants provided their written informed consent to participate in this study.

## Author Contributions

FW and MW contributed to the study design, data interpretation, study supervision, and the acquisition of funding. YZ contributed to critical revision of the manuscript for important intellectual content. ZL and YY contributed to data processing, statistical analysis, and identification of differential metabolites. CP, KX, and YY contributed to questionnaire and sample collection. ZW, VD, and CX contributed to sample preparation and metabolomics detection. ZL, YY, and LL contributed to literature search, quality assessment of individual studies and data extraction. KX and QQ contributed to manuscript preparation. All authors contributed to review and revision of the manuscript.

## Conflict of Interest

The authors declare that the research was conducted in the absence of any commercial or financial relationships that could be construed as a potential conflict of interest.

## Footnotes

1 http://mummichog.org/

2 https://www.metaboanalyst.ca/

3 https://doi.org/10.6084/m9.figshare.13621664

## Supplementary Material

Supplementary Figure 1 Variable importance for the top 30 aging-related metabolites selected by random forest. The *y*-axis represents retention time and mass of metabolites. The *x*-axis represents IncNodePurity, which indicates the importance of the variable given by the classification model.

Supplementary Figure 2 Flow diagram of the literature search of metabolomics studies in Alzheimer’s disease using the PubMed, Cochrane and Embase databases.

Supplementary Figure 3 Description of the included studies’ basic information. **(A)** year of publication; **(B)** race of population; **(C)** study design; **(D)** different platforms used in different biosample analyses.

## Abbreviations

Ach: acetylcholine
AD: Alzheimer’s disease
Aβ: amyloid beta
BDNF: brain-derived neurotrophic factor
CNS: central nervous system
CDP-choline: cytidine diphosphate choline
DHA: Docosahexaenoic acid
ESI: electrospray ionization
CN: healthy controls
MS: mass spectrometry
MCI: mild cognitive impairment
NMR: nuclear magnetic resonance
NO: nitric oxide
QUADAS: Quality Assessment of Diagnostic Accuracy Studies
QC: quality-control
RSD: relative standard deviations.
