# Supplementary material for: High-Throughput Metabolomics for Discovering Potential Biomarkers and Identifying Metabolic Mechanisms in Aging and Alzheimer’s Disease
Source: Front Cell Dev Biol. 2021 Feb 25;9:602887. doi: 10.3389/fcell.2021.602887 (PMC7947003; doi:10.3389/fcell.2021.602887)
Supplement: Supplementary Figure 1 — Variable importance for the top 30 aging-related metabolites selected by random forest. The y-axis represents retention time and mass of metabolites. The x-axis represents IncNodePurity, which indicates the importance of the variable given by the classification model. [file Data_Sheet_1.PDF]

SUPPLEMENTARY MATERIAL FOR

# High-throughput metabolomics for discovering potential biomarkers and identifying metabolic mechanisms in aging and Alzheimer's disease

## 1. Supplementary Methods

### 1.1 Experimental protocol of metabolic profiling analysis for plasma by UPLC/Q-TOF-MS/MS

#### Chemicals and reagents

Formic acid was procured from Beijing Reagent Company (Beijing, China). Methanol and acetonitrile of HPLC grade were both procured from Honeywell Burdick & Jackson (Muskegon, MI). All regents and chemicals (HPLC grade) were available from commercial sources. Ultrapure water was purchased from Veolia Water Solutions & Technologies (France).

#### Plasma sample preparation

Samples were thawed at 4°C and vortexed for 1 min. We obtained 250 μl of plasma using a 2-ml centrifuge tube, followed by addiction of 750 μl of methanol, a 1 min vortex, and centrifugation performed at 12000 rpm for 10 min at 4°C. The upper phase of the mixture was collected and dried via nitrogen. Proteins were treated with a 350 μl mixture consisted of acetonitrile and water (1:2, v/v) for deproteinization, after which they were subjected to a 1 min vortex and centrifugation at 12,000 rpm for 10 min at 4°C. The collected supernatants were brought to the dried tubes for the dissolution of the residue. A final sample was obtained before a 1 min vortex, a 5 min stewing, and centrifuged at 12,000 rpm for 10 min at 4°C. Then, the metabolite-containing supernatants were placed in autosampler vials for metabolic measurement. Aliquots of 5 plasma was used as pooled quality-control (QC) sample to evaluate the repeatability and reliability of the platform, with which plasma samples were analyzed in two batches at regular intervals throughout the measurement run (n =15).

#### UPLC/Q-TOF-MS/MS analysis

Plasma metabolite separation was conducted using a UPLC system (ACQUITY UPLC; Waters Corp., USA) equipped with 1.7-μm BEH C18 column [ACQUITY (HSS); Waters Corp., Milford, MA, USA; 2.1 mm × 100 mm], maintained at 35°C. The mobile phase was performed using 0.1% formic acid waters (A) and acetonitrile (B). Each plasma sample was delivered at a volume of 2 μl and a flow rate of 0.35 ml/min. The elution gradient was 0.5 min (2% B), over 0.5 to 6.0 min (2% to 20% B), over 6.0 to 7.0 min (20% to 35% B), over 7.0 to 9.0 min (35% to 70% B), over 9.0 to 10.5 min (70% to 98% B), 2.0 min (98% B), 6.0 min (2% B). After establishing the initial settings, the column was equilibrated for 2.0 min. Samples were injected in a randomized order with acetonitrile run at regular intervals (n=4) as a blank solution.

Q-TOF MS/MS, incorporated with a mass spectrometer (Micromass Q-TOF mass pectrometer; Waters Corp., Manchester, UK), was carried out in electrospray ionization (ESI) interface under full scan monitoring mode (50~1000 m/z). The capillary voltage and source temperature of $ESI^-$ and $ESI^+$ were set to be 2800V, 100°C and 3000V, 110°C, respectively. The desolvation gas (nitrogen) flow was 650 L/h and the temperature was 320°C.In both modes, collision energy was 6 eV, sample cone voltage was 35 V, collision gas was selected as argon, nitrogen cone gas flow was 50 L/h, MCP detector voltage was 2400 V and acquisition rate was 0.4 s with 0.1 s interscan delay. Data were obtained in centroid MS mode using the lock spray, the frequency of whom was 10s. Metabolite mass was locked by application of 200 pg/ml leucine-enkephalin (554.2615 in $ESI^-$ and 556.2771 in $ESI^+$) and data were averaged over 10 scans.

**Data processing**

MarkerLynx software software coupled with Masslynx software (version 4.1 SCN714) was used to pretreat the LC-MS data, the latter of which aimed to recognize and align peak. The peak width was set at 5% height and 1 s, and peak-to-peak baseline noise was automatically calculated. Parameters of collection were as follows: 0.5-10.5 min and 0.1 min for range and tolerance of retention time, 50-1000 Da and 0.05 Da for range and tolerance of mass, 6.0 for noise elimination level, 80 for minimum intensity, and remove deisotope peak data. The intensity of ion was converted to the summed

one of each chromatogram after peak identification and alignment. The data pretreatment was accorded with the “80% rule.”

**Differential metabolites identification**

Metabolite annotation was performed by comparing the exact m/z values and MS/MS spectra with those in free online databases Human Metabolome Database (HMDB, http://www.hmdb.ca/). Additionally, the MassFragment application manager (MassLynx version 4.1, Waters) was applied to facilitate the MS/MS fragment ion analysis process via chemically intelligent peak-matching algorithms.

**1.2 Systematic review of metabolomics studies of human Alzheimer’s disease**

**Literature search strategy**

**Inclusion and exclusion criteria**

Previous published studies were judged to be eligible if the following items were achieved: 1) all patients were diagnosed as AD or MCI with standard criteria, and all healthy controls (CN) were confirmed to be free of AD or MCI; 2) Studies applied approaches of metabolomics such as GC-MS, LC-MS and NMR; 3) Metabolites or metabolic pathways were presented with complete names; 4) Studies focused on differentiated metabolites or metabolic pathways which were related to the progression of MCI or AD. In detail, biomarkers were identified by at least one of following six comparisons: AD VS. CN, MCI VS. CN, AD VS. MCI, CN_AD (healthy subjects who developed AD subsequently) VS. CN (healthy controls without diagnosed AD during follow-up), CN_MCI (healthy subjects who developed MCI subsequently) VS. CN (healthy controls without diagnosed MCI during the follow-up), as well as MCI_AD (patients of MCI who developed AD subsequently) VS. MCI (patients of MCI without diagnosed AD during follow-up). The exclusion criteria were as follows: 1) Genomics or proteomics studies; 2) Studies were performed on non-human models including animals, plants or cells; 3) Studies investigated pathogenesis or clinical evaluation of AD drugs; 4) Subjects mixed with other kinds of dementia, such as vascular dementia, preclinical AD.

**Data extraction from included studies**

We examined the selected studies in more detail and extracted relevant information, including study characteristics (year of publication, author details, study design, sample characteristics, metabolomics platform applied in different biosamples and sample size), as well as a summary of findings that comprised differentiated metabolites and pathways.

Two authors (Y.H. Y and Z.P. L) independently extracted the data and appraised the quality of each article. Divergence regarding quality evaluation or information extraction was dealt with discussion or judgment of senior investigators (F. W. and M.Q. W.).

**2. Supplementary Tables**

Supplementary Table 1. Demographic and clinical characteristics of population in training and testing sets

Supplementary Table 2. The characteristics and identified metabolites or pathways of included metabolomics studies.

Supplementary Table 3. Quality assessment of metabolomics studies included in this systematic review by QUADOMICS

Supplementary Table 4. Differential metabolites of Alzheimer's disease and their reported frequencies in previous studies

Supplementary Table 5. Differential metabolites of mild cognitive impairment and their reported frequencies in previous studies

Supplementary Table 6. Differential metabolites between Alzheimer's disease and mild cognitive impairment and their reported frequencies in previous studies

Supplementary Table 7. Metabolites related with Alzheimer's disease replicated in previous prospective studies

Supplementary Table 8. Metabolites related with the conversion from mild cognitive impairment to Alzheimer's disease in previous prospective studies

Supplementary Table 9. Pathways altered in mild cognitive impairment and Alzheimer's disease identified by direct extraction and pathway enrichment analysis

Supplementary Table 10. Pathways altered in Alzheimer's disease identified in brain tissue, CSF, plasma and serum

## 3. Supplementary Figures

**Supplementary Figure 1. Variable importance for the top 30 aging-related metabolites selected by random forest.** The y-axis represents retention time and mass of metabolites. The x-axis represents IncNodePurity, which indicates the importance of the variable given by the classification model.

**Supplementary Figure 2. Flow diagram of the literature search of metabolomics studies in Alzheimer's disease using the PubMed, Cochrane and Embase databases.**

**Supplementary Figure 3. Description of the included studies' basic information.** (A) year of publication; (B) race of population; (C) study design; (D) different platforms used in different biosample analyses.

**Supplementary Table 1. Demographic and clinical characteristics of training set and testing set**

| | Training set(n=119) | Testing set(n=64) | t / $\chi^2$ value | *P*-value |
|---|---|---|---|---|
| **Age(years, mean(sd))** | 58.66±11.21 | 58.98±10.84 | -0.186 | 0.852 |
| **Range (min-max)** | 32~82 | 25~85 | | |
| **Gender(F/M)** | 48/71 | 31/33 | 0.80762 | 0.3688 |
| **BMI (kg/m2, mean(sd))** | 24.10±3.30 | 23.93±4.03 | 0.289 | 0.773 |
| **Education [a] (NO, (%))** | | | | |
| Primary school or below | 38(31.93) | 23(35.94) | | |
| Junior high school | 33(27.73) | 16(25) | | |
| Senior high school | 18(15.13) | 8(12.5) | | |
| College and above | 20(16.81) | 10(15.63) | | |
| Unknown | 10(8.4) | 7(10.94) | 0.84132 | 0.9328 |
| **Tobacco Smoking [b] (NO, (%))** | | | | |
| Yes | 43(36.13) | 28(43.75) | | |
| No | 48(40.34) | 34(53.13) | 0.008022 | 0.9286 |
| **Alcohol drinking [c] (NO, (%))** | | | | |
| Yes | 41(34.45) | 27(42.19) | | |
| No | 50(42.02) | 35(54.69) | 0.00033898 | 0.9853 |
| **Chronic diseases [d] (NO, (%))** | | | | |
| No | 67(56.3) | 43(67.19) | | |
| Yes | | | | |
| Diabetes | 17(14.29) | 3(4.69) | | |
| Hypertension | 27(22.69) | 5(7.81) | | |
| Heart disease | 22(18.49) | 6(9.38) | 0.46382 | 0.793 |
| **Exercise [e] (NO, (%))** | | | | |
| Yes | 34(28.57) | 18(28.13) | | |
| No | 50(42.02) | 34(53.13) | 0.25194 | 0.6157 |

[a]Education, missing for 10 in training set and 7 in testing set.

[b]Smoking, missing for 28 in training set and 3 in testing set.

[c]Drinking, missing for 28 in training set and 3 in testing set.

[d]Chronic diseases, missing for 6 in training set and 3 in testing set.

[e]Exercise, missing for 35 in training set and 12 in testing set.

**Supplementary Table 2. The characteristics and identified metabolites or pathways of included metabolomics studies.**

| Study First Author [ref]Year | Study Type | Number of Subjects | Match or Not | Sample Source | Sample Type | Platforms | Metabolites |
|---|---|---|---|---|---|---|---|
| Varma(1)2018 | Case-control study | 15 AD<br>15 ASYMAD<br>14 CN | No | BLSA | brain tissue | FIA-MS/MS；<br>HPLC-MS/MS | Arginine, Propionylcarnitine, lysoPC a C17:0, lysoPC a C18:0, PC aa C38:4, PC aa C40:4, PC aa C40:5 , PC aa C40:6, PC ae C34:0, PC ae C34:2 , PC ae C36:0, PC ae C36:3 , PC ae C36:4 , PC ae C40:1, PC ae C42:3, Serotonin, Spermidine, SM C16:0, SM C16:1, SM C18:1 , SM C24:1, SM C26:1, (SM (OH) C14:1, SM (OH) C22:1, SM (OH) C22:2, SM (OH) C24:1, H1 |
| Marksteiner(2) 2018 | Nested case-control study | 30 CN<br>20 MCI<br>30 AD | No | Hall/Tirol State Hospital, Austria | plasma | LC-MS/MS | glycochenodeoxycholic acid, glycodeoxycholic acid, glycolithocholic acid, lithocholic acid, lithocholic acid |
| Pan(3)2017 | Case-control study | Plasma:<br>10 AD<br>10 CN<br>Brain tissue:<br>10 AD<br>10 CN | Yes | Plasma:<br>the Belfast City Hospital<br>brain tissue:<br>Brains for Dementia Research (BDR) initiative | plasma and brain tissue | LC-MS/MS | cholic acid, taurocholic acid |
| Oresic(4)2017 | Case-control study | 37 AD<br>46 CN | No | Florida Hospital's NPH program | serum | GC-GC-TOFMS | Glutamic acid |
| Lau(5)2017 | Case-control study | 20 AD<br>20 PD<br>20 CN | No | Kyungpook National University Hospital | exhaled breath samples | GC-MS and in-house fabricated exhaled breath sensor systems | Acetamide, Siloxanes, Cyclopentasiloxane, Cyclotrisiloxane, Triphenyl phosphate, 1-phenantherol, ethyl 3-cyano-2,3-bis (2,5,-dimethyl-3-thienyl)-acrylate |

| | | | | | | | |
|---|---|---|---|---|---|---|---|
| Proitsi[6]2015 | Case-control study | 36 AD<br>48 MCI<br>40 CN | Yes | Dementia Case Register (DCR) at King's College London and the EU funded AddNeuroMed study | plasma | UPLC-MS | ChE 32:0, ChE 34:0, ChE34:6, ChE 32:4, ChE 33:6, ChE 40:4, Cholesterol, |
| Oberacher[7]2017 | Nested case-control study | training set:<br>21 AD<br>15 MCI<br>18 CN<br>validation set:<br>9 AD<br>6 MCI<br>11 CN<br><br>blinded follow up conversion study：<br>7 AD<br>3 CN | No | Hall/Tirol 120 State Hospital, Austria | platelets | FIA-MS/MS | PC aaC32:0, PC aeC32:2, PC aeC34:1, SM(OH) C14:1, PC ae C40:4, PC aeC32:2, PC aaC36:5, PC aeC34:1, lysoPCaC18:1, lysoPC aC16:0, SM(OH) C14:1 |
| de Leeuw [8]2017 | Case-control study | 127 AD<br>121 CN | No | Amsterdam Dementia Cohort | plasma | UPLC-MS;<br>GC-MS | 2-Aminoadipic , TG(51:3), 3-Hydroxyisovaleric acid , Tyrosine , TG(54:6), TG(50:4) , S-3-Hydroxyisobutyric acid , TG(56:8) , Methyldopa, 8-iso-PGF2a , TG(48:3) , O-Acetylserine , TG(48:2), Methylmalonic acid , TG(46:2), Valine , TG(50:3) Lipids, TG(52:4) , TG(52:5) , TG(56:7) , TG(48:0) , Ornithine, SM(d18:1/23:0) , SM(d18:1/20:1) , TG(48:1) , TG(58:10) , 2-Aminoadipic acid , Tyrosine, Lysine, TG(48:2) , TG(51:3) , TG(54:6) , TG(50:3) , TG(50:2) , TG(50:1) , Leucine , LPC(18:1) , TG(46:2) , TG(50:0) , |

| | | | | | | | |
|---|---|---|---|---|---|---|---|
| | | | | | | | TG(52:3) , TG(51:2) , Isoleucine , 2-hydroxybutyric acid , TG(51:1), TG(52:1), Proline, TG(54:5), TG(56:7), PGD2, TG(46:1), PC(O-44:5) , LPA C14:0 , PC(O-34:1) , LPC(20:4) , SM(d18:1/24:2) , 8,12-iPF2a IV , TG(46:0), 5-iPF2a VI , TG(52:2) , SM(d18:1/16:0), Ornithine , Histidine , PGD2 , Methyldopa , NO2-aLA (C18:3) , Serine , Arginine , SM(d18:1/23:0), Glyceric acid , Lysine , Glycolic acid , cLPA C18:0 , SM(d18:1/18:0) , LPA C22:4 , LPA C16 , 3-Methoxytyramine , 2,3-dinor-8-iso-PGF2a , PC(O-34:3) , Cis-Aconitic acid , LPA C14:0, PC(O-36:6) , PC(O-38:6) , Putrescine , Homoserine , TG(O-50:0), Carnosine , Sarcosine, O-Acetylserine, PE(38:2) , Gamma-glutamylalanine, 8-iso-PGF2a (15-F2t-IsoP) , LPA C18:3 , Uracil , Citrulline , Histamine , NO2-OA (C18:1) , S-3-Hydroxyisobutyric acid, 2-Aminoadipic acid, TG(50:3), TG(50:1), TG(52:5), Isoleucine, LPC(18:1), TG(52:1) , SM(d18:1/20:1), 1-Methylhistidine , 5-iPF2a VI, TG(51:2), TG(58:9), Histidine, DG(36:3) , Phenylalanine, SM(d18:1/24:2), TG(46:0) , Methionine , LPA C20:1 , TG(48:3), TG(52:3), TG(58:10), TG(58:9) , Methylmalonic acid, TG(50:0), SM(d18:1/25:0) , LPA C18 , Glyceric acid, 8-iso-PGF2a (15-F2t-IsoP), TG(O-50:0) , TG(48:0), Serine, Putrescine, 3-Methoxytyramine, cLPA C18:1 , PE(O-38:5) , LPC(20:4), Gamma-glutamylalanine , 2,3-dinor-8-iso-PGF2a, Cysteine , Carnosine, PC(O-34:2) , SM(d18:1/25:0) |
| Zhu[9]2016 | Case-control | 7 AD | No | Karolinska Institutet | brain | LC-MS-MS | RvD5, MaR, PD1, PGD2, |

| | | | | | | | |
|---|---|---|---|---|---|---|---|
| | study | 7 CN | | | tissue | | |
| Xu[10]2016 | Case-control study | 9 AD<br>9 CN | Yes | New Zealand Neurological Foundation HBB | brain tissue | GC--MS | Glucose, Glucose-6-phosphate, Sorbitol, Fructose, Fructose-6-phosphate, Pentonic acid A, Pentonic acid B, Arabinose, Ribose-5-phosphate, Erythronic acid, Butanediol , β-Hydroxybutyric acid , Lactic acid , 2-hydroxy-3-methylbutyric acid , Threitol , Xylitol , Disaccharide , N-acetylglucosamine , Myo-inositol , Myo-inositol-1-phosphate , Glycerol , Glycerol-2-phosphate , Glycerol-3-phosphate , Glyceric acid , Citric acid , Malic acid , Fumaric acid , Ornithine , Urea , N-acetylglutamic acid , Creatinine , Proline , Lysine , Glycine , Serine , Threonine , Cysteine , beta-Alanine , Aspartic acid , N-acetylaspartic acid , Glutamic acid , GABA , 4-hydroxybutyric acid , Phenylalanine , Tryptophan , Adenine , Uracil , Adenosine-5-monophosphate , Guanosine , Hypoxanthine , Ethanolamine , Methyl-phosphate , Phosphoric acid , 2-Hydroxyglutaric acid , Ascorbic acid |
| Paglia[11]2016 | Case-control study | 21 AD<br>19 CN | Yes | Banner Sun Health Research Institute | brain tissue | UPLC-HILIC-MS and LC-MS | Acetylaspartic acid, Aspartic acid, Alanine, Asparagine, Pyruvate, Glutamic acid, Glutamine, Succinic acid, Arginine, Proline, SAMe, Hydroxyproline, Acetylglutamic acid, Serine, Cystine, Methionine, SAH, Threonine, Choline, Tryptophan, Pentose 5-phosphate, ADP-ribose, ADP, AMP, GMP, Guanosine, IMP, Uric acid, Xanthine, Xanthosine, Hypoxanthine, Inosine, Valine, Pantothenic acid, aspartate, malate, citrate, glutamate<br>Arginine and proline metabolism, Alanine, aspartate and glutamate metabolism, Glycine, serine and threonine |

| | | | | | | | |
|---|---|---|---|---|---|---|---|
| | | | | | | | metabolism, Purine metabolism, Cysteine and methionine metabolism, Pantothenate and CoA biosynthesis |
| Nasaruddin [12]2016 | Case-control study | 14 AD<br>15 CN | Yes | Newcastle Brain Tissue Resource | brain tissue | GC-MS | Pentadecanoic acid, Palmitic acid, Palmitoleic acid, Heptadecanoic acid, Stearic acid, Elaidic acid, all-cis-11,14,17-eicosatrienoic acid, all-cis-13,16-docosadienoic acid, Docosahexanoic acid |
| Casanova[13]2016 | Nested case-control study | The Baltimore Longitudinal Study of Aging (BLSA):<br>93 AD<br>99 CN<br>Age, Gene/Environment Susceptibility-Reykjavik Study (AGES-RS):<br>100 AD<br>100 CN | Yes | BLSA and AGES-RS | serum | FIA-MS/MS; HPLC-MS/MS | Propionylcarnitine, Glutarylcarnitine or Hydroxyhexanoylcarnitine, Creatinine, Methionine, Ornithine, Serine, Taurine, Threonine, Glucose, PC aa C36:4, PC aa C38:4, PC ae C30:2, PC ae C42:5, PC ae C44:4, Acetylcarnitine, C5(Isovalerylcarnitine / 2-Methylbutyrylcarnitine / Valerylcarnitine), C5:1-DC(Glutaconylcarnitine / Mesaconylcarnitine), Citrulline, Symmetric dimethylarginine, lysoPC a C16:1（Lysophosphatidylcholine with acyl residue C16:1）, lysoPC a C18:1（Lysophosphatidylcholine with acyl residue C18:1）, PC aa C32:3, PC aa C34:3, PC aa C36:3 |
| Gonzalez-Dominguez [14]2016 | Case-control study | 75 AD<br>17 MCI<br>45 CN | Yes | Neurological Service of Hospital Juan Ramón Jiménez | serum | UPLC-MS | LPE (16:0), LPE (18:2), LPC (16:1), LPC (16:0), LPC (O-18:0), LPC (20:5), LPC (22:6), LPC (22:5), PE(16:0/18:0), PC(16:1/16:0), PC(16:0/16:0), PPC(16:0/18:2), PC(15:0/18:2), PC(15:0/18:1), PPE(16:0/22:6), PPE(18:1/20:4), PC(16:0/18:2), PC(16:0/20:3), PC(16:1/22:6), PC(18:0/20:4), PC(20:4/20:4), PC(18:1/22:6), PC(20:4/22:6), sphingosine-1-phosphate, CER(d18:1/16:0), SM(d18:1/12:0), SM(d18:1/14:0), SM(d18:1/16:0), SM(d18:1/18:2), SM(d18:1/18:1), |

| | | | | | | | |
|---|---|---|---|---|---|---|---|
| | | | | | | | SM(d18:1/18:0), Hex-CER(d18:1/16:0), Hex-CER(d18:1/18:0), SULF(d18:1/18:0), Lac-CER(d18:1/14:0), Lac-CER(d18:1/16:1), Lac-CER(d18:1/16:0), Monopalmitin, MG(18:0), palmitoyl-carnitine, inoleyl-carnitine, oleylcarnitine, stearoyl-carnitine, histidine, PAG, oleamide, PREGS |
| Ciavardelli, D.[15]2016 | Case-control study | 35 AD<br>38 MCI<br>34 CN | Yes | memory clinic of the Santa Lucia Foundation (Rome, Italy) | plasma | GC-MS; LC-MS | octanoylcarnitine , decenoylcarnitine, decanoylcarnitine , dodecenoylcarnitine, lauroylcarnitine , tetradecenoylcarnitine, acetylcarnitine , 2-hydroxybutyric acid, tetradecadienoylcarnitine, oleylcarnitine, free carnitine |
| Yoshida [16]2015 | Case-control study | 32 AD<br>22 MCI<br>74 CN | Yes | unknown | urine | LC-MS/MS | creatinine |
| Vankova[17]2016 | Case-control study | 16 AD<br>22 CN | Yes | unknown | serum | GC-MS | Pregnenolone sulfate, 17-Hydroxypregnenolonea , 17-Hydroxyprogesteronea, 20α-Dihydroprogesterone , Androstenedione , Allopregnanolone , Allopregnanolone sulfate, Isopregnanolone , Pregnanolone , Conjugated pregnanolone, Conjugated 5ß-pregnane-3α,20α-diol, Conjugated 5α-androstane-3ß,17ß-diol , Pregnenolone, 17-Hydroxypregnenolone, 16α-Hydroxypregnenolone, Cortisol, Allopregnanolone, Isopregnanolone, Pregnanolone, Androsterone sulfate, Epiandrosterone sulfate, Epietiocholanolone sulfate, 17-hydroxyprogesterone, 16α-hydrox-yprogesterone, cortisol, Epiandrosterone sulfate, epietiocholanolone sulfate, conjugated 5ß-androstane-3α,17ß-diol, pregnenolone, pregnenolone sulfate, 16α-hydroxypregnenolone, 16α-hydroxyprogesterone, |

| | | | | | | | |
|---|---|---|---|---|---|---|---|
| | | | | | | | conjugated pregnanolone , 5α-Androstane-3b,17ß-diol sulfate, 5ß-Androstane-3α,17ß-diol sulfate, Epiandrosterone sulfate |
| Cho[18]2006 | Case-control study | 20 AD<br>20 CN | Yes | Gongju Geriatric Hospital | plasma | Column-switching HPLC with MS spectrometry | DHEAS |
| Koal [19]2015 | Case-control study | 50 AD-Like<br>50 CN | No | unknown | CSF | FIA-MS/MS ;<br>HPLC-MS/MS | SM (d18:1/18:0), SM (d18:1/18:1), SM(OH)C14:1, PC aa C30:0, PC aa C32:0, PC aa C34:1, PC aa C34:4, PC aa C36.1, PC aa C36:6, PC aa C38:4, PC aa C38:5, PC aa C38:6, PC ae C30:1, PC ae C34:0, PC ae C38:2, lyosPC a C16:0, C10:2, C16-OH, C3-DC-M/C5-OH, Ala, Gln, Creatinine |
| Klavins[20]2015 | Case-control study | 43 AD<br>33 MCI<br>35 CN | No | the memory clinics at the Department of Psychiatry of Innsbruck Medical University and Hall in Tirol State Hospital | plasma | AbsoluteIDQ p180 Kit (Biocrates Life Sciences) | PC aa C34:4, PC aa C38:3, PC aa C40:5, PC aa C36:6, PC aa C40:6, lysoPC a C18:1, lysoPC a C18:2, glycine, valine |
| Gonzalez-Dominguez [21]2015 | Case-control study | 30 AD<br>30 CN | No | Neurologic service of Hospital Juan Ramón Jiménez (Huelva, Spain) | serum | FIA-APPI-QTOF MS | Palmitoleamide, Palmitamide, Linolenamide, Linoleamide, Oleamide, Stearamide, CER(d18:1/16:1), CER(d18:1/16:0), CER(d18:1/18:1), CER(d18:1/18:0), CER(d18:1/18:0)-1P, CER(d18:1/24:1), DAG(14:1/16:0), DAG(16:0/18:3), DAG(16:0/18:1), DAG(18:3/18:3), DAG(18:3/18:2), DAG(16:0/20:4), DAG(18:2/18:1), Palmitoleicacid, Palmitic acid, Oleic acid, Urea, Alanine, Taurine, Picolinic acid, Creatine, Malic acid, Dopamine, Serotonin |
| Cui[22]2015 | Case-control study | 33 AD<br>31 CN | No | Centre of Harbin Elderly Care Service in Heilongjiang Province of | serum | GC-MS | C14:0, C16:0, C18:0, C16:1, C18:1, C18:2, γ-C18:3, C20:2, C22:6, C18:3 |

| | | | | | | | |
|---|---|---|---|---|---|---|---|
| | | | | northern China. | | | |
| Ansoleaga[23] 2015 | Case-control study | 16 AD<br>4 CN | No | Institute of Neuropathology HUB-ICO-IDIBELL Biobank | brain tissue | LC-MS | guanine, dGMP, glycine, xanthosine, inosine diphosphate, deoxyguanosine;<br>purine metabolism |
| Wisniewski[24] 2013 | Case-control study | 9 AD<br>10 CN | Yes | BioRepository Center of NYU-Langone Medical Center | brain tissue | LC-MS | desmosterol |
| Gonzalez-Dominguez [25] 2014 | Case-control study | 19 AD<br>17 CN | No | Neurologic Service of Hospital Juan Ramón Jiménez (all residents in the province of Huelva) | serum | UPLC-ESI-QTOF-MS;<br>UPLC-ICP-MS | LPE(16:0), LPE(18:2), LPE(18:1), LPC(16:0), LPPC(18:1), LPPC(18:0), LPC(18:2), LPC(18:1), LPC(18:0), LPC(20:5), LPC(22:6), PC(16:1/16:1), PC(16:1/16:0), PC(16:0/16:0), PC(16:1/18:3), PC(16:0/18:3), PC(16:0/18:2), PC(16:0/18:1), PC(16:0/18:0), PC(16:0/20:5), PC(18:2/18:2), PC(18:2/18:1), PC(18:1/18:1), PC(18:0/18:0), PC(18:2/20:5）, PC(16:0/22:6), PC(16:0/22:5), PC(18:1/20:4), PC(18:1/20:3), PC(18:0/20:3), PC(18:0/22:6), PE(16:1/20:5), PE(16:0/20:5), PE(18:1/20:5), PE(18:1/20:4), PE(18:2/22:6), PE(18:1/22:6), PE(18:0/22:6), PE(22:6/22:5), PE(22:5/22:4), PPE(16:0/20:5), PPE(16:0/20:4), PPE(18:1/18:2), PPE(18:1/18:1), PPE(16:0/22:6), PPE(18:1/20:4), PPE(18:0/20:4), PPE(18:1/22:6), PPE(18:0/22:6), PPC(16:0/22:6), PPC(18:1/20:4), PPC(18:0/20:4), PPC(18:1/22:6), PPC(18:0/22:6) |
| Gonzalez-Dominguez [26] 2014 | Case-control study | 42 AD<br>14 MCI<br>37 CN | Yes | Neurological Service of Hospital Juan Ram´on Jim´enez | serum | CE-ESI-TOF-MS | Choline, Creatinine, Creatine, Asparagine, Proline betaine, Methionine, Histidine, Carnitine, Gly Val, N-acetyl-spermidine, Asymmetric dimethyl-Arginine, Leu Pro, C5-Carnitine, Hcy-Cys, Glu Gln, C8-carnitine, Phe Phe, C10:1-carnitine, |

| | | | | | | | |
|---|---|---|---|---|---|---|---|
| | | | | | | | C10:0-carnitine, Tripeptide |
| Liu[27]2014 | Case-control study | 20 AD<br>20 MCI<br>20 CN | No | Shengjing Affiliated Hospital, China Medical University | plasma | UPLC-MS | L-phenylalanine, L-phenylalanine fragment, Tryptophan fragment, Progesterone, Phytosphingosine, Dihydrosphingosine, LPC C 18:2, LPC C 20:4, LPC C 16:0, LPC C 18：1, LPC C 18：0, |
| Gonzalez-Dominguez [28]2014 | Case-control study | 22 AD<br>18 CN | No | Patients were newly diagnosed of sporadic Alzheimer's disease by the Neurologic Service of Hospital Juan Ramón Jiménez | serum | DIMS | LPC(16:0), LPC(18:1), LPC(18:0), LPC(20:5), PPE(16:0/22:6), PPE(18:1/20:4), PPE(18:0/20:4), PPE(18:1/22:6), PPE(18:0/22:6), PC(16:0/18:3), PC(16:0/18:2), PC(16:0/18:1), PC(16:0/18:0), PC(16:0/20:5), PC(18:2/18:2), PC(18:2/18:1), PC(18:2/18:0), PPC(16:0/22:6), PPC(16:0/22:5), PPC(18:1/22:6), PC(16:0/22:6), PC(18:1/20:4), PC(18:0/22:6), Caprylic acid, Capric acid, Lauric acid, Myristic acid, Palmitoleic acid, Palmitic acid, Linoleic acid, Docosahexaenoic acid, Leukotriene B4, Prostaglandin, Choline, Valine, Creatine, Glutamine, Glutamate, Dopamine, Histidine, Carnitine, Arginine, N-acetyl glutamine, Glucose, GPCh |
| Trushina E[29]2013 | Case-control study | 15 AD<br>15 MCI<br>15 CN | No | MCSA and ADRC | plasma and CSF | UPLC-ToF-MS | Methylglyoxal, Lactaldehyde, 2-Pyrrolidinone , Acetoin, Succinic anhydride, Indole, Vigabatrin, DL-Ornithine, N-Acetylarylamine, Urocanic acid, 1-Aminocyclohexanecarboxylic acid, Propionylglycine methyl ester, 2-oxo-4-hydroxy-hexanoic acid, 5,7-nonadienoic acid, 2-Methylbutyrylglycine , Phenylalanine, Terephthalic acid, ACECLIDINE, Hippuric acid, 2,4-DINITROPHENOL, Indoleacrylic acid, 8-Amino-7-oxononanoate, Asymmetric dimethylarginine, PYROGALLIN, Tryptophan, 10-hydroxy-11-dodecenoic |

| | | | | | | | |
|---|---|---|---|---|---|---|---|
| | | | | | | | acid, 12-amino-dodecanoic acid, EUPATORIOCHROMENE, 9,12-dioxo-dodecanoic acid, Glu Ser , 3E,13Z-octadecadien-1-ol, Ser Asp Gly, 1-Methyladenosine, 5-octadecylenic acid - 1.9778987, 1,3-DIPROPYL-8-CYCLOPENTYLXANTHINE [DPCPX], Isorhamnetin, Val Ser Lys , Anandamide (20:2, n-6), 1-octadecanoyl-rac-glycerol, Testosterone sulfate - 0.8743172, Cys Tyr Cys, 3beta,4beta-Dihydroxy-5beta-cholan-24-oic Acid, 2beta,3beta-Dihydroxy-6-oxo-5alpha-chol-7-en-24-oic Acid, Trp Gly Phe, Met His Lys, (24S)-1alpha,24-dihydroxy-22-oxa-20-epivitamin D3 / (24S)-1alpha,24-dihydroxy-22-oxa-20-epicholecalc, 3alpha,6alpha,12alpha-Trihydroxy-7-oxo-5beta-cholan-24-oic Acid, 25-hydroxy-26,27-dimethylvitamin D3 / 25-hydroxy-26,27-dimethylcholecalciferol, (17Z)-1alpha,25-dihydroxy-26,27-dimethyl-17,20,22,22,23,23-hexadehydrovitamin D3 / (17Z)-1alpha,25-d, 27-nor-5b-cholestane-3a,7a,12a,24,25-pentol, (23R,25R)-1alpha,25-dihydroxyvitamin D3 26,23-lactone / (23R,25R)-1alpha,25-dihydroxycholecalciferol, Glycoursodeoxycholic acid, Ritodrine glucuronide, Met Trp Gln, 6alpha-Hydroxycastasterone , Netilmicin, 1beta,25-dihydroxy-2beta-(3-hydroxypropoxy)vitamin D3 / 1beta,25-dihydroxy-2beta-(3-hydroxypropoxy)c, 1-Hydroxyvitamin D3 3-D-glucopyranoside, Biliverdin IX , |

| | | | | | | | |
|---|---|---|---|---|---|---|---|
| | | | | | | | Bilirubin, GlcAbeta-Cer(d18:1/18:0), GPEtn(18:1(11Z)/18:1(9Z))[U] , GPEtn(20:4(5Z,8Z,11Z,14Z)/20:4(5Z,8Z,11Z,14Z))[U], GPGro(17:0/20:4(5Z,8Z,11Z,14Z)), GPEtn(O-16:0/22:5(4Z,7Z,10Z,13Z,16Z)), Acetoacetic acid, Deoxyadenosine, Fumaric acid, Proline, Pyruvic acid, Pyroglutamic acid , 2-Methylbutyrylglycine, 2-Methyl-3-ketovaleric acid, 2-Furoic acid, N-Acetyl-a-neuraminic acid, Dehydroascorbic acid , 5-(hydroxymethyl)- 2-Furancarboxylic acid , Testosterone sulfate, Diacetyl , Diethanolamine, Ne-Methyl-L-lysine, Lecanoric acid, (S)-2-Hydroxyglutarate, 4-Hydroxy-L-threonine, Acetazolamide, Isoniazid, Pyrimethamine, Pirenzepine, ETHOPROPAZINE, L-Aspartic acid b-semialdehyde, COTARNINE, 8-Amino Caprylic acid, Bis (2-hydroxypropyl) amine, Allocortol, diethyltoluamide, 20alpha-Dihydroprednisolone, Gualenate (Sodium Gualenate), N,N-Didemethylchlorpromazine, MYCOPHENOLIC ACID, TUBAIC ACID, AMBELLINE, 7alpha-(Thiomethyl)spironolactone, L-Glutamic acid dibutyl ester, BERGENIN, CITROPTEN, O-Desmethylnaproxen , Embelin , Phenoperidine, Idebenone Metabolite (Benzenehexanoic acid, 2,5-dihydroxy-3,4-dimethoxy-6-methyl-) + 5.8371787, DIALLYL SULFIDE, 4-Methylumbelliferyl beta-D-glucuronide, 1,3-Glyceryl dinitrate, Glycolate, |

| | | | | | | | |
|---|---|---|---|---|---|---|---|
| | | | | | | | METHYL 7-DESHYDROXYPYROGALLIN-4-CARBOXYLATE, Ethosuximide, 7-hydroxy Tetranor Iloprost, Oxaloglutarate, Methoxsalen Metabolite, Met Glu Lys, Met Ala His , Iridotrial glucoside, Imidazolone, EPICATECHIN PENTAACETATE, dodecanamide, clavirin I, a-hydroxybutyrate, 6,7-DICHLORO-3-HYDROXY-2-QUINOXALINECARBOXYLIC ACID, 2-ISOPROPYL-3-METHOXYCINNAMIC ACID, 10-hydroxy-8E-Decene-2,4,6-triynoic acid, (R)-3-Methyl-2-oxobutanoate, (24S)-1alpha,24-dihydroxy-22-oxavitamin D3 / (24S)-1alpha,24-dihydroxy-22-oxacholecalciferol + 2.8750935, (22R)-1alpha,22,25-trihydroxy-23,24-tetradehydro-24a,24b-dihomo-20-epivitamin D3 / (22R)-1alpha,22,2, (22E,24E)-1alpha,25-dihydroxy-22,23,24,24a-tetradehydro-24a-homovitamin D3 / (22E,24E)-1alpha,25-dih, Azatadine, N-Acetylcadaverine, VD 2656, a-hydroxyisovalerate, Hydroxyhydroquinone, (22R)-1alpha,22,25-trihydroxy-26,27-dimethyl-23,24-tetradehydro-24a-homo-20-epivitamin D3 / (22R)-1a + 1.1406572, L-2-Aminoadipic acid, p-Hydroxyaniline, 5-oxo-7-decynoic acid, (23R)-1alpha,23,25-trihydroxy-24-oxovitamin D3 / (23R)-1alpha,23,25-trihydroxy-24-oxocholecalciferol, Ala Leu, Estradiol valerate, Trp Ala Ile, Diaminopimelic acid, |

| | | | | | | | |
|---|---|---|---|---|---|---|---|
| | | | | | | | N4-Acetylsulfapyridine, 1alpha,25-dihydroxy-11beta-phenylvitamin D3 / 1alpha,25-dihydroxy-11beta-phenylcholecalciferol, epsilon-Caprolactam , N-arachidonoyl D-serine, Purine, Phe Val Val, GPSer(16:0/18:1(11Z)), 1alpha,25-dihydroxy-3-deoxy-3-thiavitamin D3 / 1alpha,25-dihydroxy-3-deoxy-3-thiacholecalciferol, Glucosamine , Thr Ser Gln, Ophiobolin A, 1alpha,25-dihydroxy-22-oxavitamin D3 3-hemiglutarate/ 1alpha,25-dihydroxy-22-oxacholecalciferol 3-he, Glu Ser + 1.2297873, QUERCETIN TETRAMETHYL (5,7,3',4') ETHER, Visnagin, clavulone I - 10.898006, Loroxanthin ester/ Loroxanthin dodecenoate, Penicillin G, Ala Thr Pro, Isoquinoline, 5-NITRO-2-PHENYLPROPYLAMINOBENZOIC ACID [NPPB] + 1.8134243, 20-oxo-heneicosanoic acid, Arginine, Indole-3-ethanol , Ile Ser Lys, 5H-Oxireno[4,5]furo[3,2-g][1]benzopyran-5-one, 1a,8b-dihydro-3-methoxy-, Creatinine, Dimethylglycine, 4,7,10,13,16-docosapentaenoic acid, 1-Phenyl-2-(diethylamino)-1-propanol, 4-Hydroxybenzyl cyanide , EMODIC ACID - 4.837887, N-methyl-Gabapentin, Traumatic acid, Acetamide, PTERIN-6-CARBOXYLIC ACID, Asp Asn Glu, 1-eicosanoyl-rac-glycerol, glyceraldehyde, TEGASEROD, 3,6-octadecadiynoic acid, KARANJIN, Acenocoumarol, 5-(hydroxymethyl)- |

| | | | | | | | |
|---|---|---|---|---|---|---|---|
| | | | | | | | 2-Furancarboxylic acid, BEPRIDIL, 4-Pyridoxic acid , 5-octadecylenic acid, 7,12-Dioxo-5beta-cholan-24-oic Acid, Rimiterol, N'-5Z,8Z,11Z,14Z-eicosatetraenoyl-N''-diethyl-ethylenediamine, 4-dodecynoic acid, Acetoin , 4-undecynoic acid, 5beta-Androstan-3alpha-ol-17-one sulfate - 7.384574, Leu Glu Gln, N(pai)-Methyl-L-histidine, 6-hydroxy-2-hexynoic acid, 8R-hydroxy-9Z-octadecenoic acid, guaifenesin, Biliverdin IX, Queuine, Propionylglycine methyl ester , (22S)-1alpha,22,25-trihydroxy-23,24-tetradehydro-24a,24b-dihomo-20-epivitamin D3 / (22S)-1alpha,22,2, GPA(21:0/22:6(4Z,7Z,10Z,13Z,16Z,19Z)), Citraconic acid, threo-Isocitric acid , Pyruvic acid , Methionine, Granisetron metabolite , p-Aminobenzoic acid , N-Didesethylquinagolide, dihydro-alpha-ergocryptine, Pro Pro, L-Alanine n-butyl ester, Loratadine, bicyclo-PGE2, 4'-Hydroxyminoxidil, 7,8-Didehydroastaxanthin, ,4-Dihydroxyphenylpropionic acid, 3-Aminopropanal, S-(p-Azidophenacyl)glutathione, 3-(a-Naphthoxy)lactic acid, Adriamycinone, Valine, Isoacitretin , Bifemelane (M4), Acyclovir, 5-Hydroxytryptophan, Mebeverine, 2-Pyrrolidinone, Taurine, 2-Amino-3-methyl-1-butanol, 13,14-dihydro-15-keto-PGF2alpha, Gln Pro Lys, Dimercaprol, 6-Hydroxynicotinic acid, 3-dodecynoic acid, Leu Leu Ala, Asn Gln Gln, Ala Met Lys, |

| | | | | | | | |
|---|---|---|---|---|---|---|---|
| | | | | | | | Dihydrolevobunolol, 9-pentadecen-1-ol, VALERYL SALYCILATE, 2,5-Dimethoxycinnamic acid, Nalidixic acid, Pyridoxamine , ethylmalonic acid, Glu Ser, alpha,alpha-Trehalose, 3-Hydroxydodecanedioic acid, 5,7-nonadienoic acid , Pyroglutamic acid, D-Biotin, Ala Ala Asp, 1a,1b-dihomo-PGJ2, 5-Hydroxyindoleacetic acid , Embelin, DALBERGIONE, 4-METHOXY-4'-HYDROXY-, (R)-(+)-Citronellal, 3-Dehydroquinic acid, S-Succinyldihydrolipoamide, Gummiferol, Tetrahydrofuran, 9-lauroleic acid, EB 1213, Urocanic acid , 4-hydroxy enanthoic acid, 2-Hydroxy-3-(4-methoxyethylphenoxy)-propanoic acid, cyclohexylammonium, cis-3-(6-Hydroxy-7-methoxy-5-benzofuranyl)acrylic acid - 0.9427811, 5-(4-hydroxy-2,5-dimethylphenoxy)-2,2-dimethyl-Pentanoic acid (Gemfibrozil M1), Tyr Pro, 2H-1-Benzopyran-6-acetic acid, 7-hydroxy-8-methoxy-2-oxo-, Furafylline, PURPUROGALLIN, Lorazepam , alpha-ketoglutarate, Lidocaine, Tranexamic acid, phosphatidylethanolamine(20:4(5Z,8Z,11Z,14Z)/20:4(5Z,8Z,11Z,14Z))[U], DIHYDROFISSINOLIDE, Met His Lys , L-Urobilin, 2,4-DICHLOROPHENOXYBUTYRIC ACID, METHYL ESTER, myo-Inositol, 5beta-Androstan-3alpha-ol-17-one sulfate, phosphatidylethanolamine(18:1(11Z)/18:1(9Z))[U] + |

| | | | | | | | |
|---|---|---|---|---|---|---|---|
| | | | | | | | 2.416716, ARECOLINE, L-gamma-Cyano-gamma-aminobutyric acid, gamma-Hydroxyphenylbutazone glucuronide, Galbeta1-4GlcNAcbeta-Sp, 3alpha,12alpha,25-trihydroxy-5beta-cholestan-7-one, Pizotyline, DL-2-Aminooctanoic acid , thyrotropin releasing hormone, Purine - 5.6426544, epsilon-Caprolactam + 6.236251, (2-Chlorophenyl)diphenylmethane, Phe Ala Arg, Met Glu Cys, CARYLOPHYLLENE OXIDE, Glutaral (Glutaraldehyde), 3-oxo-4-pentenoic acid, N-Acetylputrescine, S-Methylpenicillamine, Creatine, Val Gly, Glutaconic acid, isoamyl nitrite, Uric acid, 5-Acetyl-4-methylthiazole, Doxapram, 3-Hydroxy-4-methoxyphenylacetic acid, methyl 8-[2-(2-formyl-vinyl)-3-hydroxy-5-oxo-cyclopentyl]-octanoate, GPSer(18:0/20:4(5Z,8Z,11Z,14Z)), THIODIGLYCOL, KOBUSONE, 9-amino-nonanoic acid, Uridine, Isoquinoline N-oxide, Cilastatin, 3,3,45-Tetrahydroxystilbene , phosphatidylethanolamineNMe(18:1(9Z)/18:1(9Z))[U], Mannitol, Indole-3-ethanol, 1-(11E-octadecenoyl)-rac-glycerol, Pergolide, 1-Hydroxy-2-naphthoic acid, 3-Methyl-2-oxovaleric acid, EMODIC ACID, Flavin adenine dinucleotide (FAD), PRISTIMEROL, 10,11-epoxy-chlorovulone I, 1-(2-Pyrimidyl)piperazine, AGELASINE, Gibberellin A9, Glucoheptonic acid, 4-hydroxy pelargonic acid, |

| | | | | | | | |
|---|---|---|---|---|---|---|---|
| | | | | | | | HYDROLYSIS PRODUCT OF BUSSEIN - 1.1705549, phosphatidylethanolamine(O-16:0/22:5(4Z,7Z,10Z,13Z,16Z)), 3-(a-Naphthoxy)lactic acid glucuronide, 9,12-dioxo-dodecanoic acid , 3beta-ACETOXYDEOXYANGOLENSIC ACID, METHYL ESTER, OXOLINIC ACID, Bilirubin , 3'-Methoxy-E,E-dienoestrol, Xanthine , Sedoheptulose, 2-Naphthaleneacetic acid, 6-hydroxy- - 2.938551, phosphatidylethanolamineNMe(18:2(9Z,12Z)/18:2(9Z,12Z))[U], 7-Hydroxyoctanoic acid, 3,7,12-Trioxochola-1,5-dien-24-oic Acid, 9R-hydroxy-10E-octadecenoic acid, D-Leucic acid, SULBACTAM , 9-chloro-10-hydroxy-hexadecanoic acid + 1.4075756, 4-Pyridoxic acid, Galabiosylceramide (d18:1/12:0), 10-hydroxy-2E,8E-Decadiene-4,6-diynoic acid, phosphatidylethanolamine(18:0/20:4(5Z,8Z,11Z,14Z))[U] - 5.053175, (+/-)-13-HDoHE, Diglycolic acid, Urea, Nalorphine, alpha-hydroxyisobutyrate, Norcodeine, Hypoxanthine, Dehydroascorbic acid, 2-Aminopropanol, threo-Isocitric acid, Propanoic acid, 2-hydroxy-3-[(4-hydroxy-1-naphthalenyl)oxy]-, Vigabatrin , Granisetron metabolite 1, L-gamma-Cyano-gamma-aminobutyric acid , N-gamma-Acetyl-N-2-formyl-5-methoxykynurenamine, 2-Methyl-3-ketovaleric acid , Phenol, Promazine, L-Glutamic acid dibutyl ester , beta-D-Glucopyranosiduronic |

| | | | | | | | |
|---|---|---|---|---|---|---|---|
| | | | | | | | acid, 2-(1H-indol-4-yloxy)-1-[[(1-methylethyl)amino]methyl]ethyl - 10.241287, Tyr Tyr Thr, Isoacitretin, L-DOPA, Glucosylceramide (d18:1/20:0), GPEtn(13:0/15:0)[U], D-Leucic acid , 4-Hydroxytacrine, HAEMATOXYLIN, 8,13-dihydroxy-9,11-octadecadienoic acid, N2,N2-Dimethylguanosine, Ala Phe Arg, Estrone sulfate, 5-[2-(hydroxymethyl)-5-methylphenoxy]-2,2-dimethyl-Pentanoic acid (Gemfibrozil M4), Cyclopenthiazide, 3-oxo-dodecanoic acid, dihydroxyacetone phosphate (DHAP), 3-Amino-2-piperidone, 1-heptadecanoyl-2-(9Z-heptadecenoyl)-3-eicosanoyl-sn-glycerol, N1-Acetylspermidine, Pyrrole-2-carboxylic acid, Catalpol, MERBROMIN, Imidazolone , Pro Lys Pro, Creatinine , 9(S)-HODE-d4, Thr Gly, Asp Glu, BENZANTHRONE , Lactate, beta-Alanine , Benzaldehyde, p-amino-, thiosemicarbazone, 4-Guanidinobutanoate, 4-Aminophenyl 1-thio-beta-D-glucuronide + 2.5971124, cysteine, beta-vinyl acrylic acid, Clonidine Metabolite 3, N1,N4-Diacetylsulfanilamide, Cys Cys Tyr, 1-(7Z,10Z,13Z,16Z-docosatetraenoyl)-2-(7Z,10Z,13Z,16Z,19Z-docosapentaenoyl)-sn-glycerol, Allantoin , Octane, Erythrono-1,4-lactone, 3-(Imidazol-4-yl)-2-oxopropyl phosphate, Alfentanil, 3-Hydroxyphenytoin, 1-Hexadecylamine, Arg Cys Cys, Oxazepam, 2-Naphthaleneacetic acid, 6-hydroxy, 4-Hydroxypyridine, |

| | | | | | | | |
|---|---|---|---|---|---|---|---|
| | | | | | | | O-BENZYL-l-SERINE, (22E,24E,26E)-1alpha,26b-dihydroxy-22,23,24,25,26,26a-hexadehydro-26a,26b-dihomo-27-norvitamin D3 /, B-ureidoisobutyric acid, ANISOMYCIN, N-Acetyl-p-benzoquinonimine, 7E-decen-1-ol, Dihydrokaempferol, Hydromorphone, PGE2alpha dimethyl amine, 2-Octenedioic acid, Anthracene, 3E-undecenoic acid, 5-tert-Butyl-4-hydroxymethylfuran-2-carboxylic acid, Gly His, Carbaryl, 5-Methoxyindoleacetate , BUFEXAMAC, Arg Asn Gln, (22R)-1alpha,22,25-trihydroxy-26,27-dimethyl-23,24-tetradehydro-24a-homo-20-epivitamin D3 / (22R)-1a, 3-Methylindole, Chloramphenicol alcohol, 13E,17-octadecadienoic acid , 1alpha,25-dihydroxy-2beta-(5-hydroxypentoxy)vitamin D3 / 1alpha,25-dihydroxy-2beta-(5-hydroxypentoxy, clavulone I, Urea , SULBACTAM, Phenyl sulfate, Asn Gly Ser, Succinic anhydride , DICHLORODIPHENYLTRICHLOROETHANE, Methylphenidate, Tyrosine , Caffeine, Phenytoin-N-glucuronide<br>TCA cycle, Cholesterol and sphingolipids transport, Vitamin d2 metabolism, Polyamine metabolism, Urea cycle, Intracellular cholesterol transport, (L)-Arginine metabolism, cortisone biosynthesis and metabolism, lipid metabolism, FXR-regulated cholesterol and bile acid transport, Lysine |

| | | | | | | | |
|---|---|---|---|---|---|---|---|
| | | | | | | | metabolism, Bile acid metabolism, Regulation of CFTR gating, Role of VDR in regulation of genes involved in osteoporosis, Vitamin D3 metabolic C-23 and C-24 pathways, Triacylglycerol biosynthesis in obesity and diabetes mellitus, type II, Mechanism of action of DGaT1 in obesity and diabetes mellitus, type II, Aminoacyl-tRNA biosynthesis in mitochondria , Triacylglycerol metabolism p.2, Muscle contraction_nNOS signaling in skeletal muscles, Aminoacyl-tRNA biosynthesis in cytoplasm , tryptophan metabolism, Regulation of lipid MB FXR-dependent negative-feedback regulation of bile acid concentration, Cortisol biosynthesis from cholesterol , Development_Activation of astroglia cell proliferation by ACM3, Fatty Acid Omega Oxidation , Prostaglandin 2 biosynthesis and metabolism, Aspartate and aspargine metabolism, Nicotine metabolism in liver, Cholesterol and sphingolipids transport/transport from Golgi, Glycolysis and gluconeogenesis, FXR-regulated cholesterol and bile acid cellular transport, Saturated fatty acids metabolism, Cortisol BS from cholesterol, saturated fatty acid metabolism, aminoacyl-tRNA biosynthesis in cytoplasm, neurophysiological process_, melatonin signaling, Nicotine MB in liver, Role of Diethylhexyl Phthalate and Tributyltin in fat differentiation, Nitrogen metabolism, Cholesterol metabolism, Arginine metabolism, Bile Acid Biosynthesis, Aminoacyl-tRNA biosynthesis in mitochondrion, |

| | | | | | | | |
|---|---|---|---|---|---|---|---|
| | | | | | | | Histidine-glutamate-glutamine metabolism, Neurophysiological process_GABAergic neurotransmission, tryptophan metabolism , Proline metabolism, Saturated fatty acid biosynthesis, Catecholamine metabolism, Muscle contraction_nNOS Signaling in Skeletal Muscle, Acetylcholine biosynthesis and metabolism, Development Activation of astroglial cells proliferation by ACM3, Fatty Acid Omega Oxidation, Nicotine signaling in GABAergic neurons, Neurophysiological process_ ACM1 and ACM2 in neuronal…, Serotonin modulation of dopamine release in nicotine addiction, Transport ACM3 in salivary glands, Development_ACM2 and ACM4 activation of ERK, Regulation of CFTR gating (nomal and CF), HETE and HPETE biosynthesis and metabolism, Neurophysiological process_Role of CDK5 in presynaptic signaling, Role of Diethylhexyl Phthalate and Tributyltin in fat cell differentiation, Phosphatidylinositol metabolism, Aldosterone biosynthesis and metabolism, Transcription role of VDR in regulation of genes involved in osteoporosis, Tyrosine metabolism p.1(dopamine) |
| Kaddurah-Daouk[30]2013 | Case-control study | 40 AD<br>36 MCI<br>38 CN | Yes | Penn Memory Center, University of Pennsylvania (Philadelphia,PA, USA) and the Maria de los Santos Health Center (Philadelphia, PA, USA), | CSF | liquid chromatography electrochemical array platform | MET, 5-HIAA, Xanthosine, GSH, VMA, Hypoxanthine, Indole-3-acetic acid, Uric acid, 5-HTP, Kynurenine, TRP |

| | | | | | | | |
|---|---|---|---|---|---|---|---|
| Sato[31]2012 | Case-control study | Test set:<br>10 AD<br>10 CN<br>validation set:<br>41 AD<br>26 MCI<br>42 CN | Yes | PrecisionMed, Inc. (San Diego, CA) | plasma and CSF | LC/APCI-MS;<br>GC-MS | Desmosterol |
| Czech[32]2012 | Case-control study | 53 Light to mild AD (MMSE>22)<br>26 Moderate to Strong AD (MMSE 14-22)<br>51 CN | Yes | five different clinical centres in Europe: One in Germany, one in France, one in Switzerland, and two in Sweden | CSF | GC-MS;<br>LC-MS/MS | 5-Hydroxy-3-indoleacetic acid , Noradrenaline, 3,4-Dihydroxyphenylalanine, 4-Hydroxy-3-methoxy-phenylglycol, Histamine, 21-Hydroxyprogesterone, Cortisol, Normetanephrine, Citrulline, Cysteine, Methionine, Ornithine (incl. Arginine, Citrulline), Arabinose, Erythrol, Galactitol, Lyxose, myo-Inositol, Ribonic acid, Sorbitol (incl. Mannitol, Galactitol), scyllo-Inositol, Pyruvate (incl. Phosphoenolpyruvate), Carnitine, Pseudouridine, putative Tartronic acid, Quinic acid (incl. Chlorogenic acid), Salicylic acid, Uric acid, Choline, uridine, 3-methoxy-4-hydroxy phenylglycol (MHPG), dopamine, noradrenaline , normetanephrine., sorbitol, tyrosine, phenylalanine, serine, pyruvate, taurine, creatinine, dopamine , ornithine |
| Ibanez[33]2013 | Nested case-control study | 21 AD<br>21 MCI-S<br>12 MCI-AD<br>21 CN | No | unknown | CSF | RP/UHPLC-TOF MS;<br>HILIC/UHPLC-TOF MS | uracil, xanthine, uridine, tyrosyl-serine (or serinyl-tyrosine), methyl-salsolinol, nonanoylglycine, dopamine-quinone, caproic acid, vanylglycol, histidine, pipecolic acid, hydroxyphophinyl-piruvate, creatinine, taurine, C16-sphingosine-1-phosphate, tryptophan, methylthioadenosine |

| | | | | | | | |
|---|---|---|---|---|---|---|---|
| Wang[34]2012 | Case-control study | 46 AD<br>39 CN | No | Centre of Harbin Elderly Care Service | serum | GC-MS | linolenic acid, cis-4,7,10,13,16,19-docosahexaenoic acid, myristic acid, palmitic acid, oleic acid |
| Oresic [35]2011 | Nested case-control study | 37 AD<br>52 Progressive MCI<br>91 Stable MCI<br>46 CN | No | longitudinal study databases gathered in the University of Kuopio | serum | UPLC-MS ;<br>GC GC-TOFMS | PC(16:0/18:2), PI(18:0/20:4), PC(O-18:0/18:2), PC(18:0/20:4), SM(d18:1/24:0), 2-ketobutyric acid, Sitosterol, Ketovaline, Histamine, lactic acid, PC(18:0/18:2), PC(16:0/20:4), PC (16:0/16:0), 2,4-dihydroxybutanoic acid, lysoPC (16:0), PC(16:0/20:5), PC (18:0/20:4) , PC (O-18:1/16:0), ribose-5-phosphate, pyruvic acid<br>pentose phosphate pathway |
| Kaddurah-Daouk[36]2011 | Case-control study | 15 AD<br>15 CN | Yes | autopsy program of the Joseph and Kathleen Bryan Alzheimer Disease Research Center (Bryan ADRC) | CSF | LC/ECA | tryptophan, indoleacetic acid, Norepinephrine |
| Han[37]2011 | Case-control study | 26 AD<br>26 CN | Yes | Joseph and Kathleen Bryan Alzheimer's Disease Research Center (Bryan ADRC) and the Department of Psychiatry both at Duke University | plasma | MDMS-SL | N22:1, N20:0, N22:0, N23:0, N23:1, N24:1, N21:0, N24:0, N17:1, N24:2, N18:0, N21:0, N16:0, N26:0, OH-N24:2, OH-N24:1, N28:2, N23:0 |
| Li[38]2010 | Case-control study | 20 AD<br>20 CN | No | Shengjing Affiliated Hospital<br>China Medical University. | plasma | UPLC-MS | Dihydrosphingosine, Tryptophan fragment, LPC C18:2, LPC C18:1, LPC C16:0, LPC C18:0, LPC C20:4, phytosphingosine, hexadecasphinganine |
| Lee[39]2007 | Case-control study | 36 AD<br>34 CN | No | patients:Gongju Geriatric Hospital. | urine | LC/ESI-MS | Pseudouridine, 1-Methyladenosine, 8-Hydroxy-2′-deoxyguanosine, 3-Methyluridine, N2, |

| | | | | | | | |
|---|---|---|---|---|---|---|---|
| | | | | | | | N2-dimethylguanosine, 2-Deoxyguanosine |
| Paik[40]2006 | Case-control study | 4 AD<br>22 CN | No | unknown | urine | GC-SIM-MS | N1-acetylputrescine, N1-acetylcadaverine, Putrescine, Cadaverine, N1-acetylspermidine, N8-acetylspermidine, Spermidine, N1-acetylspermine, Spermine |
| Yoshida[41]2009 | Case-control study | 39 AD<br>25 VD<br>24 CN | No | Aoisoranosato Geriatric Health Services Facility and the Arimakogen Hospital | plasma | GC-MS | t8-iso-PGF2, Ascorbic acid, Ubiquinol-10 , α-Tocopherol |
| Kim[42]2004 | Case-control study | 34 AD<br>20 CN | Yes | Gongju Geriatric Hospital | urine | GC-MS | PGF2α, PGF2ß |
| Fonteh[43]2013 | Case-control study | 29<br>40 MCI<br>79 CN | Yes | The local Institutional Review Board (Huntington Hospital Pasadena CA) | CSF | LC-MS/MS | PC(32a:0), PC(34p:0/34e:1), PC(34a:1), PC(34a:0), PC(36a:1), PC(38a:5), PC(36a:0/38p:6), PC(38a:6), 1-radyl-2-acyl- sn -glycerophosphoethanolamine, 1,2-diacyl- sn -glycerophosphoserine , PE(40p:4/40e:5), PS(34a:3), PS(36a:4) |
| Wang[44]2005 | Case-control study | 8 AD<br>8 CN | Yes | University of Kentucky Alzheimer's Disease Research Center Clinic; All controls were followed longitudinally in the control clinic | nuclear and mitochondrial DNA of frontal, parietal, and temporal lobes and cerebellum from short | GC/MS-SIM | 8-hydroxyadenine, 5-hydroxycytosine, fapyadenine, 5-hydroxyuraci, Fapyguanine, 8-hydroxyguanine |

| | | | | | | | |
|---|---|---|---|---|---|---|---|
| | | | | | postmortem interval AD brain | | |
| Huang[45]2004 | Case-control study | 3 AD<br>3 CN | Yes | unknown | brain tissue | NMR; GC, GC-MS | glucose |
| Gonzalez-Dominguez [46]2015 | Case-control study | 8 AD<br>21 CN | Yes | Neurological Service of Hospital Juan Ramón Jiménez | serum | GC-MS | Valine, Urea, Aspartic acid, Pyroglutamic acid, Glutamine, Phenylalanine, Asparagine, Ornithine, Pipecolic acid, Histidine, Tyrosine, Palmitic acid, Uric acid, Tryptophan, Stearic acid, Cystine, Lactic acid, α-Ketoglutarate, Isocitric acid, Glucose, Adenosine, Cholesterol, Oleic acid<br>Alanine, aspartate and glutamate metabolism, TCA cycle, tryptophan metabolism, arginine and proline metabolism, histidine metabolism, pyruvatemetabolism, phenylalanine metabolism |
| Whiley[47]2014 | Case-control study | screen phase:<br>10 AD<br>10 MCI<br>10 CN<br>Validation phase：<br>Phosphatidylcholine analysis：<br>42 AD<br>50 MCI<br>49 control<br>Total plasma fatty acid analysis： | Yes | the AddNeuroMed cohort and the King's College London Dementia Case Register (DCR) | plasma | NMR;<br>LC-MS | PC 16:0/20:5, PC16:0/22:6, PC18:0/22:6, eicosapentaenoic acid |

| | | | | | | | |
|---|---|---|---|---|---|---|---|
| | | 10 AD<br>10 MCI<br>10 control<br>total choline：<br>n=35 | | | | | |
| Popp[48]2013 | Case-control study | 106 AD<br>87 CN | No | Memory Clinic at the Department of Psychiatry University of Bonn | Plasma and CSF | GC-MS and gas chromatography-flame ionization detection | Cholesterol, 24s-hydroxycholesterol, 27-hydroxycholesterol, lanosterol, lathosterol |
| Snowden[49]2017 | Case-control study | 14 AD<br>15 ASYMAD<br>14 CN | No | BLSA | brain tissue | LC-MS;<br>GC-MS | Cholesterol, Linoleic Acid, Cholestenol, Docosahexaenoic acid, Carbamic acid, Methylheptadecadiynoic acid, Oleic acid, Palmitic acid, Hexanedioic acid, Dimethylglycine, Guanidobutanoate, Ascorbate, Aminobutanal, Gluconic acid, Cysteine, Aspartate, L-DOPA, Fumaric acid, Linolenic acid, Indoleacetic acid, Eicosapentaenoic acid, Allantoin, Hypoxanthine, Coumaric acid, Adenine, Oxoarginine, Deoxyflurouridine, Arginine, GABA, Methylstearate, Octadecanal, eicosapentaenoic acid , arachidonic acid, docosahexaenoic acid |
| Guiraud[50]2017 | Case-control study | 6 AD<br>6 CN | No | PrecisionMed Inc. | plasma and CSF | UPLC-MS/MS | S-adenosylhomocysteine, S-adenosylmethionine, glycine |
| Graham[51]2015 | Nested case-control study | 19 MCI-AD<br>16 MCI<br>37 CN | Yes | patients: Belfast City Hospital memory clinic; Controls were recruited from groups of volunteers that have previously | plasma | HRMS | 4-aminobutanal, Creatine, GABA, L-arginine, L-ornithine, Methylthioadenosine, N1 or N8-acetyl-spermidine, N1,N12-diacetlyspermine, N-acetylputrescine, Putrescine, Spermidine, Spermine<br>Lysine metabolism, Tryptophan metabolism, Polyamine |

| | | | | | | | |
|---|---|---|---|---|---|---|---|
| | | | | assisted with studies of this type or were spouses of patients | | | metabolism, Urea cycle, Phospholipid metabolism p.1, CHOL & Sphingolipid transport, Sat fatty acid biosynthesis, Regulation of lipid metabolism, Glycolysis & GNG p.2, Transport of IC CHO, Niacin-HDL metabolism, Plasmalogen biosynthesis, UMP biosynthesis, GABA biosynthesis& metabolism, PG 2 biosynthesis & metabolism, Glycolysis GNG(short map), L-Arginine metabolism, CHOL sphingolipid transport, Transport of IC CHOL, Glycolysis & GNG p.1, Prostaglandin 2 biosynthesis and metabolism, Ascorbate metabolism, Glycolysis &GNG p.2, Tyrosine metabolism p.1, Catecholamine metabolism, NAE & PL A2 pathway |
| Kim[52]2017 | Case-control study | 205 AD<br>207 CN | No | patients：Dementia Case Register (DCR) at King's College London and the EU funded AddNeuroMed study；Normal elderly control subjects: non-related family members of AD patients care-givers' relatives social centers for the elderly or GP surgeries | plasma | UPLC-MS | PC36:5, PC38：6, Cer24:1, Cer16:0, Cer18:0, PC40:6 |
| Qun Liang[53]2015 | Case-control study | 256 AD<br>218 CN | Yes | First Affiliated Hospital Heilongjiang University of Chinese Medicine. | saliva samples | FUPLC-MS | Sphinganine-1-phosphate, Ornithine, Phenyllactic acid, Inosine, 3-Dehydrocarnitine, Hypoxanthine |
| Andrea | Case-control | 30 AD | Yes | MCI/AD Italian prevention | plasma | LC-MS/MS | d18:1/16:0, d18:1/24:0, d18:1/24:1, d18:1/16:0, d18:1/18:0, |

| | | | | | | | |
|---|---|---|---|---|---|---|---|
| Armirotti[54]2014 | study | 30 MCI<br>30 CN | | project | | | d18:1/24:1, d18:1/24:1, d18:1/16:0, d18:1/16:0, d18:1/24:1 |
| Iltaf Shah[55]2012 | Case-control study | 26 untreated AD<br>44 trrated AD<br>35 CN | No | memory clinic in East Sussex UK | serum | LC-MS/MS | 25OHD2, Vitamin D2, Vitamin D3 |
| Yu Cui[56]2014 | Case-control study | 46 AD<br>47 CN | No | Centre of Harbin Elderly Care Service Heilongjiang Province in the North of China | serum | UPLC-QTOF-MS | PGE2α dimethyl amine, Monoiodothyronine, Palmitic amide, (6R)-vitamin D3 6,19- (4-phenyl-1,2,4-triazoline-3,5-dione), Trimethyltridecanoic acid, 1-Methylinosine, 16-bromo-9-hexadecenoic acid, PS(18:1/0:0), LysoPC(18:0), LysoPC(20:3), LysoPC(18:2), Dihydrosphingosine, N-Acetylglutamine, 1α,25-dihydroxy-2α-(3-hydroxypropoxy) vitamin D3, N-Acryloylglycine, Argininosuccinic acid, Dethiobiotin, Isobutyryl-L-carnitine, 2-hydroxy-N-(2-hydroxyethyl)-N,N-dimethyl-1-dodecanaminium,, L-aspartyl-4-phosphate, L-glutamine, 5-L-glutamylglycine, azelaic acid, aminopterin, cytidine, acetyl-L-carnitine , stra-1,3,5(10),7-tetraene-3,17α-diol, p-cresol glucuronide, etiocholanolone glucuronide |
| Muguruma [57] 2018 | Case-control study | 10 AD<br>10 CN | No | unknown | CSF | UHPLC-MS/MS | anthranilic acid, methionine sulfoxide, N1N8-diacetyl-spermidine, Trp , kynurenine, ornithine, 3-hydroxykyurenine, N1N12-diacetyl-spermine, anthranilate, Phe, diacetyl-spermidine, diacetyl-spermine, |
| van der Lee [58]2018 | Cohort study | 1356 AD,<br>23882 CN | No | ERF, RS,WHII, VUmc ADC, AgeCoDe, EGCUT , FHS, Finrisk97, and DILGOM | plasma | NMR | S-HDL-free cholesterol, M-HDL-phospholipids, DHA, Glutamine |

| | | | | | | | |
|---|---|---|---|---|---|---|---|
| Tukiainen [59]2008 | Cohort study | unknown | No | the city of Kuopio | serum | 1H NMR | valine, lactate, 3-hydroxybutyrate, acetate, glucose |
| Ellison [60]2017 | Case-control study | 10 CN<br>8 PCAD<br>11 MCI<br>10 LOAD | Yes | autopsies from the Neuropathology Core of the University of Kentucky Alzheimer's Disease Center (UK-ADC) | DNA of brain tissue | GC-MS | cytosine, 5-methylcytosine, 5-hydroxmethylcytosine |
| Dayon [61]2017 | Case-control study | Cognitively normal (CDR 0) :n = 48<br>Cognitively impaired(CDR 0.5 or 1): n = 72 | Yes | MCI:<br>the memory clinics, departments of psychiatry, and the Leenaards Memory Center, Department of Clinical Neurosciences, University Hospitals of Lausanne (Switzerland). CN: the community through advertisement or among the spouses of memory clinic patients. | CSF and plasma | LC-MS / MS | cystathionine, methionine, SAH, SAM, serine, cysteine, 5-MTHF, cystathionine, glycine, methionine, SAH, SAM, serine, cysteine, Hcy |
| Fiandaca [62]2015 | Cohort study | discovery cohort:<br>53 normal<br>18 phenoconverters<br>internal validation:<br>20 normal<br>10 phenoconverters | Yes | participation at the University of Rochester and the University of California Irvine | plasma | MRM-SID-MS | PC ae C40:6, PC aa C40:1, PC aa C38:6, PC aa C38:0, PC aa C36:6, lysoPC a C18:2, C3, PC ae C36:4, C10:2, C9, PC ae C42:1, PC aa C38:3, C5, ADMA, Asn, PC aa C34:4, C18:1-OH, PC ae C34:0, C5-OH (C3-DC-M), PC aa C40:5, PC aa C32:0, C16:2, C12:1, C10:1 |

| | | | | | | | |
|---|---|---|---|---|---|---|---|
| Ibanez [63]2012 | Nested case control study | training set:<br>19 CN<br>23 MCI-nonAD<br>9 MCI-AD<br>23 AD<br>test set:<br>4 CN<br>2 MCI-nonAD<br>4 MCI-AD<br>2 AD | No | Karolinska University Hospital | CSF | CE-MS | choline, valine, arginine, tripeptide, suberylglycine, carnitine, dimethy-L-arginine, creatine, serine, hisidine |
| Ronnemaa [64]2012 | Cohort study | baseline:<br>2009 participants<br>During a 35 year follow-up time:<br>213 dementia<br>91 AD<br>1796 control. | No | ULSAM cohort | serum | GC | Myristic acid, Palmitic acid, Stearic acid, Oleic acid, Linoleic acid |
| Toledo [65]2017 | Cohort study | 175 AD<br>356 MCI<br>199 CN | No | ADNI-1 cohort | serum | UPLC-MS/MS | PC ae C40:3, PC ae C42:4, PC ae C44:4, SM (OH) C14:1, SM C16:0, SM C20:2, PC ae C36:2 |
| Takayama [66]2015 | Case-control | unknown | No | Fukushimura Brain Bank | brain tissue | LC-ESI-MS/MS | L-phenylalanine, L-lactic acid |
| Shah [67]2014 | Case-control | 12AD<br>32CN | No | a memory clinic in East Sussex | serum | LC-MS/MS | 24R,25(OH)2D3, 1alpha-25-(OH)2D2 |

Reference:

1. Varma VR, Oommen AM, Varma S, Casanova R, An Y, Andrews RM, et al. Brain and blood metabolite signatures of pathology and progression in Alzheimer disease: A targeted metabolomics study. *PLoS medicine* (2018) 15(1):e1002482. Epub 2018/01/26. doi: 10.1371/journal.pmed.1002482. PubMed PMID: 29370177; PubMed Central PMCID: PMCPMC5784884.
2. Marksteiner J, Blasko I, Kemmler G, Koal T, Humpel C. Bile acid quantification of 20 plasma metabolites identifies lithocholic acid as a putative biomarker in Alzheimer's disease. *Metabolomics : Official journal of the Metabolomic Society* (2018) 14(1):1. Epub 2017/12/19. doi: 10.1007/s11306-017-1297-5. PubMed PMID: 29249916; PubMed Central PMCID: PMCPMC5725507.
3. Pan X, Elliott CT, McGuinness B, Passmore P, Kehoe PG, Holscher C, et al. Metabolomic Profiling of Bile Acids in Clinical and Experimental Samples of Alzheimer's Disease. *Metabolites* (2017) 7(2). Epub 2017/06/21. doi: 10.3390/metabo7020028. PubMed PMID: 28629125; PubMed Central PMCID: PMCPMC5487999.
4. Oresic M, Anderson G, Mattila I, Manoucheri M, Soininen H, Hyotylainen T, et al. Targeted Serum Metabolite Profiling Identifies Metabolic Signatures in Patients with Alzheimer's Disease, Normal Pressure Hydrocephalus and Brain Tumor. *Frontiers in neuroscience* (2017) 11:747. Epub 2018/01/30. doi: 10.3389/fnins.2017.00747. PubMed PMID: 29375291; PubMed Central PMCID: PMCPMC5767271.
5. Lau HC, Yu JB, Lee HW, Huh JS, Lim JO. Investigation of Exhaled Breath Samples from Patients with Alzheimer's Disease Using Gas Chromatography-Mass Spectrometry and an Exhaled Breath Sensor System. *Sensors (Basel, Switzerland)* (2017) 17(8). Epub 2017/08/05. doi: 10.3390/s17081783. PubMed PMID: 28771180; PubMed Central PMCID: PMCPMC5579482.
6. Proitsi P, Kim M, Whiley L, Pritchard M, Leung R, Soininen H, et al. Plasma lipidomics analysis finds long chain cholesteryl esters to be associated with Alzheimer's disease. *Translational psychiatry* (2015) 5:e494. Epub 2015/01/15. doi: 10.1038/tp.2014.127. PubMed PMID: 25585166; PubMed Central PMCID: PMCPMC4312824.
7. Oberacher H, Arnhard K, Linhart C, Diwo A, Marksteiner J, Humpel C. Targeted Metabolomic Analysis of Soluble Lysates from Platelets of Patients with Mild Cognitive Impairment and Alzheimer's Disease Compared to Healthy Controls: Is PC aeC40:4 a Promising Diagnostic Tool? *Journal of Alzheimer's disease : JAD* (2017) 57(2):493-504. Epub 2017/03/09. doi: 10.3233/jad-160172. PubMed PMID: 28269764.
8. de Leeuw FA, Peeters CFW, Kester MI, Harms AC, Struys EA, Hankemeier T, et al. Blood-based metabolic signatures in Alzheimer's disease. *Alzheimer's & dementia (Amsterdam, Netherlands)* (2017) 8:196-207. Epub 2017/09/28. doi: 10.1016/j.dadm.2017.07.006. PubMed PMID: 28951883; PubMed Central PMCID: PMCPMC5607205.
9. Zhu M, Wang X, Hjorth E, Colas RA, Schroeder L, Granholm AC, et al. Pro-Resolving Lipid Mediators Improve Neuronal Survival and Increase Abeta42 Phagocytosis. *Molecular neurobiology* (2016) 53(4):2733-49. Epub 2015/12/10. doi: 10.1007/s12035-015-9544-0. PubMed PMID: 26650044; PubMed Central PMCID: PMCPMC4824659.
10. Xu J, Begley P, Church SJ, Patassini S, Hollywood KA, Jullig M, et al. Graded perturbations of metabolism in multiple regions of human brain in Alzheimer's disease: Snapshot of a pervasive metabolic disorder. *Biochimica et biophysica acta* (2016) 1862(6):1084-92. Epub 2016/03/10.

doi: 10.1016/j.bbadis.2016.03.001. PubMed PMID: 26957286; PubMed Central PMCID: PMCPMC4856736.

11. Paglia G, Stocchero M, Cacciatore S, Lai S, Angel P, Alam MT, et al. Unbiased Metabolomic Investigation of Alzheimer's Disease Brain Points to Dysregulation of Mitochondrial Aspartate Metabolism. *Journal of proteome research* (2016) 15(2):608-18. Epub 2015/12/31. doi: 10.1021/acs.jproteome.5b01020. PubMed PMID: 26717242; PubMed Central PMCID: PMCPMC5751881.

12. Nasaruddin ML, Holscher C, Kehoe P, Graham SF, Green BD. Wide-ranging alterations in the brain fatty acid complement of subjects with late Alzheimer's disease as detected by GC-MS. *American journal of translational research* (2016) 8(1):154-65. Epub 2016/04/14. PubMed PMID: 27069549; PubMed Central PMCID: PMCPMC4759425.

13. Casanova R, Varma S, Simpson B, Kim M, An Y, Saldana S, et al. Blood metabolite markers of preclinical Alzheimer's disease in two longitudinally followed cohorts of older individuals. *Alzheimer's & dementia : the journal of the Alzheimer's Association* (2016) 12(7):815-22. Epub 2016/01/26. doi: 10.1016/j.jalz.2015.12.008. PubMed PMID: 26806385; PubMed Central PMCID: PMCPMC4947451.

14. Gonzalez-Dominguez R, Ruperez FJ, Garcia-Barrera T, Barbas C, Gomez-Ariza JL. Metabolomic-Driven Elucidation of Serum Disturbances Associated with Alzheimer's Disease and Mild Cognitive Impairment. *Current Alzheimer research* (2016) 13(6):641-53. Epub 2016/01/31. PubMed PMID: 26825096.

15. Ciavardelli D, Piras F, Consalvo A, Rossi C, Zucchelli M, Di Ilio C, et al. Medium-chain plasma acylcarnitines, ketone levels, cognition, and gray matter volumes in healthy elderly, mildly cognitively impaired, or Alzheimer's disease subjects. *Neurobiology of aging* (2016) 43:1-12. Epub 2016/06/04. doi: 10.1016/j.neurobiolaging.2016.03.005. PubMed PMID: 27255810.

16. Yoshida M, Higashi K, Kuni K, Mizoi M, Saiki R, Nakamura M, et al. Distinguishing mild cognitive impairment from Alzheimer's disease with acrolein metabolites and creatinine in urine. *Clinica chimica acta; international journal of clinical chemistry* (2015) 441:115-21. Epub 2014/12/30. doi: 10.1016/j.cca.2014.12.023. PubMed PMID: 25542982.

17. Vankova M, Hill M, Velikova M, Vcelak J, Vacinova G, Dvorakova K, et al. Preliminary evidence of altered steroidogenesis in women with Alzheimer's disease: Have the patients "OLDER" adrenal zona reticularis? *The Journal of steroid biochemistry and molecular biology* (2016) 158:157-77. Epub 2015/12/26. doi: 10.1016/j.jsbmb.2015.12.011. PubMed PMID: 26704533.

18. Cho SH, Jung BH, Lee WY, Chung BC. Rapid column-switching liquid chromatography/mass spectrometric assay for DHEA-sulfate in the plasma of patients with Alzheimer's disease. *Biomedical chromatography : BMC* (2006) 20(10):1093-7. Epub 2006/04/04. doi: 10.1002/bmc.647. PubMed PMID: 16583455.

19. Koal T, Klavins K, Seppi D, Kemmler G, Humpel C. Sphingomyelin SM(d18:1/18:0) is significantly enhanced in cerebrospinal fluid samples dichotomized by pathological amyloid-beta42, tau, and phospho-tau-181 levels. *Journal of Alzheimer's disease : JAD* (2015) 44(4):1193-201. Epub 2014/11/20. doi: 10.3233/jad-142319. PubMed PMID: 25408209; PubMed Central PMCID: PMCPMC4699259.

20. Klavins K, Koal T, Dallmann G, Marksteiner J, Kemmler G, Humpel C. The ratio of

phosphatidylcholines to lysophosphatidylcholines in plasma differentiates healthy controls from patients with Alzheimer's disease and mild cognitive impairment. *Alzheimer's & dementia (Amsterdam, Netherlands)* (2015) 1(3):295-302. Epub 2016/01/09. doi: 10.1016/j.dadm.2015.05.003. PubMed PMID: 26744734; PubMed Central PMCID: PMCPMC4700585.
21. Gonzalez-Dominguez R, Garcia-Barrera T, Gomez-Ariza JL. Application of a novel metabolomic approach based on atmospheric pressure photoionization mass spectrometry using flow injection analysis for the study of Alzheimer's disease. *Talanta* (2015) 131:480-9. Epub 2014/10/05. doi: 10.1016/j.talanta.2014.07.075. PubMed PMID: 25281130.
22. Cui Y, Chen X, Liu L, Xie W, Wu Y, Wu Q, et al. Gas chromatography-mass spectrometry analysis of the free fatty acids in serum obtained from patients with Alzheimer's disease. *Bio-medical materials and engineering* (2015) 26 Suppl 1:S2165-77. Epub 2015/09/26. doi: 10.3233/bme-151522. PubMed PMID: 26405996.
23. Ansoleaga B, Jove M, Schluter A, Garcia-Esparcia P, Moreno J, Pujol A, et al. Deregulation of purine metabolism in Alzheimer's disease. *Neurobiology of aging* (2015) 36(1):68-80. Epub 2014/10/15. doi: 10.1016/j.neurobiolaging.2014.08.004. PubMed PMID: 25311278.
24. Wisniewski T, Newman K, Javitt NB. Alzheimer's disease: brain desmosterol levels. *Journal of Alzheimer's disease : JAD* (2013) 33(3):881-8. Epub 2012/10/09. doi: 10.3233/jad-2012-121453. PubMed PMID: 23042211; PubMed Central PMCID: PMCPMC3557460.
25. Gonzalez-Dominguez R, Garcia-Barrera T, Gomez-Ariza JL. Combination of metabolomic and phospholipid-profiling approaches for the study of Alzheimer's disease. *Journal of proteomics* (2014) 104:37-47. Epub 2014/01/30. doi: 10.1016/j.jprot.2014.01.014. PubMed PMID: 24473279.
26. Gonzalez-Dominguez R, Garcia A, Garcia-Barrera T, Barbas C, Gomez-Ariza JL. Metabolomic profiling of serum in the progression of Alzheimer's disease by capillary electrophoresis-mass spectrometry. *Electrophoresis* (2014) 35(23):3321-30. Epub 2014/08/20. doi: 10.1002/elps.201400196. PubMed PMID: 25136972.
27. Liu Y, Li N, Zhou L, Li Q, Li W. Plasma metabolic profiling of mild cognitive impairment and Alzheimer's disease using liquid chromatography/mass spectrometry. *Central nervous system agents in medicinal chemistry* (2014) 14(2):113-20. Epub 2014/12/18. PubMed PMID: 25515716.
28. Gonzalez-Dominguez R, Garcia-Barrera T, Gomez-Ariza JL. Using direct infusion mass spectrometry for serum metabolomics in Alzheimer's disease. *Analytical and bioanalytical chemistry* (2014) 406(28):7137-48. Epub 2014/09/19. doi: 10.1007/s00216-014-8102-3. PubMed PMID: 25230597.
29. Trushina E, Dutta T, Persson XM, Mielke MM, Petersen RC. Identification of altered metabolic pathways in plasma and CSF in mild cognitive impairment and Alzheimer's disease using metabolomics. *PloS one* (2013) 8(5):e63644. Epub 2013/05/24. doi: 10.1371/journal.pone.0063644. PubMed PMID: 23700429; PubMed Central PMCID: PMCPMC3658985.
30. Kaddurah-Daouk R, Zhu H, Sharma S, Bogdanov M, Rozen SG, Matson W, et al. Alterations in metabolic pathways and networks in Alzheimer's disease. *Translational psychiatry* (2013) 3:e244. Epub 2013/04/11. doi: 10.1038/tp.2013.18. PubMed PMID: 23571809; PubMed Central PMCID: PMCPMC3641405.

31. Sato Y, Suzuki I, Nakamura T, Bernier F, Aoshima K, Oda Y. Identification of a new plasma biomarker of Alzheimer's disease using metabolomics technology. *Journal of lipid research* (2012) 53(3):567-76. Epub 2011/12/29. doi: 10.1194/jlr.M022376. PubMed PMID: 22203775; PubMed Central PMCID: PMCPMC3276481.
32. Czech C, Berndt P, Busch K, Schmitz O, Wiemer J, Most V, et al. Metabolite profiling of Alzheimer's disease cerebrospinal fluid. *PloS one* (2012) 7(2):e31501. Epub 2012/02/24. doi: 10.1371/journal.pone.0031501. PubMed PMID: 22359596; PubMed Central PMCID: PMCPMC3281064.
33. Ibanez C, Simo C, Barupal DK, Fiehn O, Kivipelto M, Cedazo-Minguez A, et al. A new metabolomic workflow for early detection of Alzheimer's disease. *Journal of chromatography A* (2013) 1302:65-71. Epub 2013/07/06. doi: 10.1016/j.chroma.2013.06.005. PubMed PMID: 23827464.
34. Wang DC, Sun CH, Liu LY, Sun XH, Jin XW, Song WL, et al. Serum fatty acid profiles using GC-MS and multivariate statistical analysis: potential biomarkers of Alzheimer's disease. *Neurobiology of aging* (2012) 33(6):1057-66. Epub 2010/10/29. doi: 10.1016/j.neurobiolaging.2010.09.013. PubMed PMID: 20980076.
35. Oresic M, Hyotylainen T, Herukka SK, Sysi-Aho M, Mattila I, Seppanan-Laakso T, et al. Metabolome in progression to Alzheimer's disease. *Translational psychiatry* (2011) 1:e57. Epub 2011/01/01. doi: 10.1038/tp.2011.55. PubMed PMID: 22832349; PubMed Central PMCID: PMCPMC3309497.
36. Kaddurah-Daouk R, Rozen S, Matson W, Han X, Hulette CM, Burke JR, et al. Metabolomic changes in autopsy-confirmed Alzheimer's disease. *Alzheimer's & dementia : the journal of the Alzheimer's Association* (2011) 7(3):309-17. Epub 2010/11/16. doi: 10.1016/j.jalz.2010.06.001. PubMed PMID: 21075060; PubMed Central PMCID: PMCPMC3061205.
37. Han X, Rozen S, Boyle SH, Hellegers C, Cheng H, Burke JR, et al. Metabolomics in early Alzheimer's disease: identification of altered plasma sphingolipidome using shotgun lipidomics. *PloS one* (2011) 6(7):e21643. Epub 2011/07/23. doi: 10.1371/journal.pone.0021643. PubMed PMID: 21779331; PubMed Central PMCID: PMCPMC3136924.
38. Li NJ, Liu WT, Li W, Li SQ, Chen XH, Bi KS, et al. Plasma metabolic profiling of Alzheimer's disease by liquid chromatography/mass spectrometry. *Clinical biochemistry* (2010) 43(12):992-7. Epub 2010/05/12. doi: 10.1016/j.clinbiochem.2010.04.072. PubMed PMID: 20457143.
39. Lee SH, Kim I, Chung BC. Increased urinary level of oxidized nucleosides in patients with mild-to-moderate Alzheimer's disease. *Clinical biochemistry* (2007) 40(13-14):936-8. Epub 2007/08/19. doi: 10.1016/j.clinbiochem.2006.11.021. PubMed PMID: 17692303.
40. Paik MJ, Lee S, Cho KH, Kim KR. Urinary polyamines and N-acetylated polyamines in four patients with Alzheimer's disease as their N-ethoxycarbonyl-N-pentafluoropropionyl derivatives by gas chromatography-mass spectrometry in selected ion monitoring mode. *Analytica chimica acta* (2006) 576(1):55-60. Epub 2007/08/29. doi: 10.1016/j.aca.2006.01.070. PubMed PMID: 17723614.
41. Yoshida Y, Yoshikawa A, Kinumi T, Ogawa Y, Saito Y, Ohara K, et al. Hydroxyoctadecadienoic acid and oxidatively modified peroxiredoxins in the blood of Alzheimer's disease patients and their potential as biomarkers. *Neurobiology of aging* (2009) 30(2):174-85. Epub 2007/08/11. doi: 10.1016/j.neurobiolaging.2007.06.012. PubMed PMID: 17688973.

42. Kim KM, Jung BH, Paeng KJ, Kim I, Chung BC. Increased urinary F(2)-isoprostanes levels in the patients with Alzheimer's disease. *Brain research bulletin* (2004) 64(1):47-51. Epub 2004/07/28. doi: 10.1016/j.brainresbull.2004.04.016. PubMed PMID: 15275956.
43. Fonteh AN, Chiang J, Cipolla M, Hale J, Diallo F, Chirino A, et al. Alterations in cerebrospinal fluid glycerophospholipids and phospholipase A2 activity in Alzheimer's disease. *Journal of lipid research* (2013) 54(10):2884-97. Epub 2013/07/23. doi: 10.1194/jlr.M037622. PubMed PMID: 23868911; PubMed Central PMCID: PMCPMC3770101.
44. Wang J, Xiong S, Xie C, Markesbery WR, Lovell MA. Increased oxidative damage in nuclear and mitochondrial DNA in Alzheimer's disease. *Journal of neurochemistry* (2005) 93(4):953-62. Epub 2005/04/29. doi: 10.1111/j.1471-4159.2005.03053.x. PubMed PMID: 15857398.
45. Huang L, Hollingsworth RI, Castellani R, Zipser B. Accumulation of high-molecular-weight amylose in Alzheimer's disease brains. *Glycobiology* (2004) 14(5):409-16. Epub 2004/01/14. doi: 10.1093/glycob/cwh042. PubMed PMID: 14718371.
46. Gonzalez-Dominguez R, Garcia-Barrera T, Gomez-Ariza JL. Metabolite profiling for the identification of altered metabolic pathways in Alzheimer's disease. *Journal of pharmaceutical and biomedical analysis* (2015) 107:75-81. Epub 2015/01/13. doi: 10.1016/j.jpba.2014.10.010. PubMed PMID: 25575172.
47. Whiley L, Sen A, Heaton J, Proitsi P, Garcia-Gomez D, Leung R, et al. Evidence of altered phosphatidylcholine metabolism in Alzheimer's disease. *Neurobiology of aging* (2014) 35(2):271-8. Epub 2013/09/18. doi: 10.1016/j.neurobiolaging.2013.08.001. PubMed PMID: 24041970; PubMed Central PMCID: PMCPMC5866043.
48. Popp J, Meichsner S, Kolsch H, Lewczuk P, Maier W, Kornhuber J, et al. Cerebral and extracerebral cholesterol metabolism and CSF markers of Alzheimer's disease. *Biochemical pharmacology* (2013) 86(1):37-42. Epub 2013/01/08. doi: 10.1016/j.bcp.2012.12.007. PubMed PMID: 23291240.
49. Snowden SG, Ebshiana AA, Hye A, An Y, Pletnikova O, O'Brien R, et al. Association between fatty acid metabolism in the brain and Alzheimer disease neuropathology and cognitive performance: A nontargeted metabolomic study. *PLoS medicine* (2017) 14(3):e1002266. Epub 2017/03/23. doi: 10.1371/journal.pmed.1002266. PubMed PMID: 28323825; PubMed Central PMCID: PMCPMC5360226.
50. Guiraud SP, Montoliu I, Da Silva L, Dayon L, Galindo AN, Corthesy J, et al. High-throughput and simultaneous quantitative analysis of homocysteine-methionine cycle metabolites and co-factors in blood plasma and cerebrospinal fluid by isotope dilution LC-MS/MS. *Analytical and bioanalytical chemistry* (2017) 409(1):295-305. Epub 2016/10/21. doi: 10.1007/s00216-016-0003-1. PubMed PMID: 27757515; PubMed Central PMCID: PMCPMC5203846.
51. Graham SF, Chevallier OP, Elliott CT, Holscher C, Johnston J, McGuinness B, et al. Untargeted metabolomic analysis of human plasma indicates differentially affected polyamine and L-arginine metabolism in mild cognitive impairment subjects converting to Alzheimer's disease. *PloS one* (2015) 10(3):e0119452. Epub 2015/03/25. doi: 10.1371/journal.pone.0119452. PubMed PMID: 25803028; PubMed Central PMCID: PMCPMC4372431.
52. Kim M, Nevado-Holgado A, Whiley L, Snowden SG, Soininen H, Kloszewska I, et al. Association between Plasma Ceramides and Phosphatidylcholines and Hippocampal Brain Volume in Late Onset Alzheimer's Disease. *Journal of Alzheimer's disease : JAD* (2017)

60(3):809-17. Epub 2016/12/03. doi: 10.3233/jad-160645. PubMed PMID: 27911300; PubMed Central PMCID: PMCPMC5676755.
53. Liang Q, Liu H, Zhang T, Jiang Y, Xing H, Zhang A-h. Metabolomics-based screening of salivary biomarkers for early diagnosis of Alzheimer's disease. *RSC Advances* (2015) 5(116):96074-9. doi: 10.1039/c5ra19094k.
54. Armirotti A, Basit A, Realini N, Caltagirone C, Bossu P, Spalletta G, et al. Sample preparation and orthogonal chromatography for broad polarity range plasma metabolomics: application to human subjects with neurodegenerative dementia. *Analytical biochemistry* (2014) 455:48-54. Epub 2014/04/09. doi: 10.1016/j.ab.2014.03.019. PubMed PMID: 24708938.
55. Shah I, Petroczi A, Tabet N, Klugman A, Isaac M, Naughton DP. Low 25OH vitamin D2 levels found in untreated Alzheimer's patients, compared to acetylcholinesterase-inhibitor treated and controls. *Current Alzheimer research* (2012) 9(9):1069-76. Epub 2012/08/11. PubMed PMID: 22876849.
56. Cui Y, Liu X, Wang M, Liu L, Sun X, Ma L, et al. Lysophosphatidylcholine and amide as metabolites for detecting alzheimer disease using ultrahigh-performance liquid chromatography-quadrupole time-of-flight mass spectrometry-based metabonomics. *Journal of Neuropathology and Experimental Neurology* (2014). doi: 10.1097/NEN.0000000000000116.
57. Muguruma Y, Tsutsui H, Noda T, Akatsu H, Inoue K. Widely targeted metabolomics of Alzheimer's disease postmortem cerebrospinal fluid based on 9-fluorenylmethyl chloroformate derivatized ultra-high performance liquid chromatography tandem mass spectrometry. *Journal of chromatography B, Analytical technologies in the biomedical and life sciences* (2018) 1091:53-66. Epub 2018/06/01. doi: 10.1016/j.jchromb.2018.05.031. PubMed PMID: 29852382.
58. van der Lee SJ, Teunissen CE, Pool R, Shipley MJ, Teumer A, Chouraki V, et al. Circulating metabolites and general cognitive ability and dementia: Evidence from 11 cohort studies. *Alzheimer's & dementia : the journal of the Alzheimer's Association* (2018). Epub 2018/01/10. doi: 10.1016/j.jalz.2017.11.012. PubMed PMID: 29316447.
59. Tukiainen T, Tynkkynen T, Makinen VP, Jylanki P, Kangas A, Hokkanen J, et al. A multi-metabolite analysis of serum by 1H NMR spectroscopy: early systemic signs of Alzheimer's disease. *Biochemical and biophysical research communications* (2008) 375(3):356-61. Epub 2008/08/14. doi: 10.1016/j.bbrc.2008.08.007. PubMed PMID: 18700135.
60. Ellison EM, Abner EL, Lovell MA. Multiregional analysis of global 5-methylcytosine and 5-hydroxymethylcytosine throughout the progression of Alzheimer's disease. *Journal of neurochemistry* (2017) 140(3):383-94. Epub 2016/11/28. doi: 10.1111/jnc.13912. PubMed PMID: 27889911; PubMed Central PMCID: PMCPMC5250541.
61. Dayon L, Guiraud SP, Corthesy J, Da Silva L, Migliavacca E, Tautvydaite D, et al. One-carbon metabolism, cognitive impairment and CSF measures of Alzheimer pathology: homocysteine and beyond. *Alzheimer's research & therapy* (2017) 9(1):43. Epub 2017/06/19. doi: 10.1186/s13195-017-0270-x. PubMed PMID: 28623948; PubMed Central PMCID: PMCPMC5473969.
62. Fiandaca MS, Zhong X, Cheema AK, Orquiza MH, Chidambaram S, Tan MT, et al. Plasma 24-metabolite Panel Predicts Preclinical Transition to Clinical Stages of Alzheimer's Disease. *Frontiers in neurology* (2015) 6:237. Epub 2015/12/01. doi: 10.3389/fneur.2015.00237. PubMed PMID: 26617567; PubMed Central PMCID: PMCPMC4642213.
63. Ibanez C, Simo C, Martin-Alvarez PJ, Kivipelto M, Winblad B, Cedazo-Minguez A, et al.

Toward a predictive model of Alzheimer's disease progression using capillary electrophoresis-mass spectrometry metabolomics. *Analytical chemistry* (2012) 84(20):8532-40. Epub 2012/09/13. doi: 10.1021/ac301243k. PubMed PMID: 22967182.
64. Ronnemaa E, Zethelius B, Vessby B, Lannfelt L, Byberg L, Kilander L. Serum fatty-acid composition and the risk of Alzheimer's disease: a longitudinal population-based study. *European journal of clinical nutrition* (2012) 66(8):885-90. Epub 2012/06/21. doi: 10.1038/ejcn.2012.63. PubMed PMID: 22713770.
65. Toledo JB, Arnold M, Kastenmuller G, Chang R, Baillie RA, Han X, et al. Metabolic network failures in Alzheimer's disease: A biochemical road map. *Alzheimer's & dementia : the journal of the Alzheimer's Association* (2017) 13(9):965-84. Epub 2017/03/28. doi: 10.1016/j.jalz.2017.01.020. PubMed PMID: 28341160; PubMed Central PMCID: PMCPMC5866045.
66. Takayama T, Mochizuki T, Todoroki K, Min JZ, Mizuno H, Inoue K, et al. A novel approach for LC-MS/MS-based chiral metabolomics fingerprinting and chiral metabolomics extraction using a pair of enantiomers of chiral derivatization reagents. *Analytica chimica acta* (2015) 898:73-84. Epub 2015/11/04. doi: 10.1016/j.aca.2015.10.010. PubMed PMID: 26526912.
67. Shah I, Petroczi A, Naughton DP. Exploring the role of vitamin D in type 1 diabetes, rheumatoid arthritis, and Alzheimer disease: new insights from accurate analysis of 10 forms. *The Journal of clinical endocrinology and metabolism* (2014) 99(3):808-16. Epub 2014/01/16. doi: 10.1210/jc.2013-2872. PubMed PMID: 24423328.

**Supplementary Table 3. Quality assessment of metabolomics studies included in this systematic review by QUADOMICS**

| First author Year | Item | | | | | | | | | | | | | | | | |
|---|---|---|---|---|---|---|---|---|---|---|---|---|---|---|---|---|---|
| | 1 | 2 | 3 | 4a | 4b | 5 | 6 | 7 | 8 | 9 | 10 | 11 | 12 | 13 | 14 | 15 | 16 |
| Varma 2018 | Y | NA | Y | Y | Y | Y | Y | Y | Y | Y | Y | Y | N | Y | NA | Y | Y |
| Marksteiner 2018 | Y | NA | Y | Y | Y | Y | ? | Y | Y | Y | Y | Y | N | Y | NA | Y | Y |
| Pan 2017 | Y | NA | Y | Y | ? | Y | ? | Y | Y | Y | Y | Y | N | Y | NA | Y | N |
| Oresic 2017 | Y | NA | Y | Y | Y | Y | ? | Y | Y | Y | Y | Y | N | Y | NA | Y | N |
| Lau 2017 | Y | NA | Y | Y | Y | Y | ? | ? | Y | Y | Y | Y | Y | Y | NA | Y | N |
| Proitsi 2015 | Y | NA | Y | Y | ? | Y | ? | ? | Y | Y | Y | Y | N | Y | NA | Y | Y |
| Oberacher 2017 | Y | NA | Y | Y | Y | Y | ? | Y | Y | Y | Y | Y | N | Y | NA | Y | Y |
| De Leeuw 2017 | Y | NA | Y | Y | Y | Y | ? | Y | Y | Y | Y | Y | N | Y | NA | Y | Y |
| Zhu 2016 | Y | NA | Y | Y | ? | Y | Y | ? | Y | Y | Y | N | N | Y | NA | Y | Y |
| Xu 2016 | N | NA | Y | Y | ? | Y | Y | Y | Y | Y | Y | N | N | Y | NA | Y | N |
| Paglia 2016 | N | NA | Y | Y | ? | Y | ? | ? | ? | Y | Y | N | Y | Y | NA | Y | N |
| Nasaruddin 2016 | N | NA | Y | Y | ? | Y | ? | Y | Y | Y | Y | N | Y | Y | NA | Y | Y |
| Casanova 2016 | Y | NA | Y | Y | ? | Y | Y | Y | Y | Y | Y | Y | N | Y | NA | Y | Y |
| Gonzalez-Dominguez 2016 | Y | NA | Y | Y | Y | Y | Y | Y | Y | Y | Y | N | N | Y | NA | Y | Y |
| Ciavardelli, D. 2016 | Y | NA | Y | Y | Y | Y | Y | Y | Y | Y | Y | Y | Y | Y | NA | Y | N |
| Yoshida 2015 | Y | NA | Y | Y | Y | Y | ? | Y | Y | Y | Y | Y | N | Y | NA | Y | N |
| Vankova 2016 | Y | NA | Y | Y | Y | Y | Y | Y | ? | Y | Y | Y | N | Y | NA | Y | Y |
| Cho 2006 | Y | NA | Y | Y | ? | Y | ? | Y | ? | Y | Y | Y | N | Y | NA | Y | N |
| Koal 2015 | Y | NA | Y | ? | ? | Y | ? | Y | Y | Y | Y | Y | N | Y | NA | Y | Y |
| Klavins 2015 | Y | NA | Y | Y | Y | Y | ? | ? | Y | Y | Y | N | N | Y | NA | Y | Y |
| Gonzalez-Dominguez 2015 | Y | NA | Y | Y | Y | Y | Y | Y | Y | Y | Y | Y | N | Y | NA | Y | Y |
| Cui 2015 | Y | NA | Y | Y | Y | Y | Y | Y | ? | Y | Y | Y | N | Y | NA | Y | N |
| Ansoleaga 2015 | Y | NA | Y | Y | Y | Y | ? | Y | Y | Y | Y | Y | N | Y | NA | Y | N |
| Wisniewski 2013 | Y | NA | Y | Y | Y | Y | ? | ? | Y | Y | Y | N | N | Y | NA | Y | N |
| Gonzalez-Dominguez 2014 | Y | NA | Y | Y | Y | Y | Y | Y | Y | Y | Y | Y | N | Y | NA | Y | N |
| Gonzalez-Dominguez 2014 | Y | NA | Y | Y | Y | Y | Y | Y | Y | Y | Y | Y | N | Y | NA | Y | Y |
| Liu 2014 | Y | NA | Y | Y | Y | Y | ? | Y | Y | Y | Y | Y | N | Y | NA | Y | N |

| | | | | | | | | | | | | | | | | | |
|---|---|---|---|---|---|---|---|---|---|---|---|---|---|---|---|---|---|
| **Gonzalez-Dominguez 2014** | Y | NA | Y | Y | Y | Y | Y | Y | ? | Y | Y | Y | N | Y | NA | Y | Y |
| **Trushina E 2013** | Y | NA | Y | Y | ? | Y | ? | Y | Y | Y | Y | Y | Y | Y | NA | Y | Y |
| **Kaddurah-Daouk 2013** | Y | NA | Y | Y | Y | Y | ? | Y | Y | Y | Y | N | N | Y | NA | Y | Y |
| **Sato 2012** | ? | NA | Y | Y | ? | Y | ? | ? | Y | Y | Y | ? | N | Y | NA | Y | N |
| **Czech 2012** | Y | NA | Y | Y | Y | Y | ? | Y | Y | Y | Y | Y | Y | Y | NA | Y | Y |
| **Ibanez 2013** | Y | NA | Y | Y | ? | Y | ? | ? | Y | Y | Y | N | Y | Y | NA | Y | Y |
| **Wang 2012** | Y | NA | Y | Y | Y | Y | ? | Y | Y | Y | Y | Y | N | Y | NA | Y | N |
| **Oresic 2011** | Y | NA | Y | Y | Y | Y | ? | Y | Y | Y | Y | Y | N | Y | NA | Y | Y |
| **Kaddurah-Daouk 2011** | Y | NA | Y | Y | ? | Y | ? | Y | Y | Y | Y | Y | N | Y | NA | Y | N |
| **Han 2011** | Y | NA | Y | Y | ? | Y | ? | ? | Y | Y | Y | N | Y | Y | NA | Y | N |
| **Li 2010** | Y | NA | Y | Y | Y | Y | ? | Y | Y | Y | Y | Y | N | Y | NA | Y | N |
| **Lee 2007** | Y | NA | Y | Y | ? | Y | ? | ? | Y | Y | Y | N | Y | Y | NA | Y | N |
| **Paik 2006** | Y | NA | Y | Y | Y | Y | ? | Y | Y | Y | Y | Y | N | Y | NA | Y | N |
| **Yoshida 2009** | Y | NA | Y | Y | Y | Y | ? | Y | Y | Y | Y | Y | N | Y | NA | Y | N |
| **Kim 2004** | Y | NA | Y | Y | ? | Y | ? | ? | Y | Y | Y | N | N | Y | NA | Y | N |
| **Fonteh 2013** | ? | NA | Y | Y | ? | Y | ? | ? | Y | Y | Y | N | N | Y | NA | Y | N |
| **Wang 2005** | ? | NA | Y | Y | ? | Y | ? | ? | ? | Y | Y | N | N | Y | NA | Y | N |
| **Huang 2004** | Y | NA | Y | Y | Y | Y | Y | Y | Y | Y | Y | Y | Y | Y | NA | Y | N |
| **Gonzalez-Dominguez 2015** | Y | NA | Y | Y | Y | Y | ? | Y | Y | Y | Y | Y | Y | Y | NA | Y | Y |
| **Whiley 2014** | Y | NA | Y | Y | ? | Y | ? | Y | Y | Y | Y | Y | N | Y | NA | Y | N |
| **Popp 2013** | Y | NA | Y | Y | Y | Y | ? | Y | Y | Y | Y | Y | N | Y | NA | Y | N |
| **Snowden 2017** | Y | NA | Y | Y | ? | Y | ? | Y | Y | Y | Y | Y | Y | Y | NA | Y | Y |
| **Guiraud 2017** | N | NA | Y | Y | ? | Y | ? | ? | ? | Y | Y | N | Y | Y | NA | Y | N |
| **Graham 2015** | Y | NA | Y | Y | ? | Y | ? | Y | Y | Y | Y | Y | Y | Y | NA | Y | Y |
| **Kim 2017** | Y | NA | Y | Y | ? | Y | ? | ? | Y | Y | Y | N | N | Y | NA | Y | N |
| **Qun Liang 2015** | Y | NA | Y | Y | ? | Y | ? | Y | Y | Y | Y | Y | Y | Y | NA | Y | N |
| **Andrea Armirotti 2014** | Y | NA | Y | Y | Y | Y | ? | Y | Y | Y | Y | Y | Y | Y | NA | Y | N |
| **Iltaf Shah 2012** | Y | NA | Y | Y | Y | Y | ? | ? | Y | Y | Y | ? | N | Y | NA | Y | N |
| **Yu Cui 2014** | Y | NA | Y | Y | Y | Y | ? | Y | Y | Y | Y | Y | Y | Y | NA | Y | Y |
| **Muguruma 2018** | ? | NA | Y | Y | ? | Y | ? | ? | Y | Y | Y | N | Y | Y | NA | Y | N |
| **van der Lee 2008** | Y | NA | Y | Y | Y | Y | ? | Y | Y | Y | Y | Y | N | Y | NA | Y | Y |
| **Tukiainen 2008** | Y | NA | Y | Y | Y | Y | ? | Y | Y | Y | Y | Y | N | Y | NA | Y | N |
| **Ellison 2017** | Y | NA | Y | Y | ? | Y | ? | Y | Y | Y | Y | Y | N | Y | NA | Y | N |

| | | | | | | | | | | | | | | | | | |
|---|---|---|---|---|---|---|---|---|---|---|---|---|---|---|---|---|---|
| **Dayon 2017** | Y | NA | Y | Y | ? | Y | Y | Y | Y | Y | Y | Y | N | Y | NA | Y | Y |
| **Fiandaca 2015** | Y | NA | Y | Y | Y | Y | ? | Y | Y | Y | Y | Y | N | Y | NA | Y | Y |
| **Ibanez 2012** | Y | NA | Y | Y | ? | Y | ? | ? | ? | Y | Y | Y | Y | Y | NA | Y | Y |
| **Ronnemaa 2012** | Y | NA | Y | Y | Y | Y | Y | Y | Y | Y | Y | Y | Y | Y | NA | Y | N |
| **Toledo 2017** | Y | NA | Y | Y | Y | Y | ? | Y | Y | Y | Y | Y | N | Y | NA | Y | Y |
| **Takayama 2015** | Y | NA | Y | ? | ? | Y | ? | ? | ? | Y | Y | ? | Y | Y | NA | Y | N |
| **Shah 2014** | Y | NA | Y | Y | Y | Y | ? | ? | Y | Y | Y | Y | N | Y | NA | Y | Y |
| **Y%** | 88.1 | 0 | 100 | 97.0 | 56.7 | 100 | 22.4 | 70.2 | 86.6 | 100 | 100 | 70.2 | 31.3 | 100 | 0 | 100 | 44.8 |
| **N%** | 6.0 | 0 | 0 | 0 | 0 | 0 | 0 | 0 | 0 | 0 | 0 | 25.4 | 68.7 | 0 | 0 | 0 | 55.2 |
| **?%** | 6.0 | 0 | 0 | 3.0 | 43.3 | 0 | 77.6 | 29.9 | 13.4 | 0 | 0 | 4.5 | 0 | 0 | 0 | 0 | 0 |
| **NA%** | 0 | 100 | 0 | 0 | 0 | 0 | 0 | 0 | 0 | 0 | 0 | 0 | 0 | 0 | 100 | 0 | 0 |

**Index:** Y=criteria achieved, N=criteria not achieved, ?=Unclear, NA=not applicable.

Item 1. Were selection criteria clearly described?

2. Was the spectrum of patients' representative of patients who will receive the test in practice?

3. Was the type of sample fully described?

4. Were the procedures and timing of biological sample collection with respect to clinical factors described with enough detail?

4a. Clinical and physiological factors; 4b. Diagnostic and treatment procedures

5. Were handling and pre-analytical procedures reported in sufficient detail and similar for the whole sample? And, if differences in procedures were reported, was their effect on the results assessed?

6. Is the time period between the reference standard and the index test short enough to reasonably guarantee that the target condition did not change between the two tests?

7. Is the reference standard likely to correctly classify the target condition?

8. Did the whole sample or a random selection of the sample receive verification using a reference standard of diagnosis?

9. Did patients receive the same reference standard regardless of the result of the index test?

10. Was the execution of the index test described in sufficient detail to permit replication of the test?

11. Was the execution of the reference standard described in sufficient detail to permit its replication?

12. Were the index test results interpreted without knowledge of the results of the reference standard?

13. Were the reference standard results interpreted without knowledge of the results of the index test?

14. Were the same clinical data available when test results were interpreted as would be available when the test is used in practice?

15. Were uninterpretable/intermediate test results reported?

16. Is it likely that the presence of over-fitting was avoided?

**Supplementary Table 4. Differential metabolites of Alzheimer's disease and their reported frequencies in previous studies**

| Number | Metabolite Name | HMDB ID | Frenquency | Number | Metabolite Name | HMDB ID | Frenquency[a] |
|---|---|---|---|---|---|---|---|
| 1 | L-Tryptophan | HMDB0000929 | 8[10,8,10,27,32,46,57,66] | 416 | SM(d18:1/23:0) | HMDB0012105 | 1[8] |
| 2 | L-Phenylalanine | HMDB0000159 | 7[8,10,27,32,46,57,66] | 417 | LysoPC(17:0) | HMDB0012108 | 1[1] |
| 3 | Palmitic acid | HMDB0000220 | 7[12,21,22,28,34,46,49] | 418 | 3-Dehydrocarnitine | HMDB0012154 | 1[53] |
| 4 | L-Arginine | HMDB0000517 | 7[1,8,11,28,29,32,49] | 419 | Imidazole acetol-phosphate | HMDB0012236 | 1[29] |
| 5 | Ornithine | HMDB0000214 | 6[8,10,32,46,53,57] | 420 | L-Aspartyl-4-phosphate | HMDB0012250 | 1[56] |
| 6 | L-Methionine | HMDB0000696 | 6[8,11,26,29,30,32] | 421 | Ubiquinol-10 | HMDB0013111 | 1[41] |
| 7 | Hypoxanthine | HMDB0000157 | 5[10,11,29,49,53] | 422 | Glutaconylcarnitine | HMDB0013129 | 1[13] |
| 8 | L-Histidine | HMDB0000177 | 5[8,14,26,28,46] | 423 | PC(o-14:0/16:1(9Z)) | HMDB0013402 | 1[19] |
| 9 | L-Lactic acid | HMDB0000190 | 5[10,29,35,46,66] | 424 | PC(o-16:0/20:0) | HMDB0013406 | 1[1] |
| 10 | Oleic acid | HMDB0000207 | 5[21,22,34,46,49] | 425 | PC(o-16:0/20:4(8Z,11Z,14Z,17Z)) | HMDB0013407 | 1[1] |
| 11 | Creatinine | HMDB0000562 | 5[10,19,26,32,33] | 426 | PC(o-16:0/22:6(4Z,7Z,10Z,13Z,16Z,19Z)) | HMDB0013409 | 1[8] |
| 12 | L-Cysteine | HMDB0000574 | 5[8,10,29,32,49] | 427 | PC(o-16:1(9Z)/18:0) | HMDB0013412 | 1[8] |
| 13 | L-Valine | HMDB0000883 | 5[10,29,35,46,66] | 428 | PC(o-16:1(9Z)/18:2(9Z,12Z)) | HMDB0013413 | 1[8] |
| 14 | LysoPC(18:1(9Z)) | HMDB0002815 | 5[8,13,20,25,28] | 429 | PC(o-18:1(11Z)/18:2(9Z,12Z)) | HMDB0013425 | 1[1] |
| 15 | Cholesterol | HMDB0000067 | 4[6,46,48,49] | 430 | PC(o-18:1(9Z)/20:1(11Z)) | HMDB0013431 | 1[19] |
| 16 | Choline | HMDB0000097 | 4[11,26,28,32] | 431 | PC(o-18:1(9Z)/22:0) | HMDB0013433 | 1[1] |
| 17 | D-Glucose | HMDB0000122 | 4[10,28,45,46] | 432 | PC(o-20:0/20:4(8Z,11Z,14Z,17Z)) | HMDB0013442 | 1[7] |
| 18 | Glycine | HMDB0000123 | 4[10,20,23,50] | 433 | PC(o-22:1(13Z)/20:4(8Z,11Z,14Z,17Z)) | HMDB0013451 | 1[13] |
| 19 | L-Glutamic acid | HMDB0000148 | 4[4,10,11,28] | 434 | PC(o-22:2(13Z,16Z)/22:3(10Z,13Z,16Z)) | HMDB0013456 | 1[8] |
| 20 | L-Serine | HMDB0000187 | 4[8,10,11,32] | 435 | PC(o-24:0/18:3(6Z,9Z,12Z)) | HMDB0013458 | 1[1] |
| 21 | L-Aspartic acid | HMDB0000191 | 4[10,11,46,49] | 436 | O-Desmethylnapro | HMDB0013989 | 1[29] |

| | | | |
|---|---|---|---|
| 22 | Taurine | HMDB0000251 | 4[13,21,29,32] |
| 23 | Uric acid | HMDB0000289 | 4[11,29,32,46] |
| 24 | Urea | HMDB0000294 | 4[10,21,29,46] |
| 25 | L-Glutamine | HMDB0000641 | 4[11,28,35,46] |
| 26 | Palmitoleic acid | HMDB0003229 | 4[12,21,22,28] |
| 27 | PC(16:0/18:2(9Z,12Z)) | HMDB0007973 | 4[14,25,28,35] |
| 28 | LysoPC(18:0) | HMDB0010384 | 4[1,56,25,28] |
| 29 | 2-Hydroxybutyric acid | HMDB0000008 | 3[8,15,29] |
| 30 | Ascorbic acid | HMDB0000044 | 3[10,41,49] |
| 31 | L-Carnitine | HMDB0000062 | 3[26,28,32] |
| 32 | Creatine | HMDB0000064 | 3[21,28,29] |
| 33 | Dopamine | HMDB0000073 | 3[21,28,32] |
| 34 | Fumaric acid | HMDB0000134 | 3[10,29,49] |
| 35 | L-Tyrosine | HMDB0000158 | 3[8,32,46] |
| 36 | L-Alanine | HMDB0000161 | 3[11,19,21] |
| 37 | L-Proline | HMDB0000162 | 3[8,10,11] |
| 38 | L-Asparagine | HMDB0000168 | 3[11,26,46] |
| 39 | L-Dopa | HMDB0000181 | 3[29,32,49] |
| 40 | L-Acetylcarnitine | HMDB0000201 | 3[13,15,56] |
| 41 | myo-Inositol | HMDB0000211 | 3[10,29,32] |
| 42 | Pyruvic acid | HMDB0000243 | 3[11,29,32] |
| 43 | Sphinganine | HMDB0000269 | 3[27,38,56] |
| 44 | Uridine | HMDB0000296 | 3[29,32,33] |
| 45 | Xanthosine | HMDB0000299 | 3[11,23,30] |
| 46 | PC(16:0/16:0) | HMDB0000564 | 3[14,19,25] |
| 47 | Linoleic acid | HMDB0000673 | 3[22,28,49] |
| 48 | Myristic acid | HMDB0000806 | 3[22,28,34] |
| 49 | **Stearic acid**[b] | HMDB0000827 | 3[12,22,46] |
| 50 | Histamine | HMDB0000870 | 3[8,32,35] |
| 51 | Citrulline | HMDB0000904 | 3[8,13,32] |
| 52 | N1-Acetylspermidine | HMDB0001276 | 3[26,29,40] |
| 53 | Alpha-Linolenic acid | HMDB0001388 | 3[22,34,49] |
| 54 | Docosahexaenoic acid | HMDB0002183 | 3[28,34,49] |
| 55 | PC(18:2(9Z,12Z)/20:2(11Z,14Z)) | HMDB0008145 | 3[1,13,19] |
| 56 | LysoPC(16:0) | HMDB0010382 | 3[14,25,28] |
| 57 | LysoPC(20:5(5Z,8Z,1 | HMDB0010397 | 3[14,25,28] |

| | | | |
|---|---|---|---|
| | xen | | |
| 437 | Pyrimethamine | HMDB0014350 | 1[29] |
| 438 | Calcium Gluceptate | HMDB0014471 | 1[29] |
| 439 | Hydromorphone | HMDB0014472 | 1[29] |
| 440 | Ethopropazine | HMDB0014536 | 1[29] |
| 441 | Promazine | HMDB0014564 | 1[29] |
| 442 | Doxapram | HMDB0014701 | 1[29] |
| 443 | Ethosuximide | HMDB0014731 | 1[29] |
| 444 | Pirenzepine | HMDB0014808 | 1[29] |
| 445 | Aciclovir | HMDB0014925 | 1[29] |
| 446 | Alfentanil | HMDB0014940 | 1[29] |
| 447 | Oxazepam | HMDB0014980 | 1[29] |
| 448 | Mycophenolic acid | HMDB0015159 | 1[29] |
| 449 | Vigabatrin | HMDB0015212 | 1[29] |
| 450 | Pergolide | HMDB0015317 | 1[29] |
| 451 | Bepridil | HMDB0015374 | 1[29] |
| 452 | Acenocoumarol | HMDB0015487 | 1[29] |
| 453 | Cilastatin | HMDB0015535 | 1[29] |
| 454 | Glutamylglutamine | HMDB0028817 | 1[26] |
| 455 | Glycyl-Valine | HMDB0028854 | 1[26] |
| 456 | SM C16:1 | HMDB0029216 | 1[1] |
| 457 | Glutaral | HMDB0029599 | 1[29] |
| 458 | Arecoline | HMDB0030353 | 1[29] |
| 459 | Aromadendrin | HMDB0030847 | 1[29] |
| 460 | Acetamide | HMDB0031645 | 1[29] |
| 461 | Succinic anhydride | HMDB0032523 | 1[29] |
| 462 | Citropten | HMDB0032952 | 1[29] |
| 463 | Octadecanamide | HMDB0034146 | 1[21] |
| 464 | Hydroxypropanedioic acid | HMDB0035227 | 1[32] |
| 465 | (R)-Citronellal | HMDB0035820 | 1[29] |
| 466 | Coumaric acid | HMDB0041592 | 1[49] |
| 467 | 11,14,17-Eicosatrienoic acid | HMDB0060039 | 1[12] |
| 468 | Linoleamide | HMDB0062656 | 1[21] |
| 469 | 8-Amino-7-oxononanoate | METPA0126 | 1[29] |
| 470 | Benzene-1,2,4-triol | METPA0328 | 1[29] |
| 471 | 1-Hydroxy-2-naphthoate | METPA0374 | 1[29] |
| 472 | epsilon-Caprolacta | METPA0843 | 1[29] |

| | | | | | | | |
|---|---|---|---|---|---|---|---|
| | 1Z,14Z,17Z)) | | | | m | | |
| 58 | PPE(18:1/20:4) | / | 3[14,25,28] | 473 | Gibberellin A9 | METPA1064 | 1[29] |
| 59 | PPE(16:0/22:6) | / | 3[14,25,28] | 474 | Anthracene | METPA1122 | 1[29] |
| 60 | PPE(18:1/22:6) | / | 3[25,28,29] | 475 | 12-amino-dodecanoic acid | / | 1[29] |
| 61 | Alpha-ketoisovaleric acid | HMDB0000019 | 2[29,35] | 476 | 10-hydroxy-8E-Decene-2,4,6-triynoic acid | / | 1[29] |
| 62 | Adenine | HMDB0000034 | 2[10,49] | 477 | 10-hydroxy-2E,8E-Decadiene-4,6-diynoic acid | / | 1[29] |
| 63 | Adenosine monophosphate | HMDB0000045 | 2[10,11] | 478 | 10-hydroxy-11-dodecenoic acid | / | 1[29] |
| 64 | Beta-Alanine | HMDB0000056 | 2[10,29] | 479 | 10,11-epoxy-chlorovulone I | / | 1[29] |
| 65 | Cortisol | HMDB0000063 | 2[17,32] | 480 | 1,3-Glyceryl dinitrate | / | 1[29] |
| 66 | Deoxyguanosine | HMDB0000085 | 2[23,39] | 481 | 1,3-DIPROPYL-8-CYCLOPENTYLXANTHINE [DPCPX] | / | 1[29] |
| 67 | Dimethylglycine | HMDB0000092 | 2[29,49] | 482 | 1,2-diacyl- sn -glycerophosphoserine | / | 1[43] |
| 68 | Citric acid | HMDB0000094 | 2[10,11] | 483 | 1-(2-Pyrimidyl)piperazine | / | 1[29] |
| 69 | Gamma-Aminobutyric acid | HMDB0000112 | 2[10,49] | 484 | 1-(11E-octadecenoyl)-rac-glycerol | / | 1[29] |
| 70 | Glycolic acid | HMDB0000115 | 2[8,29] | 485 | (SM (OH) C14:1 | / | 1[1] |
| 71 | Guanosine | HMDB0000133 | 2[10,11] | 486 | (6R)-vitamin D3 6,19-(4-phenyl-1,2,4-triazoline-3,5-dione) | / | 1[56] |
| 72 | Glyceric acid | HMDB0000139 | 2[8,10] | 487 | (2-Chlorophenyl)diphenylmethane | / | 1[29] |
| 73 | L-Threonine | HMDB0000167 | 2[10,11] | 488 | (24S)-1alpha,24-dihydroxy-22-oxavitamin D3 / (24S)-1alpha,24-dihydroxy-22-oxacholecalciferol | / | 1[29] |
| 74 | L-Lysine | HMDB0000182 | 2[8,10] | 489 | (23R,25R)-1alpha,25-dihydroxyvitamin D3 26,23-lactone / (23R,25R)-1alpha,2 | / | 1[29] |

| | | | | | | | |
|---|---|---|---|---|---|---|---|
| 75 | L-Cystine | HMDB0000192 | 2[8,10] | 490 | 5-dihydroxycholecalciferol (22S)-1alpha,22,25-trihydroxy-23,24-tetradehydro-24a,24b-dihomo-20-epivitamin D3 / | / | 1[29] |
| 76 | Inosine | HMDB0000195 | 2[11,53] | 491 | (22S)-1alpha,22,2 (22R)-1alpha,22,25-trihydroxy-23,24-tetradehydro-24a,24b-dihomo-20-epivitamin D3 / | / | 1[29] |
| 77 | Indoleacetic acid | HMDB0000197 | 2[36,49] | 492 | (22R)-1alpha,22,2 (22E,24E,26E)-1alpha,26b-dihydroxy-22,23,24,25,26,26a-hexadehydro-26a,26b-dihomo-27-norvitamin D3 / | / | 1[29] |
| 78 | Norepinephrine | HMDB0000216 | 2[32,36] | 493 | (+/-)-13-HDoHE | / | 1[29] |
| 79 | Sorbitol | HMDB0000247 | 2[10,32] | 494 | 16α-hydroxyprogesterone | / | 1[17] |
| 80 | Serotonin | HMDB0000259 | 2[1,21] | 495 | 16-bromo-9-hexadecenoic acid | / | 1[56] |
| 81 | Pyroglutamic acid | HMDB0000267 | 2[29,46] | 496 | 17-Hydroxypregnenolonea | / | 1[17] |
| 82 | Xanthine | HMDB0000292 | 2[11,29] | 497 | 17-Hydroxyprogesteronea | / | 1[17] |
| 83 | Uracil | HMDB0000300 | 2[8,10] | 498 | 1a,1b-dihomo-PGJ2 | / | 1[29] |
| 84 | Allantoin | HMDB0000462 | 2[29,49] | 499 | 1α,25-dihydroxy-2α-(3-hydroxypropoxy) vitamin D3 | / | 1[56] |
| 85 | L-Arabinose | HMDB0000646 | 2[10,32] | 500 | 1alpha-25-(OH)2D2 | / | 1[67] |
| 86 | Malic acid | HMDB0000744 | 2[10,21] | 501 | 1-heptadecanoyl-2-(9Z-heptadecenoyl)-3-eicosanoyl-sn-glycerol | / | 1[29] |
| 87 | 5-Hydroxyindoleacetic acid | HMDB0000763 | 2[30,32] | 502 | 1-Hexadecylamine | / | 1[29] |
| 88 | Pseudouridine | HMDB0000767 | 2[32,39] | 503 | 1-phenantherol | / | 1[5] |
| 89 | Pregnenolone sulfate | HMDB0000774 | 2[14,17] | 504 | 1-radyl-2-acyl- sn-glycerophosphoeth | / | 1[43] |

| | | | | | | | |
|---|---|---|---|---|---|---|---|
| | | | | | anolamine | | |
| 90 | N-Acetyl-L-aspartic acid | HMDB0000812 | 2[10,11] | 505 | 2,3-dinor-8-iso-PG F2a | / | 1[8] |
| 91 | S-Adenosylhomocysteine | HMDB0000939 | 2[11,50] | 506 | 2,4-DICHLOROPHENOXYBUTYRIC ACID, METHYL ESTER | / | 1[29] |
| 92 | N-Acetylglutamic acid | HMDB0001138 | 2[10,11] | 507 | 2,4-DINITROPHENOL | / | 1[29] |
| 93 | S-Adenosylmethionine | HMDB0001185 | 2[11,50] | 508 | 2,5-Dimethoxycinnamic acid | / | 1[29] |
| 94 | Spermidine | HMDB0001257 | 2[1,40] | 509 | 20alpha-Dihydroprednisolone | / | 1[29] |
| 95 | SM(d18:1/18:0) | HMDB0001348 | 2[8,14] | 510 | 24R,25(OH)2D3 | / | 1[67] |
| 96 | Prostaglandin D2 | HMDB0001403 | 2[8,9] | 511 | 25-hydroxy-26,27-dimethylvitamin D3 / 25-hydroxy-26,27-dimethylcholecalciferol | / | 1[29] |
| 97 | Putrescine | HMDB0001414 | 2[8,40] | 512 | 25OHD2 | / | 1[55] |
| 98 | Vanylglycol | HMDB0001490 | 2[32,33] | 513 | 2-Aminoadipic | / | 1[8] |
| 99 | Eicosapentaenoic acid | HMDB0001999 | 2[47,49] | 514 | 2-Aminopropanol | / | 1[29] |
| 100 | Oleamide | HMDB0002117 | 2[14,21] | 515 | 2beta,3beta-Dihydroxy-6-oxo-5alpha-chol-7-en-24-oic Acid | / | 1[29] |
| 101 | Desmosterol | HMDB0002719 | 2[24,31] | 516 | 2-Hydroxy-3-(4-methoxyethylphenoxy)-propanoic acid | / | 1[29] |
| 102 | 1-Methyladenosine | HMDB0003331 | 2[29,39] | 517 | 2-Hydroxyglutaric acid | / | 1[10] |
| 103 | Phytosphingosine | HMDB0004610 | 2[27,38] | 518 | 2-hydroxy-N-(2-hydroxyethyl)-N,N-dimethyl-1-dodecanaminium, | / | 1[56] |
| 104 | N2,N2-Dimethylguanosine | HMDB0004824 | 2[29,39] | 519 | 2-ISOPROPYL-3-METHOXYCINNAMIC ACID | / | 1[29] |
| 105 | Ceramide (d18:1/16:0) | HMDB0004949 | 2[14,21] | 520 | 2-Naphthaleneacetic acid, 6-hydroxy | / | 1[29] |
| 106 | N-Acetylglutamine | HMDB0006029 | 2[28,56] | 521 | 2-oxo-4-hydroxy-hexanoic acid | / | 1[29] |
| 107 | PC(14:0/20:4(8Z,11Z | HMDB0007884 | 2[19,20] | 522 | 3-(a-Naphthoxy)lac | / | 1[29] |

| | | | | | | | |
|---|---|---|---|---|---|---|---|
| | ,14Z,17Z)) | | | | tic acid | | |
| 108 | PC(16:0/18:0) | HMDB0007970 | 2[25,28] | 523 | 3-(a-Naphthoxy)lactic acid glucuronide | / | 1[29] |
| 109 | PC(16:0/18:1(11Z)) | HMDB0007971 | 2[25,28] | 524 | 3,7,12-Trioxochola-1,5-dien-24-oic Acid | / | 1[29] |
| 110 | PC(16:0/18:3(6Z,9Z,12Z)) | HMDB0007974 | 2[25,28] | 525 | 3alpha,12alpha,25-trihydroxy-5beta-cholestan-7-one | / | 1[29] |
| 111 | PC(16:0/20:5(5Z,8Z,11Z,14Z,17Z)) | HMDB0007984 | 2[25,28] | 526 | 3alpha,6alpha,12alpha-Trihydroxy-7-oxo-5beta-cholan-24-oic Acid | / | 1[29] |
| 112 | PC(16:0/22:6(4Z,7Z,10Z,13Z,16Z,19Z)) | HMDB0007991 | 2[25,28] | 527 | 3beta-ACETOXYDEOXYANGOLENSIC ACID, METHYL ESTER | / | 1[29] |
| 113 | PC(16:1(9Z)/16:0) | HMDB0008001 | 2[14,25] | 528 | 3E,13Z-octadecadien-1-ol | / | 1[29] |
| 114 | PC(18:0/20:4(5Z,8Z,11Z,14Z)) | HMDB0008048 | 2[14,35] | 529 | 3-hydroxykyurenine | / | 1[57] |
| 115 | PC(18:0/22:6(4Z,7Z,10Z,13Z,16Z,19Z)) | HMDB0008057 | 2[25,28] | 530 | 3-Hydroxyphenytoin | / | 1[29] |
| 116 | PC(18:1(11Z)/20:4(5Z,8Z,11Z,14Z)) | HMDB0008081 | 2[25,28] | 531 | 3'-Methoxy-E,E-dienoestrol | / | 1[29] |
| 117 | PC(18:2(9Z,12Z)/18:1(11Z)) | HMDB0008136 | 2[25,28] | 532 | 3-oxo-4-pentenoic acid | / | 1[29] |
| 118 | PC(18:2(9Z,12Z)/18:2(9Z,12Z)) | HMDB0008138 | 2[25,28] | 533 | 4-Aminophenyl 1-thio-beta-D-glucuronide | / | 1[29] |
| 119 | PC(22:5(4Z,7Z,10Z,13Z,16Z)/14:1(9Z)) | HMDB0008657 | 2[19,20] | 534 | 4-hydroxy pelargonic acid | / | 1[29] |
| 120 | SM(d18:1/16:0) | HMDB0010169 | 2[8,14] | 535 | 4'-Hydroxyminoxidil | / | 1[29] |
| 121 | LysoPC(18:2(9Z,12Z)) | HMDB0010386 | 2[25,56] | 536 | 4-Hydroxypyridine | / | 1[29] |
| 122 | LysoPC(22:6(4Z,7Z,10Z,13Z,16Z,19Z)) | HMDB0010404 | 2[14,25] | 537 | 4-Hydroxytacrine | / | 1[29] |
| 123 | MG(18:0/0:0/0:0) | HMDB0011131 | 2[14,29] | 538 | 5-[2-(hydroxymethyl)-5-methylphenoxy]-2,2-dimethyl-Pentanoic acid (Gemfibrozil M4) | / | 1[29] |
| 124 | PC(O-16:0/18:2(9Z,12Z)) | HMDB0011151 | 2[1,8] | 539 | 5α-Androstane-3ß,17ß-diol sulfate | / | 1[17] |

| | | | |
|---|---|---|---|
| 125 | Palmitic amide | HMDB0012273 | 2[21,56] |
| 126 | PC(o-16:0/18:0) | HMDB0013405 | 2[1,19] |
| 127 | PPE(18:0/22:6) | / | 2[25,28] |
| 128 | PPE(18:0/20:4) | / | 2[25,28] |
| 129 | PPC(18:1/22:6) | / | 2[25,28] |
| 130 | PPC(16:0/22:6) | / | 2[25,28] |
| 131 | PGE2alpha dimethyl amine | / | 2[29,56] |
| 132 | LPC C20:4 | / | 2[27,38] |
| 133 | LPC C18:2 | / | 2[27,38] |
| 134 | LPC C18:1 | / | 2[27,38] |
| 135 | LPC C18:0 | / | 2[27,38] |
| 136 | LPC C16:0 | / | 2[27,38] |
| 137 | Epietiocholanolone sulfate | / | 2[17,29] |
| 138 | 1-Methylhistidine | HMDB0000001 | 1[8] |
| 139 | 2-Ketobutyric acid | HMDB0000005 | 1[35] |
| 140 | Deoxycorticosterone | HMDB0000016 | 1[32] |
| 141 | 4-Pyridoxic acid | HMDB0000017 | 1[29] |
| 142 | 3-Methoxytyramine | HMDB0000022 | 1[8] |
| 143 | (S)-3-Hydroxyisobutyric acid | HMDB0000023 | 1[8] |
| 144 | Biotin | HMDB0000030 | 1[29] |
| 145 | Carnosine | HMDB0000033 | 1[8] |
| 146 | Taurocholic acid | HMDB0000036 | 1[3] |
| 147 | Adenosine | HMDB0000050 | 1[46] |
| 148 | Argininosuccinic acid | HMDB0000052 | 1[56] |
| 149 | Androstenedione | HMDB0000053 | 1[17] |
| 150 | Bilirubin | HMDB0000054 | 1[29] |
| 540 | 5-Acetyl-4-methylthiazole | / | 1[29] |
| 541 | 5beta-Androstan-3alpha-ol-17-one sulfate | / | 1[29] |
| 542 | 5-hydroxycytosine | / | 1[44] |
| 543 | 5-hydroxyuraci | / | 1[44] |
| 544 | 5-iPF2a VI | / | 1[8] |
| 545 | 5-octadecylenic acid | / | 1[29] |
| 546 | 5ß-Androstane-3ɑ,17ß-diol sulfate | / | 1[17] |
| 547 | 6alpha-Hydroxycastasterone | / | 1[29] |
| 548 | 7,12-Dioxo-5beta-cholan-24-oic Acid | / | 1[29] |
| 549 | 7E-decen-1-ol | / | 1[29] |
| 550 | 7-hydroxy Tetranor Iloprost | / | 1[29] |
| 551 | 8,12-iPF2a IV | / | 1[8] |
| 552 | 8,13-dihydroxy-9,11-octadecadienoic acid | / | 1[29] |
| 553 | 8-Amino Caprylic acid | / | 1[29] |
| 554 | 9(S)-HODE-d4 | / | 1[29] |
| 555 | 9,12-dioxo-dodecanoic acid | / | 1[29] |
| 556 | 9-amino-nonanoic acid | / | 1[29] |
| 557 | 9-chloro-10-hydroxy-hexadecanoic acid | / | 1[29] |
| 558 | 9R-hydroxy-10E-octadecenoic acid | / | 1[29] |
| 559 | Adriamycinone | / | 1[29] |
| 560 | AGELASINE | / | 1[29] |
| 561 | Ala Phe Arg | / | 1[29] |
| 562 | all-cis-13,16-docosadienoic acid | / | 1[12] |
| 563 | Allocortol | / | 1[29] |
| 564 | Allopregnanolone sulfate | / | 1[17] |
| 565 | AMBELLINE | / | 1[29] |

| | | | | | | | |
|---|---|---|---|---|---|---|---|
| 151 | Acetoacetic acid | HMDB0000060 | 1[29] | 566 | Aminobutanal | / | 1[49] |
| 152 | Pipecolic acid | HMDB0000070 | 1[46] | 567 | ANISOMYCIN | / | 1[29] |
| 153 | cis-Aconitic acid | HMDB0000072 | 1[8] | 568 | Arg Cys Cys | / | 1[29] |
| 154 | Cytidine | HMDB0000089 | 1[56] | 569 | Asp Glu | / | 1[29] |
| 155 | Deoxyadenosine | HMDB0000101 | 1[29] | 570 | Benzaldehyde, p-amino-, thiosemicarbazone | / | 1[29] |
| 156 | Galactitol | HMDB0000107 | 1[32] | 571 | BENZANTHRONE | / | 1[29] |
| 157 | Fructose 6-phosphate | HMDB0000124 | 1[10] | 572 | BERGENIN | / | 1[29] |
| 158 | Glutathione | HMDB0000125 | 1[30] | 573 | beta-D-Glucopyran osiduronic acid | / | 1[29] |
| 159 | Glycerol 3-phosphate | HMDB0000126 | 1[10] | 574 | beta-vinyl acrylic acid | / | 1[29] |
| 160 | Glycerol | HMDB0000131 | 1[10] | 575 | bicyclo-PGE2 | / | 1[29] |
| 161 | Guanine | HMDB0000132 | 1[23] | 576 | Bifemelane (M4) | / | 1[29] |
| 162 | Ethanolamine | HMDB0000149 | 1[10] | 577 | Bis (2-hydroxypropyl) amine | / | 1[29] |
| 163 | L-Malic acid | HMDB0000156 | 1[11] | 578 | Butanediol | / | 1[10] |
| 164 | L-Isoleucine | HMDB0000172 | 1[8] | 579 | C10:0-carnitine | / | 1[26] |
| 165 | Inosinic acid | HMDB0000175 | 1[11] | 580 | C10:1-carnitine | / | 1[26] |
| 166 | Isocitric acid | HMDB0000193 | 1[46] | 581 | C10:2 | / | 1[19] |
| 167 | Methylmalonic acid | HMDB0000202 | 1[8] | 582 | C16-OH | / | 1[19] |
| 168 | Oxoglutaric acid | HMDB0000208 | 1[46] | 583 | C20:2 | / | 1[22] |
| 169 | Pantothenic acid | HMDB0000210 | 1[11] | 584 | C22:6 | / | 1[22] |
| 170 | Myo-inositol 1-phosphate | HMDB0000213 | 1[10] | 585 | C3-DC-M/C5-OH | / | 1[19] |
| 171 | N-Acetyl-D-glucosa mine | HMDB0000215 | 1[10] | 586 | C5-Carnitine | / | 1[26] |
| 172 | L-Palmitoylcarnitine | HMDB0000222 | 1[14] | 587 | C8-carnitine | / | 1[26] |
| 173 | Phenol | HMDB0000228 | 1[29] | 588 | CARYLOPHYLLE NE OXIDE | / | 1[29] |
| 174 | Pregnenolone | HMDB0000253 | 1[17] | 589 | Catalpol | / | 1[29] |
| 175 | Succinic acid | HMDB0000254 | 1[11] | 590 | CER(d18:1/16:1) | / | 1[21] |
| 176 | Sarcosine | HMDB0000271 | 1[8] | 591 | CER(d18:1/18:0)-1 P | / | 1[21] |
| 177 | Vanillylmandelic acid | HMDB0000291 | 1[30] | 592 | CER(d18:1/18:1) | / | 1[21] |
| 178 | **16-a-Hydroxypregne nolone**[b] | HMDB0000315 | 1[17] | 593 | CER(d18:1/24:1) | / | 1[21] |
| 179 | 3-Amino-2-piperidon e | HMDB0000323 | 1[29] | 594 | Cer16:0 | / | 1[52] |
| 180 | Isohomovanillic acid | HMDB0000333 | 1[29] | 595 | Cer18:0 | / | 1[52] |
| 181 | 2-Methylbutyrylglyci ne | HMDB0000339 | 1[29] | 596 | Cer24:1 | / | 1[52] |

| | | | | | | | |
|---|---|---|---|---|---|---|---|
| 182 | 2-Octenedioic acid | HMDB0000341 | 1[29] | 597 | cholesteryl ester 32:0 | / | 1[6] |
| 183 | Erythrono-1,4-lactone | HMDB0000349 | 1[29] | 598 | cholesteryl ester 32:4 | / | 1[6] |
| 184 | 3-Hydroxybutyric acid | HMDB0000357 | 1[10] | 599 | cholesteryl ester 33:6 | / | 1[6] |
| 185 | 17a-Hydroxypregnenolone | HMDB0000363 | 1[17] | 600 | cholesteryl ester 34:0 | / | 1[6] |
| 186 | 17-Hydroxyprogesterone | HMDB0000374 | 1[17] | 601 | cholesteryl ester 34:6 | / | 1[6] |
| 187 | 2-Hydroxy-3-methylbutyric acid | HMDB0000407 | 1[10] | 602 | cholesteryl ester40:4 | / | 1[6] |
| 188 | 2-Methyl-3-ketovaleric acid | HMDB0000408 | 1[29] | 603 | clavirin I | / | 1[29] |
| 189 | Adipic acid | HMDB0000448 | 1[49] | 604 | Clonidine Metabolite 3 | / | 1[29] |
| 190 | 5-Hydroxy-L-tryptophan | HMDB0000472 | 1[29] | 605 | cLPA C18:0 | / | 1[8] |
| 191 | 3-Methylhistidine | HMDB0000479 | 1[29] | 606 | cLPA C18:1 | / | 1[8] |
| 192 | Caprylic acid | HMDB0000482 | 1[28] | 607 | conjugated 5α-androstane-3ß,17ß-diol | / | 1[17] |
| 193 | 7-Hydroxyoctanoic acid | HMDB0000486 | 1[29] | 608 | conjugated 5ß-androstane-3α,17ß-diol | / | 1[17] |
| 194 | 3-Methyl-2-oxovaleric acid | HMDB0000491 | 1[29] | 609 | Conjugated 5ß-pregnane-3α,20α-diol | / | 1[17] |
| 195 | Aminoadipic acid | HMDB0000510 | 1[8] | 610 | Conjugated pregnanolone | / | 1[17] |
| 196 | Capric acid | HMDB0000511 | 1[28] | 611 | COTARNINE | / | 1[29] |
| 197 | 8-Hydroxyadenine | HMDB0000542 | 1[44] | 612 | Cyclopenthiazide | / | 1[29] |
| 198 | Elaidic acid | HMDB0000573 | 1[12] | 613 | Cys Cys Tyr | / | 1[29] |
| 199 | PC(18:1(9Z)/18:1(9Z)) | HMDB0000593 | 1[25] | 614 | Cys Tyr Cys | / | 1[29] |
| 200 | Erythronic acid | HMDB0000613 | 1[10] | 615 | d18:1/16:0 | / | 1[54] |
| 201 | D-Ribulose 5-phosphate | HMDB0000618 | 1[11] | 616 | d18:1/18:0 | / | 1[54] |
| 202 | Cholic acid | HMDB0000619 | 1[3] | 617 | d18:1/24:1 | / | 1[54] |
| 203 | Glutaconic acid | HMDB0000620 | 1[29] | 618 | decenoylcarnitine | / | 1[15] |
| 204 | D-Leucic acid | HMDB0000624 | 1[29] | 619 | Deoxyflurouridine | / | 1[49] |
| 205 | Gluconic acid | HMDB0000625 | 1[49] | 620 | diacetyl-spermidine | / | 1[57] |
| 206 | Citraconic acid | HMDB0000634 | 1[29] | 621 | diacetyl-spermine | / | 1[57] |
| 207 | Dodecanoic acid | HMDB0000638 | 1[28] | 622 | Diglycolic acid | / | 1[29] |

| | | | | | | | |
|---|---|---|---|---|---|---|---|
| 208 | Clionasterol | HMDB0000649 | 1[35] | 623 | DIHYDROFISSIN OLIDE | / | 1[29] |
| 209 | Decanoylcarnitine | HMDB0000651 | 1[15] | 624 | dihydroxyacetone phosphate (DHAP) | / | 1[29] |
| 210 | D-Fructose | HMDB0000660 | 1[10] | 625 | Disaccharide | / | 1[10] |
| 211 | L-Kynurenine | HMDB0000684 | 1[57] | 626 | Docosahexanoic acid | / | 1[12] |
| 212 | L-Leucine | HMDB0000687 | 1[8] | 627 | dodecanamide | / | 1[29] |
| 213 | Isovalerylcarnitine/2-Methylbutyroylcarnitine/Valerylcarnitine | HMDB0000688/HMDB0000378/HMDB0013128 | 1[13] | 628 | dodecenoylcarnitine | / | 1[15] |
| 214 | 4-Hydroxybutyric acid | HMDB0000710 | 1[10] | 629 | EMODIC ACID | / | 1[29] |
| 215 | L-Homoserine | HMDB0000719 | 1[8] | 630 | EPICATECHIN PENTAACETATE | / | 1[29] |
| 216 | 4-Hydroxyproline | HMDB0000725 | 1[11] | 631 | ethyl 3-cyano-2,3-bis (2,5,-dimethyl-3-thienyl)-acrylate | / | 1[5] |
| 217 | Alpha-Hydroxyisobutyric acid | HMDB0000729 | 1[29] | 632 | EUPATORIOCHROMENE | / | 1[29] |
| 218 | Hydroxykynurenine | HMDB0000732 | 1[57] | 633 | fapyadenine | / | 1[44] |
| 219 | Indoleacrylic acid | HMDB0000734 | 1[29] | 634 | Fapyguanine | / | 1[44] |
| 220 | Isobutyryl-L-carnitine | HMDB0000736 | 1[56] | 635 | Furafylline | / | 1[29] |
| 221 | Indole | HMDB0000738 | 1[29] | 636 | Galabiosylceramide (d18:1/12:0) | / | 1[29] |
| 222 | 3-Hydroxyisovaleric acid | HMDB0000754 | 1[8] | 637 | Galbeta1-4GlcNAc beta-Sp | / | 1[29] |
| 223 | Lithocholic acid | HMDB0000761 | 1[2] | 638 | gamma-Hydroxyphenylbutazone glucuronide | / | 1[29] |
| 224 | Mannitol | HMDB0000765 | 1[29] | 639 | GlcAbeta-Cer(d18:1/18:0) | / | 1[29] |
| 225 | Phenyllactic acid | HMDB0000779 | 1[53] | 640 | Gln | / | 1[19] |
| 226 | Azelaic acid | HMDB0000784 | 1[56] | 641 | Gln Pro Lys | / | 1[29] |
| 227 | L-Octanoylcarnitine | HMDB0000791 | 1[15] | 642 | Glu Ser | / | 1[29] |
| 228 | Monoiodothyronine | HMDB0000793 | 1[56] | 643 | GPCh | / | 1[28] |
| 229 | Normetanephrine | HMDB0000819 | 1[32] | 644 | GPEtn(13:0/15:0)[U] | / | 1[29] |
| 230 | Phenylacetylglycine | HMDB0000821 | 1[14] | 645 | GPGro(17:0/20:4(5Z,8Z,11Z,14Z)) | / | 1[29] |
| 231 | Propionylcarnitine | HMDB0000824 | 1[1] | 646 | GPSer(16:0/18:1(11Z)) | / | 1[29] |

| | | | | | | | |
|---|---|---|---|---|---|---|---|
| 232 | Pentadecanoic acid | HMDB0000826 | 1[12] | 647 | GPSer(18:0/20:4(5Z,8Z,11Z,14Z)) | / | 1[29] |
| 233 | Stearoylcarnitine | HMDB0000848 | 1[14] | 648 | Granisetron metabolite 1 | / | 1[29] |
| 234 | Ribonic acid | HMDB0000867 | 1[32] | 649 | Gualenate | / | 1[29] |
| 235 | Vitamin D3 | HMDB0000876 | 1[55] | 650 | Guanidobutanoate | / | 1[49] |
| 236 | Ergocalciferol | HMDB0000900 | 1[55] | 651 | Gummiferol | / | 1[29] |
| 237 | DL-2-Aminooctanoic acid | HMDB0000991 | 1[29] | 652 | H1 | / | 1[1] |
| 238 | Biliverdin | HMDB0001008 | 1[29] | 653 | HAEMATOXYLIN | / | 1[29] |
| 239 | Dehydroepiandrosterone sulfate | HMDB0001032 | 1[18] | 654 | Hcy-Cys | / | 1[26] |
| 240 | Arachidonic acid | HMDB0001043 | 1[49] | 655 | hexadecasphinganine | / | 1[38] |
| 241 | 2'-Deoxyguanosine 5'-monophosphate | HMDB0001044 | 1[23] | 656 | Hex-CER(d18:1/16:0) | / | 1[14] |
| 242 | Leukotriene B4 | HMDB0001085 | 1[28] | 657 | Hex-CER(d18:1/18:0) | / | 1[14] |
| 243 | 2-Aminobenzoic acid | HMDB0001123 | 1[57] | 658 | HYDROLYSIS PRODUCT OF BUSSEIN | / | 1[29] |
| 244 | Prostaglandin F2a | HMDB0001139 | 1[42] | 659 | inoleyl-carnitine | / | 1[14] |
| 245 | Pyruvaldehyde | HMDB0001167 | 1[29] | 660 | Isoquinoline N-oxide | / | 1[29] |
| 246 | 4-Aminophenol | HMDB0001169 | 1[29] | 661 | KOBUSONE | / | 1[29] |
| 247 | Lathosterol | HMDB0001170 | 1[48] | 662 | Lac-CER(d18:1/14:0) | / | 1[14] |
| 248 | Adenosine diphosphate ribose | HMDB0001178 | 1[11] | 663 | Lac-CER(d18:1/16:0) | / | 1[14] |
| 249 | N1-Acetylspermine | HMDB0001186 | 1[40] | 664 | Lac-CER(d18:1/16:1) | / | 1[14] |
| 250 | Flavin adenine dinucleotide | HMDB0001248 | 1[29] | 665 | L-Alanine n-butyl ester | / | 1[29] |
| 251 | N-Acetylarylamine | HMDB0001250 | 1[29] | 666 | Lecanoric acid | / | 1[29] |
| 252 | Lanosterin | HMDB0001251 | 1[48] | 667 | Leu Pro | / | 1[26] |
| 253 | Spermine | HMDB0001256 | 1[40] | 668 | L-gamma-Cyano-gamma-aminobutyric acid | / | 1[29] |
| 254 | Dehydroascorbic acid | HMDB0001264 | 1[29] | 669 | L-Glutamic acid dibutyl ester | / | 1[29] |
| 255 | ADP | HMDB0001341 | 1[11] | 670 | Linolenamide | / | 1[21] |
| 256 | Purine | HMDB0001366 | 1[29] | 671 | LPA C14:0 | / | 1[8] |
| 257 | Diaminopimelic acid | HMDB0001370 | 1[29] | 672 | LPA C16 | / | 1[8] |
| 258 | Vaporole | HMDB0001382 | 1[29] | 673 | LPA C18 | / | 1[8] |

| | | | | | | | |
|---|---|---|---|---|---|---|---|
| 259 | Sphinganine 1-phosphate | HMDB0001383 | 1[53] | 674 | LPA C18:3 | / | 1[8] |
| 260 | Guanosine monophosphate | HMDB0001397 | 1[11] | 675 | LPA C20:1 | / | 1[8] |
| 261 | Glucose 6-phosphate | HMDB0001401 | 1[10] | 676 | LPA C22:4 | / | 1[8] |
| 262 | 24-Hydroxycholesterol | HMDB0001419 | 1[48] | 677 | LPC (16:1) | / | 1[14] |
| 263 | Estrone sulfate | HMDB0001425 | 1[29] | 678 | LPC (20:5) | / | 1[14] |
| 264 | Allopregnanolone | HMDB0001449 | 1[17] | 679 | LPC (22:5) | / | 1[14] |
| 265 | Alloepipregnanolone | HMDB0001455 | 1[17] | 680 | LPE (16:0) | / | 1[14] |
| 266 | Prostaglandin F2b | HMDB0001483 | 1[42] | 681 | LPE (18:2) | / | 1[14] |
| 267 | Octane | HMDB0001485 | 1[29] | 682 | LPPC(18:0) | / | 1[25] |
| 268 | Queuine | HMDB0001495 | 1[29] | 683 | LPPC(18:1) | / | 1[25] |
| 269 | Asymmetric dimethylarginine | HMDB0001539 | 1[26] | 684 | lyosPC a C16:0 | / | 1[19] |
| 270 | D-Ribose 5-phosphate | HMDB0001548 | 1[10] | 685 | MaR1 | / | 1[9] |
| 271 | Progesterone | HMDB0001830 | 1[27] | 686 | Mebeverine | / | 1[29] |
| 272 | Aminopterin | HMDB0001833 | 1[56] | 687 | MERBROMIN | / | 1[29] |
| 273 | N-Acryloylglycine | HMDB0001843 | 1[56] | 688 | Met Ala His | / | 1[29] |
| 274 | D-threo-Isocitric acid | HMDB0001874 | 1[29] | 689 | Met Glu Cys | / | 1[29] |
| 275 | Alpha-Tocopherol | HMDB0001893 | 1[41] | 690 | Met Glu Lys | / | 1[29] |
| 276 | Salicylic acid | HMDB0001895 | 1[32] | 691 | Met His Lys | / | 1[29] |
| 277 | Methionine sulfoxide | HMDB0002005 | 1[57] | 692 | Met Trp Gln | / | 1[29] |
| 278 | Ureidoisobutyric acid | HMDB0002031 | 1[29] | 693 | Methoxsalen Metabolite | / | 1[29] |
| 279 | 8-Hydroxyguanine | HMDB0002032 | 1[44] | 694 | METHYL 7-DESHYDROXY PYROGALLIN-4-CARBOXYLATE | / | 1[29] |
| 280 | 2-Pyrrolidinone | HMDB0002039 | 1[29] | 695 | methyl 8-[2-(2-formyl-vinyl)-3-hydroxy-5-oxo-cyclopentyl]-octanoate | / | 1[29] |
| 281 | N-Acetylputrescine | HMDB0002064 | 1[29] | 696 | Methylheptadecadiynoic acid | / | 1[49] |
| 282 | 27-Hydroxycholesterol | HMDB0002103 | 1[48] | 697 | Methyl-phosphate | / | 1[10] |
| 283 | Phosphoric acid | HMDB0002142 | 1[10] | 698 | Methylstearate | / | 1[49] |
| 284 | N8-Acetylspermidine | HMDB0002189 | 1[40] | 699 | N,N-Didemethylchlorpromazine | / | 1[29] |
| 285 | Picolinic acid | HMDB0002243 | 1[21] | 700 | N1,N4-Diacetylsulfanilamide | / | 1[29] |
| 286 | Dodecanoylcarnitine | HMDB0002250 | 1[15] | 701 | N16:0 | / | 1[37] |

| | | | | | | | |
|---|---|---|---|---|---|---|---|
| 287 | Heptadecanoic acid | HMDB0002259 | 1[12] | 702 | N17:1 | / | 1[37] |
| 288 | N-Acetylcadaverine | HMDB0002284 | 1[40] | 703 | N18:0 | / | 1[37] |
| 289 | Cadaverine | HMDB0002322 | 1[40] | 704 | N1-acetylputrescine | / | 1[40] |
| 290 | Stearaldehyde | HMDB0002384 | 1[49] | 705 | N1N12-diacetyl-spe rmine | / | 1[57] |
| 291 | Trimethyltridecanoic acid | HMDB0002396 | 1[56] | 706 | N1N8-diacetyl-sper midine | / | 1[57] |
| 292 | Terephthalic acid | HMDB0002428 | 1[29] | 707 | N20:0 | / | 1[37] |
| 293 | Beta-Glycerophospho ric acid | HMDB0002520 | 1[10] | 708 | N21:0(ceramide) | / | 1[37] |
| 294 | 6-Hydroxynicotinic acid | HMDB0002658 | 1[29] | 709 | N21:0(Sphingomye lin) | / | 1[37] |
| 295 | 1-Methylinosine | HMDB0002721 | 1[56] | 710 | N22:0 | / | 1[37] |
| 296 | Androsterone sulfate | HMDB0002759 | 1[17] | 711 | N22:1 | / | 1[37] |
| 297 | Testosterone sulfate | HMDB0002833 | 1[29] | 712 | N23:0(ceramide) | / | 1[37] |
| 298 | D-Xylitol | HMDB0002917 | 1[10] | 713 | N23:0(Sphingomye lin) | / | 1[37] |
| 299 | Erythritol | HMDB0002994 | 1[32] | 714 | N23:1 | / | 1[37] |
| 300 | O-Acetylserine | HMDB0003011 | 1[8] | 715 | N24:0 | / | 1[37] |
| 301 | Isoacitretin | HMDB0003039 | 1[29] | 716 | N24:1 | / | 1[37] |
| 302 | Lactaldehyde | HMDB0003052 | 1[29] | 717 | N24:2 | / | 1[37] |
| 303 | 20a-Dihydroprogester one | HMDB0003069 | 1[17] | 718 | N26:0 | / | 1[37] |
| 304 | Quinic acid | HMDB0003072 | 1[32] | 719 | N28:2 | / | 1[37] |
| 305 | Gamma-Linolenic acid | HMDB0003073 | 1[22] | 720 | N-Acetyl-p-benzoq uinonimine | / | 1[29] |
| 306 | Sedoheptulose | HMDB0003219 | 1[29] | 721 | Nalorphine | / | 1[29] |
| 307 | Acetoin | HMDB0003243 | 1[29] | 722 | Ne-Methyl-L-lysine | / | 1[29] |
| 308 | 8-Hydroxy-deoxygua nosine | HMDB0003333 | 1[39] | 723 | N-gamma-Acetyl-N -2-formyl-5-methox ykynurenamine | / | 1[29] |
| 309 | Symmetric dimethylarginine | HMDB0003334 | 1[13] | 724 | NO2-aLA (C18:3) | / | 1[8] |
| 310 | IDP | HMDB0003335 | 1[23] | 725 | NO2-OA (C18:1) | / | 1[8] |
| 311 | Pectin | HMDB0003402 | 1[32] | 726 | Norcodeine | / | 1[29] |
| 312 | Tryptophanol | HMDB0003447 | 1[29] | 727 | O-BENZYL-l-SERI NE | / | 1[29] |
| 313 | 4-Guanidinobutanoic acid | HMDB0003464 | 1[29] | 728 | OH-N24:1 | / | 1[37] |
| 314 | Carbamic acid | HMDB0003551 | 1[49] | 729 | OH-N24:2 | / | 1[37] |
| 315 | Dethiobiotin | HMDB0003581 | 1[56] | 730 | oleylcarnitine | / | 1[14] |
| 316 | Resolvin D5 | HMDB0004038 | 1[9] | 731 | Oxoarginine | / | 1[49] |
| 317 | D-Threitol | HMDB0004136 | 1[10] | 732 | OXOLINIC ACID | / | 1[29] |
| 318 | L-Urobilin | HMDB0004159 | 1[29] | 733 | Palmitoleamide | / | 1[21] |

| | | | | | | | |
|---|---|---|---|---|---|---|---|
| 319 | 3,3',4'5-Tetrahydroxy stilbene | HMDB0004215 | 1[29] | 734 | PC 16:0/20:5 | / | 1[47] |
| 320 | Pyrrole-2-carboxylic acid | HMDB0004230 | 1[29] | 735 | PC aa C32:3 | / | 1[13] |
| 321 | Imidazolone | HMDB0004363 | 1[29] | 736 | PC aa C36.1 | / | 1[19] |
| 322 | Etiocholanolone glucuronide | HMDB0004484 | 1[56] | 737 | PC aa C40:5 | / | 1[1] |
| 323 | 3-Methyluridine | HMDB0004813 | 1[39] | 738 | PC(18:2/20:5） | / | 1[25] |
| 324 | Proline betaine | HMDB0004827 | 1[26] | 739 | PC(32a:0) | / | 1[43] |
| 325 | Ceramide (d18:1/18:0) | HMDB0004950 | 1[21] | 740 | PC(34a:1) | / | 1[43] |
| 326 | Glucosylceramide (d18:1/20:0) | HMDB0004973 | 1[29] | 741 | PC(34p:0/34e:1) | / | 1[43] |
| 327 | 8-Isoprostaglandin F2a | HMDB0005083 | 1[8] | 742 | PC(36a:0/38p:6) | / | 1[43] |
| 328 | TG(16:0/16:0/16:0) | HMDB0005356 | 1[8] | 743 | PC(36a:1) | / | 1[43] |
| 329 | TG(16:0/16:0/18:0) | HMDB0005357 | 1[8] | 744 | PC(38a:5) | / | 1[43] |
| 330 | TG(16:0/16:0/16:1(9Z)) | HMDB0005359 | 1[8] | 745 | PC(38a:6) | / | 1[43] |
| 331 | TG(16:0/16:0/18:1(9Z)) | HMDB0005360 | 1[8] | 746 | PC(O-18:0/18:2) | / | 1[35] |
| 332 | TG(16:0/16:0/20:1(11Z)) | HMDB0005361 | 1[8] | 747 | PC(O-36:6) | / | 1[8] |
| 333 | TG(16:0/16:0/18:2(9Z,12Z)) | HMDB0005362 | 1[8] | 748 | PC16:0/22:6 | / | 1[47] |
| 334 | TG(16:0/16:0/20:4(5Z,8Z,11Z,14Z)) | HMDB0005363 | 1[8] | 749 | PC18:0/22:6 | / | 1[47] |
| 335 | TG(16:0/18:0/18:2(9Z,12Z)) | HMDB0005369 | 1[8] | 750 | PC36:5 | / | 1[52] |
| 336 | TG(16:0/16:1(9Z)/16:1(9Z)) | HMDB0005376 | 1[8] | 751 | PC38:6 | / | 1[52] |
| 337 | TG(16:0/16:1(9Z)/18:2(9Z,12Z)) | HMDB0005379 | 1[8] | 752 | PC40:6 | / | 1[52] |
| 338 | TG(16:0/16:1(9Z)/20:4(5Z,8Z,11Z,14Z)) | HMDB0005380 | 1[8] | 753 | PD1 | / | 1[9] |
| 339 | TG(16:0/18:1(9Z)/18:2(9Z,12Z)) | HMDB0005384 | 1[8] | 754 | PE(40p:4/40e:5) | / | 1[43] |
| 340 | TG(16:0/18:1(9Z)/20:4(5Z,8Z,11Z,14Z)) | HMDB0005385 | 1[8] | 755 | PE(O-38:5) | / | 1[8] |
| 341 | TG(16:0/18:2(9Z,12Z)/20:4(5Z,8Z,11Z,14Z)) | HMDB0005391 | 1[8] | 756 | Pentonic acid A | / | 1[10] |
| 342 | TG(16:0/20:4(5Z,8Z,11Z,14Z)/20:4(5Z,8Z | HMDB0005392 | 1[8] | 757 | Pentonic acid B | / | 1[10] |

| | | | | | | | |
|---|---|---|---|---|---|---|---|
| | ,11Z,14Z)) | | | | | | |
| 343 | TG(16:1(9Z)/16:1(9Z)/16:1(9Z)) | HMDB0005432 | 1[8] | 758 | Phe Ala Arg | / | 1[29] |
| 344 | TG(16:1(9Z)/16:1(9Z)/18:2(9Z,12Z)) | HMDB0005435 | 1[8] | 759 | Phe Phe | / | 1[26] |
| 345 | TG(18:1(9Z)/18:2(9Z,12Z)/20:4(5Z,8Z,11Z,14Z)) | HMDB0005462 | 1[8] | 760 | Phenoperidine | / | 1[29] |
| 346 | TG(18:1(9Z)/20:4(5Z,8Z,11Z,14Z)/20:4(5Z,8Z,11Z,14Z)) | HMDB0005463 | 1[8] | 761 | phosphatidylethanolamine(18:0/20:4(5Z,8Z,11Z,14Z))[U] | / | 1[] |
| 347 | TG(18:2(9Z,12Z)/20:4(5Z,8Z,11Z,14Z)/20:4(5Z,8Z,11Z,14Z)) | HMDB0005476 | 1[8] | 762 | phosphatidylethanolamine(18:1(11Z)/18:1(9Z))[U] | / | 1[29] |
| 348 | scyllo-Inositol | HMDB0006088 | 1[32] | 763 | phosphatidylethanolamine(20:4(5Z,8Z,11Z,14Z)/20:4(5Z,8Z,11Z,14Z))[U] | / | 1[29] |
| 349 | gamma-Glutamylalanine | HMDB0006248 | 1[8] | 764 | phosphatidylethanolamine(O-16:0/22:5(4Z,7Z,10Z,13Z,16Z)) | / | 1[29] |
| 350 | 5a-Cholest-8-en-3b-ol | HMDB0006841 | 1[49] | 765 | phosphatidylethanolamineNMe(18:1(9Z)/18:1(9Z))[U] | / | 1[29] |
| 351 | DG(14:1(9Z)/16:0/0:0) | HMDB0007040 | 1[21] | 766 | phosphatidylethanolamineNMe(18:2(9Z,12Z)/18:2(9Z,12Z))[U] | / | 1[29] |
| 352 | DG(14:1(9Z)/22:2(13Z,16Z)/0:0) | HMDB0007059 | 1[8] | 767 | Pizotyline | / | 1[29] |
| 353 | DG(16:0/18:1(11Z)/0:0) | HMDB0007101 | 1[21] | 768 | PPC(16:0/18:2) | / | 1[14] |
| 354 | DG(16:0/18:3(6Z,9Z,12Z)/0:0) | HMDB0007104 | 1[21] | 769 | PPC(16:0/22:5) | / | 1[28] |
| 355 | DG(16:0/20:4(5Z,8Z,11Z,14Z)/0:0) | HMDB0007112 | 1[21] | 770 | PPC(18:0/20:4) | / | 1[25] |
| 356 | DG(18:2(9Z,12Z)/18:1(11Z)/0:0) | HMDB0007246 | 1[21] | 771 | PPC(18:0/22:6) | / | 1[25] |
| 357 | DG(18:3(6Z,9Z,12Z)/18:2(9Z,12Z)/0:0) | HMDB0007277 | 1[21] | 772 | PPC(18:1/20:4) | / | 1[25] |
| 358 | DG(18:3(6Z,9Z,12Z)/18:3(6Z,9Z,12Z)/0:0) | HMDB0007278 | 1[21] | 773 | PPE(16.0/22.6) | / | 1[25] |
| 359 | DG(22:4(7Z,10Z,13Z | HMDB0007700 | 1[29] | 774 | PPE(16:0/20:4) | / | 1[25] |

| | | | |
|---|---|---|---|
| | ,16Z)/22:5(7Z,10Z,13Z,16Z,19Z)/0:0) | | |
| 360 | PC(14:0/20:1(11Z)) | HMDB0007879 | 1[19] |
| 361 | PC(14:0/20:3(8Z,11Z,14Z)) | HMDB0007882 | 1[13] |
| 362 | PC(15:0/18:1(11Z)) | HMDB0007938 | 1[14] |
| 363 | PC(15:0/18:2(9Z,12Z)) | HMDB0007940 | 1[14] |
| 364 | PC(16:0/20:3(5Z,8Z,11Z)) | HMDB0007980 | 1[14] |
| 365 | PC(16:0/20:3(8Z,11Z,14Z)) | HMDB0007981 | 1[13] |
| 366 | PC(16:0/20:4(5Z,8Z,11Z,14Z)) | HMDB0007982 | 1[35] |
| 367 | **PC(16:0/22:5(4Z,7Z,10Z,13Z,16Z))**[b] | HMDB0007989 | 1[25] |
| 368 | PC(16:1(9Z)/16:1(9Z)) | HMDB0008002 | 1[25] |
| 369 | PC(16:1(9Z)/18:3(6Z,9Z,12Z)) | HMDB0008007 | 1[25] |
| 370 | PC(16:1(9Z)/22:6(4Z,7Z,10Z,13Z,16Z,19Z)) | HMDB0008023 | 1[14] |
| 371 | PC(18:0/18:0) | HMDB0008036 | 1[25] |
| 372 | PC(18:0/18:2(9Z,12Z)) | HMDB0008039 | 1[35] |
| 373 | PC(18:0/20:3(5Z,8Z,11Z)) | HMDB0008046 | 1[25] |
| 374 | PC(18:1(11Z)/20:3(5Z,8Z,11Z)) | HMDB0008079 | 1[25] |
| 375 | PC(18:1(11Z)/22:6(4Z,7Z,10Z,13Z,16Z,19Z)) | HMDB0008090 | 1[14] |
| 376 | PC(18:1(9Z)/22:5(4Z,7Z,10Z,13Z,16Z)) | HMDB0008121 | 1[1] |
| 377 | PC(18:2(9Z,12Z)/18:0) | HMDB0008135 | 1[28] |
| 378 | PC(18:2(9Z,12Z)/20:4(5Z,8Z,11Z,14Z)) | HMDB0008147 | 1[19] |
| 379 | PC(20:1(11Z)/18:4(6Z,9Z,12Z,15Z)) | HMDB0008306 | 1[19] |
| 380 | PC(20:4(5Z,8Z,11Z,14Z)/20:4(5Z,8Z,11Z, | HMDB0008443 | 1[14] |
| 775 | PPE(16:0/20:5) | / | 1[25] |
| 776 | PPE(18:1/18:1) | / | 1[25] |
| 777 | PPE(18:1/18:2) | / | 1[25] |
| 778 | PRISTIMEROL | / | 1[29] |
| 779 | Pro Lys Pro | / | 1[29] |
| 780 | Pro Pro | / | 1[29] |
| 781 | Propanoic acid, 2-hydroxy-3-[(4-hydroxy-1-naphthalenyl)oxy]- | / | 1[29] |
| 782 | Propionylglycine methyl ester | / | 1[29] |
| 783 | Prostaglandin | / | 1[28] |
| 784 | PS(18:1/0:0) | / | 1[56] |
| 785 | PS(34a:3) | / | 1[43] |
| 786 | PS(36a:4) | / | 1[43] |
| 787 | PYROGALLIN | / | 1[29] |
| 788 | Ritodrine glucuronide | / | 1[29] |
| 789 | S-3-Hydroxyisobutyric acid | / | 1[8] |
| 790 | Ser Asp Gly | / | 1[29] |
| 791 | SM (d18:1/18:0) | / | 1[19] |
| 792 | SM (d18:1/18:1) | / | 1[19] |
| 793 | SM (OH) C22:1 | / | 1[1] |
| 794 | SM (OH) C22:2 | / | 1[1] |
| 795 | SM (OH) C24:1 | / | 1[1] |

| | | | | | | | |
|---|---|---|---|---|---|---|---|
| | 14Z)) | | | | | | |
| 381 | PC(20:4(5Z,8Z,11Z,14Z)/22:6(4Z,7Z,10Z,13Z,16Z,19Z)) | HMDB0008452 | 1[14] | 796 | SM C16:0 | / | 1[1] |
| 382 | PC(22:0/18:4(6Z,9Z,12Z,15Z)) | HMDB0008536 | 1[1] | 797 | SM C18:1 | / | 1[1] |
| 383 | PE(14:1(9Z)/24:1(15Z)) | HMDB0008882 | 1[8] | 798 | SM C24:1 | / | 1[1] |
| 384 | PE(16:0/18:0) | HMDB0008925 | 1[14] | 799 | SM C26:1 | / | 1[1] |
| 385 | PE(16:0/20:5(5Z,8Z,11Z,14Z,17Z)) | HMDB0008939 | 1[25] | 800 | SM(d18:1/18:1) | / | 1[14] |
| 386 | PE(16:1(9Z)/20:5(5Z,8Z,11Z,14Z,17Z)) | HMDB0008972 | 1[25] | 801 | SM(d18:1/18:2) | / | 1[14] |
| 387 | PE(18:0/22:6(4Z,7Z,10Z,13Z,16Z,19Z)) | HMDB0009012 | 1[25] | 802 | SM(d18:1/20:1) | / | 1[8] |
| 388 | PE(18:1(11Z)/20:4(5Z,8Z,11Z,14Z)) | HMDB0009036 | 1[25] | 803 | SM(d18:1/20:1) | / | 1[8] |
| 389 | PE(18:1(11Z)/20:5(5Z,8Z,11Z,14Z,17Z)) | HMDB0009038 | 1[25] | 804 | SM(d18:1/24:2) | / | 1[8] |
| 390 | PE(18:1(11Z)/22:6(4Z,7Z,10Z,13Z,16Z,19Z)) | HMDB0009045 | 1[25] | 805 | SM(d18:1/24:2) | / | 1[8] |
| 391 | PE(18:2(9Z,12Z)/22:6(4Z,7Z,10Z,13Z,16Z,19Z)) | HMDB0009111 | 1[25] | 806 | SM(d18:1/25:0) | / | 1[8] |
| 392 | PE(22:5(4Z,7Z,10Z,13Z,16Z)/22:4(7Z,10Z,13Z,16Z)) | HMDB0009636 | 1[25] | 807 | SM(d18:1/25:0) | / | 1[8] |
| 393 | PE(22:6(4Z,7Z,10Z,13Z,16Z,19Z)/22:5(4Z,7Z,10Z,13Z,16Z)) | HMDB0009703 | 1[25] | 808 | SM(OH)C14:1 | / | 1[19] |
| 394 | PI(18:0/20:4(5Z,8Z,11Z,14Z)) | HMDB0009815 | 1[35] | 809 | S-Methylpenicillamine | / | 1[29] |
| 395 | LysoPC(16:1(9Z)) | HMDB0010383 | 1[13] | 810 | sphingosine-1-phosphate | / | 1[14] |
| 396 | LysoPC(20:3(5Z,8Z,11Z)) | HMDB0010393 | 1[56] | 811 | stra-1,3,5(10),7-tetraene-3,17alpha-diol | / | 1[56] |
| 397 | LysoPC(20:4(5Z,8Z,11Z,14Z)) | HMDB0010395 | 1[8] | 812 | SULBACTAM | / | 1[29] |
| 398 | TG(16:0/14:0/16:0) | HMDB0010411 | 1[8] | 813 | SULF(d18:1/18:0) | / | 1[14] |
| 399 | TG(16:0/14:0/16:1(9Z)) | HMDB0010412 | 1[8] | 814 | t8-iso-PGF2 | / | 1[41] |
| 400 | TG(16:1(9Z)/14:0/16:1(9Z)) | HMDB0010419 | 1[8] | 815 | TEGASEROD | / | 1[29] |

| | | | | | | | |
|---|---|---|---|---|---|---|---|
| 401 | 3-Oxododecanoic acid | HMDB0010727 | 1[29] | 816 | tetradecadienoylcarnitine | / | 1[15] |
| 402 | LysoPC(O-18:0) | HMDB0011149 | 1[14] | 817 | tetradecenoylcarnitine | / | 1[15] |
| 403 | PC(P-16:0/14:0) | HMDB0011203 | 1[19] | 818 | TG(51:1) | / | 1[8] |
| 404 | LysoPE(0:0/16:0) | HMDB0011473 | 1[25] | 819 | TG(O-50:0) | / | 1[8] |
| 405 | LysoPE(0:0/18:1(11Z)) | HMDB0011475 | 1[25] | 820 | THIODIGLYCOL | / | 1[29] |
| 406 | LysoPE(0:0/18:2(9Z,12Z)) | HMDB0011477 | 1[25] | 821 | Thr Gly | / | 1[29] |
| 407 | MG(16:0/0:0/0:0) | HMDB0011564 | 1[14] | 822 | Thr Ser Gln | / | 1[29] |
| 408 | gamma-Glutamylglycine | HMDB0011667 | 1[56] | 823 | thyrotropin releasing hormone | / | 1[29] |
| 409 | p-Cresol glucuronide | HMDB0011686 | 1[56] | 824 | Tripeptide | / | 1[26] |
| 410 | SM(d18:1/24:0) | HMDB0011697 | 1[35] | 825 | Trp Gly Phe | / | 1[29] |
| 411 | TG(15:0/16:0/20:2(11Z,14Z)) | HMDB0011700 | 1[8] | 826 | TUBAIC ACID | / | 1[29] |
| 412 | TG(15:0/16:0/20:3(8Z,11Z,14Z)) | HMDB0011701 | 1[8] | 827 | Tyr Tyr Thr | / | 1[29] |
| 413 | Methyldopa | HMDB0011754 | 1[8] | 828 | Val Gly | / | 1[29] |
| 414 | SM(d18:1/12:0) | HMDB0012096 | 1[14] | 829 | Val Ser Lys | / | 1[29] |
| 415 | SM(d18:1/14:0) | HMDB0012097 | 1[14] | 830 | VALERYL SALYCILATE | / | 1[29] |

[a]reference number are same as in Supplementary Table 2. Frequency indicates the reported frequency of the metabolite in previous studies included in this systematic review.

[b]aging-related metabolites

**Supplementary Table 5. Differential metabolites of mild cognitive impairment and their reported frequencies in previous studies**

| Number | Metabolite Name | HMDB ID | Frequency | Number | Metabolite Name | HMDB ID | Frequency[a] |
|---|---|---|---|---|---|---|---|
| 1 | L-Tryptophan | HMDB0000929 | 3[27,29,30] | 148 | 10-hydroxy-8E-Decene-2,4,6-triynoic acid | / | 1[29] |
| 2 | 5'-Methylthioadenosine | HMDB0001173 | 2[33,51] | 149 | 1a,1b-dihomo-PGJ2 | / | 1[29] |
| 3 | L-Valine | HMDB0000883 | 2[29,59] | 150 | VALERYL SALYCILATE | / | 1[29] |
| 4 | 5-Hydroxyindoleacetic acid | HMDB0000763 | 2[29,30] | 151 | VD 2656 | / | 1[29] |
| 5 | L-Methionine | HMDB0000696 | 2[29,30] | 152 | Visnagin | / | 1[29] |
| 6 | L-Arginine | HMDB0000517 | 2[29,51] | 153 | 1-Aminocyclohexanecarboxylic acid | / | 1[29] |
| 7 | 5-Hydroxy-L-tryptophan | HMDB0000472 | 2[29,30] | 154 | Tyr Pro | / | 1[29] |
| 8 | L-Phenylalanine | HMDB0000159 | 2[27,29] | 155 | 1-eicosanoyl-rac-glycerol | / | 1[29] |
| 9 | epsilon-Caprolactam | METPA0843 | 1[29] | 156 | 1-Hydroxyvitamin D3 3-D-glucopyranoside | / | 1[29] |
| 10 | Benzene-1,2,4-triol | METPA0328 | 1[29] | 157 | 1-Phenyl-2-(diethylamino)-1-propanol | / | 1[29] |
| 11 | (R)-Citronellal | HMDB0035820 | 1[29] | 158 | 2,5-Dimethoxycinnamic acid | / | 1[29] |
| 12 | Isoquinoline | HMDB0034244 | 1[29] | 159 | 20-oxo-heneicosanoic acid | / | 1[29] |
| 13 | 2-Amino-4-hydroxy-6-pteridinecarboxylic acid | HMDB0033136 | 1[29] | 160 | 2-Amino-3-methyl-1-butanol | / | 1[29] |
| 14 | Citropten | HMDB0032952 | 1[29] | 161 | Thr Ser Gln | / | 1[29] |
| 15 | Succinic anhydride | HMDB0032523 | 1[29] | 162 | 2-Hydroxy-3-(4-methoxyethylphenoxy)-propanoic acid | / | 1[29] |
| 16 | Acetamide | HMDB0031645 | 1[29] | 163 | 3-(a-Naphthoxy)lactic acid | / | 1[29] |
| 17 | 4-Hydroxybenzeneacetonitrile | HMDB0029757 | 1[29] | 164 | 3,6-octadecadiynoic acid | / | 1[29] |
| 18 | Glutamylglutamine | HMDB0028817 | 1[26] | 165 | TEGASEROD | / | 1[29] |
| 19 | Dimercaprol | HMDB0015677 | 1[29] | 166 | 3beta,4beta-Dihydroxy-5beta-cholan-24-oic Acid | / | 1[29] |
| 20 | Acenocoumarol | HMDB0015487 | 1[29] | 167 | 3-dodecynoic acid | / | 1[29] |
| 21 | Bepridil | HMDB0015374 | 1[29] | 168 | 4-dodecynoic acid | / | 1[29] |
| 22 | Penicillin G | HMDB0015186 | 1[29] | 169 | 4-hydroxy enanthoic acid | / | 1[29] |
| 23 | Netilmicin | HMDB0015090 | 1[29] | 170 | 4'-Hydroxyminoxidil | / | 1[29] |
| 24 | Aciclovir | HMDB0014925 | 1[29] | 171 | 4-undecynoic acid | / | 1[29] |
| 25 | Nalidixic Acid | HMDB0014917 | 1[29] | 172 | Trp Ala Ile | / | 1[29] |
| 26 | Azatadine | HMDB0014857 | 1[29] | 173 | 5,7-nonadienoic acid | / | 1[29] |
| 27 | Pirenzepine | HMDB0014808 | 1[29] | 174 | 5beta-Androstan-3alpha-ol-17-one sulfate | / | 1[29] |

| | | | | | | | |
|---|---|---|---|---|---|---|---|
| 28 | Ethosuximide | HMDB0014731 | 1[29] | 175 | TUBAIC ACID | / | 1[29] |
| 29 | Ethopropazine | HMDB0014536 | 1[29] | 176 | 5-NITRO-2-PHENYLPROPY LAMINOBENZOIC ACID [NPPB] | / | 1[29] |
| 30 | Tranexamic Acid | HMDB0014447 | 1[29] | 177 | 5-octadecylenic acid | / | 1[29] |
| 31 | Lidocaine | HMDB0014426 | 1[29] | 178 | 5-oxo-7-decynoic acid | / | 1[29] |
| 32 | Pyrimethamine | HMDB0014350 | 1[29] | 179 | 6-hydroxy-2-hexynoic acid | / | 1[29] |
| 33 | Lorazepam | HMDB0014332 | 1[29] | 180 | 7,12-Dioxo-5beta-cholan-24-oic Acid | / | 1[29] |
| 34 | N-Nonanoylglycine | HMDB0013279 | 1[33] | 181 | 7,8-Didehydroastaxanthin | / | 1[29] |
| 35 | 3-Dehydroquinate | HMDB0012710 | 1[29] | 182 | 8R-hydroxy-9Z-octadecenoic acid | / | 1[29] |
| 36 | SM(d18:1/14:0) | HMDB0012097 | 1[14] | 183 | 9,12-dioxo-dodecanoic acid | / | 1[29] |
| 37 | MG(18:0/0:0/0:0) | HMDB0011131 | 1[29] | 184 | 9-lauroleic acid | / | 1[29] |
| 38 | SM(d18:1/16:0) | HMDB0010169 | 1[14] | 185 | 9-pentadecen-1-ol | / | 1[29] |
| 39 | PE(16:0/18:0) | HMDB0008925 | 1[14] | 186 | Adriamycinone | / | 1[29] |
| 40 | PC(22:5(4Z,7Z,10Z,13Z,16Z)/14:1(9Z)) | HMDB0008657 | 1[20] | 187 | Ala Ala Asp | / | 1[29] |
| 41 | PC(20:0/18:3(6Z,9Z,12Z)) | HMDB0008271 | 1[20] | 188 | Ala Leu | / | 1[29] |
| 42 | PC(18:1(9Z)/22:5(4Z,7Z,10Z,13Z,16Z)) | HMDB0008121 | 1[20] | 189 | Ala Met Lys | / | 1[29] |
| 43 | PC(16:1(9Z)/22:6(4Z,7Z,10Z,13Z,16Z,19Z)) | HMDB0008023 | 1[14] | 190 | Ala Thr Pro | / | 1[29] |
| 44 | PC(16:0/20:3(5Z,8Z,11Z)) | HMDB0007980 | 1[14] | 191 | AMBELLINE | / | 1[29] |
| 45 | PC(16:0/18:2(9Z,12Z)) | HMDB0007973 | 1[14] | 192 | Anandamide (20:2, n-6) | / | 1[29] |
| 46 | PC(14:0/20:4(8Z,11Z,14Z,17Z)) | HMDB0007884 | 1[20] | 193 | Asn Gln Gln | / | 1[29] |
| 47 | Docosapentaenoic acid | HMDB0006528 | 1[29] | 194 | Asp Asn Glu | / | 1[29] |
| 48 | Loratadine | HMDB0005000 | 1[29] | 195 | BERGENIN | / | 1[29] |
| 49 | Guaifenesin | HMDB0004998 | 1[29] | 196 | bicyclo-PGE2 | / | 1[29] |
| 50 | Ceramide (d18:1/16:0) | HMDB0004949 | 1[14] | 197 | Bifemelane (M4) | / | 1[26] |
| 51 | 13,14-Dihydro-15-keto PGF2a | HMDB0004685 | 1[29] | 198 | C10:0-carnitine | / | 1[26] |
| 52 | Phytosphingosine | HMDB0004610 | 1[27] | 199 | C8-carnitine | / | 1[6] |
| 53 | Imidazolone | HMDB0004363 | 1[29] | 200 | cholesteryl ester 32:0 | / | 1[6] |
| 54 | Tryptophanol | HMDB0003447 | 1[29] | 201 | cholesteryl ester 32:4 | / | 1[6] |

| | | | |
|---|---|---|---|
| 55 | Diacetyl | HMDB0003407 | 1[29] |
| 56 | Acetoin | HMDB0003243 | 1[29] |
| 57 | Isoacitretin | HMDB0003039 | 1[29] |
| 58 | Testosterone sulfate | HMDB0002833 | 1[29] |
| 59 | Desmosterol | HMDB0002719 | 1[31] |
| 60 | 6-Hydroxynicotinic acid | HMDB0002658 | 1[29] |
| 61 | Sumiki's acid | HMDB0002432 | 1[29] |
| 62 | N-Acetylcadaverine | HMDB0002284 | 1[29] |
| 63 | 27-Nor-5b-cholestane-3a,7a,12a,24,25-pentol | HMDB0002126 | 1[29] |
| 64 | Oleamide | HMDB0002117 | 1[14] |
| 65 | N-Acetylputrescine | HMDB0002064 | 1[51] |
| 66 | 2-Pyrrolidinone | HMDB0002039 | 1[29] |
| 67 | D-threo-Isocitric acid | HMDB0001874 | 1[29] |
| 68 | Progesterone | HMDB0001830 | 1[27] |
| 69 | Asymmetric dimethylarginine | HMDB0001539 | 1[29] |
| 70 | Glucosamine | HMDB0001514 | 1[29] |
| 71 | Queuine | HMDB0001495 | 1[29] |
| 72 | Pyridoxamine | HMDB0001431 | 1[29] |
| 73 | Putrescine | HMDB0001414 | 1[51] |
| 74 | p-Aminobenzoic acid | HMDB0001392 | 1[29] |
| 75 | Diaminopimelic acid | HMDB0001370 | 1[29] |
| 76 | Purine | HMDB0001366 | 1[29] |
| 77 | SM(d18:1/18:0) | HMDB0001348 | 1[14] |
| 78 | N1-Acetylspermidine | HMDB0001276 | 1[26] |
| 79 | Spermidine | HMDB0001257 | 1[51] |
| 80 | Spermine | HMDB0001256 | 1[51] |
| 81 | N-Acetylarylamine | HMDB0001250 | 1[29] |
| 82 | (S)-Succinyldihydrolipoamide | HMDB0001177 | 1[29] |
| 83 | 4-Aminophenol | HMDB0001169 | 1[29] |
| 84 | Pyruvaldehyde | HMDB0001167 | 1[29] |
| 202 | cholesteryl ester 33:6 | / | 1[6] |
| 203 | cholesteryl ester 34:0 | / | 1[6] |
| 204 | cholesteryl ester 34:6 | / | 1[6] |
| 205 | cholesteryl ester 40:4 | / | 1[29] |
| 206 | cis-3-(6-Hydroxy-7-methoxy-5-benzofuranyl)acrylic acid | / | 1[29] |
| 207 | clavulone I | / | 1[29] |
| 208 | cyclohexylammonium | / | 1[54] |
| 209 | d18:1/16:0 | / | 1[54] |
| 210 | d18:1/24:0 | / | 1[54] |
| 211 | d18:1/24:1 | / | 1[29] |
| 212 | DALBERGIONE, 4-METHOXY-4'-HYDROXY- | / | 1[29] |
| 213 | DIALLYL SULFIDE | / | 1[29] |
| 214 | dihydro-alpha-ergocryptine | / | 1[29] |
| 215 | Dihydrolevobunolol | / | 1[29] |
| 216 | dodecanamide | / | 1[29] |
| 217 | EB 1213 | / | 1[29] |
| 218 | Embelin | / | 1[29] |
| 219 | EMODIC ACID | / | 1[29] |
| 220 | Estradiol valerate | / | 1[29] |
| 221 | Furafylline | / | 1[29] |
| 222 | GlcAbeta-Cer(d18:1/18:0) | / | 1[29] |
| 223 | Gln Pro Lys | / | 1[29] |
| 224 | Glu Ser | / | 1[29] |
| 225 | GPA(21:0/22:6(4Z,7Z,10Z,13Z,16Z,19Z)) | / | 1[29] |
| 226 | GPEtn(18:1(11Z)/18:1(9Z))[U] | / | 1[29] |
| 227 | GPEtn(20:4(5Z,8Z,11Z,14Z)/20:4(5Z,8Z,11Z,14Z))[U] | / | 1[29] |
| 228 | GPEtn(O-16:0/22:5(4Z,7Z,10Z,13Z,16Z)) | / | 1[29] |
| 229 | GPSer(16:0/18:1(11Z)) | / | 1[29] |
| 230 | Granisetron metabolite | / | 1[29] |
| 231 | Gualenate | / | 1[29] |

| | | | |
|---|---|---|---|
| 85 | 3-Aminopropionaldehyde | HMDB0001106 | 1[29] |
| 86 | 4-Aminobutyraldehyde | HMDB0001080 | 1[51] |
| 87 | Glyceraldehyde | HMDB0001051 | 1[29] |
| 88 | Biliverdin | HMDB0001008 | 1[29] |
| 89 | Trehalose | HMDB0000975 | 1[29] |
| 90 | Traumatic acid | HMDB0000933 | 1[29] |
| 91 | Stearoylcarnitine | HMDB0000848 | 1[14] |
| 92 | Phenylacetylglycine | HMDB0000821 | 1[14] |
| 93 | Indoleacrylic acid | HMDB0000734 | 1[29] |
| 94 | Hippuric acid | HMDB0000714 | 1[29] |
| 95 | L-Kynurenine | HMDB0000684 | 1[30] |
| 96 | Citraconic acid | HMDB0000634 | 1[29] |
| 97 | Cytosine | HMDB0000630 | 1[60] |
| 98 | Ethylmalonic acid | HMDB0000622 | 1[29] |
| 99 | 2-Furoic acid | HMDB0000617 | 1[29] |
| 100 | PC(16:0/16:0) | HMDB0000564 | 1[14] |
| 101 | Creatinine | HMDB0000562 | 1[29] |
| 102 | Caproic acid | HMDB0000535 | 1[33] |
| 103 | Aminoadipic acid | HMDB0000510 | 1[29] |
| 104 | 3-Methylhistidine | HMDB0000479 | 1[29] |
| 105 | 3,4-Dihydroxyhydrocinnamic acid | HMDB0000423 | 1[29] |
| 106 | 3-Hydroxydodecanedioic acid | HMDB0000413 | 1[29] |
| 107 | 2-Methyl-3-ketovaleric acid | HMDB0000408 | 1[29] |
| 108 | 2-Hydroxy-3-methylbutyric acid | HMDB0000407 | 1[29] |
| 109 | 3-Hydroxybutyric acid | HMDB0000357 | 1[59] |
| 110 | 2-Methylbutyrylglycine | HMDB0000339 | 1[29] |
| 111 | Urocanic acid | HMDB0000301 | 1[29] |
| 112 | Uric acid | HMDB0000289 | 1[30] |
| 113 | Sphingosine 1-phosphate | HMDB0000277 | 1[14] |
| 114 | Sphinganine | HMDB0000269 | 1[27] |
| 115 | Pyroglutamic acid | HMDB0000267 | 1[29] |
| 116 | Taurine | HMDB0000251 | 1[29] |
| 117 | Tetrahydrofuran | HMDB0000246 | 1[29] |
| 232 | Gummiferol | / | 1[14] |
| 233 | Hex-CER(d18:1/16:0) | / | 1[14] |
| 234 | Hex-CER(d18:1/18:0) | / | 1[29] |
| 235 | Ile Ser Lys | / | 1[14] |
| 236 | inoleyl-carnitine | / | 1[29] |
| 237 | KARANJIN | / | 1[14] |
| 238 | Lac-CER(d18:1/14:0) | / | 1[14] |
| 239 | Lac-CER(d18:1/16:0) | / | 1[14] |
| 240 | Lac-CER(d18:1/16:1) | / | 1[29] |
| 241 | L-Alanine n-butyl ester | / | 1[29] |
| 242 | Leu Glu Gln | / | 1[29] |
| 243 | Leu Leu Ala | / | 1[29] |
| 244 | L-Glutamic acid dibutyl ester | / | 1[29] |
| 245 | Loroxanthin ester/ Loroxanthin dodecenoate | / | 1[27] |
| 246 | LPC C 18:0 | / | 1[27] |
| 247 | LPC C 18:1 | / | 1[27] |
| 248 | LPC C 18:2 | / | 1[27] |
| 249 | LPC C 20:4 | / | 1[20] |
| 250 | lysoPC a C18:2 | / | 1[29] |
| 251 | Mebeverine | / | 1[29] |
| 252 | Met Trp Gln | / | 1[29] |
| 253 | Methoxsalen Metabolite | / | 1[29] |
| 254 | METHYL 7-DESHYDROXYPYROGALLIN-4-CARBOXYLATE | / | 1[33] |
| 255 | methyl-salsolinol | / | 1[51] |
| 256 | N1 or N8-acetyl-spermidine | / | 1[51] |
| 257 | N1,N12-diacetlyspermine | / | 1[29] |
| 258 | N4-Acetylsulfapyridine | / | 1[29] |
| 259 | SM(OH) C14:1 | / | 1[14] |
| 260 | N-arachidonoyl D-serine | / | 1[29] |
| 261 | N-Didesethylquinagolide | / | 1[29] |
| 262 | N-methyl-Gabapentin | / | 1[14] |
| 263 | oleylcarnitine | / | 1[29] |
| 264 | Ophiobolin A | / | 1[20] |

| | | | |
|---|---|---|---|
| 118 | Pyruvic acid | HMDB0000243 | 1[29] |
| 119 | Ornithine | HMDB0000214 | 1[51] |
| 120 | Oxoglutaric acid | HMDB0000208 | 1[29] |
| 121 | Indoleacetic acid | HMDB0000197 | 1[30] |
| 122 | L-Lactic acid | HMDB0000190 | 1[59] |
| 123 | L-Asparagine | HMDB0000168 | 1[26] |
| 124 | Hypoxanthine | HMDB0000157 | 1[30] |
| 125 | Fumaric acid | HMDB0000134 | 1[29] |
| 126 | D-Glucose | HMDB0000122 | 1[59] |
| 127 | Gamma-Aminobutyric acid | HMDB0000112 | 1[51] |
| 128 | Choline | HMDB0000097 | 1[26] |
| 129 | Dimethylglycine | HMDB0000092 | 1[29] |
| 130 | Cholesterol | HMDB0000067 | 1[6] |
| 131 | Creatine | HMDB0000064 | 1[51] |
| 132 | L-Carnitine | HMDB0000062 | 1[26] |
| 133 | Acetoacetic acid | HMDB0000060 | 1[29] |
| 134 | Bilirubin | HMDB0000054 | 1[29] |
| 135 | Acetic acid | HMDB0000042 | 1[59] |
| 136 | Biotin | HMDB0000030 | 1[29] |
| 137 | 4-Pyridoxic acid | HMDB0000017 | 1[29] |
| 138 | (17Z)-1alpha,25-dihydroxy-26,27-dimethyl-17,20,22,22,23,23-hexadehydrovitamin D3 / (17Z)-1alpha,25-d | / | 1[29] |
| 139 | (22E,24E)-1alpha,25-dihydroxy-22,23,24,24a-tetradehydro-24a-homovitamin D3 / (22E,24E)-1alpha,25-dih | / | 1[29] |
| 140 | (22R)-1alpha,22,25-trihydroxy-23,24-tetradehydro-24a,24b-dihomo-20-epivitamin D3 / (22R)-1alpha,22,2 | / | 1[29] |
| 141 | (22R)-1alpha,22,25 | / | 1[29] |

| | | | |
|---|---|---|---|
| 265 | PC aa C40:5 | / | 1[7] |
| 266 | PC aaC32:0 | / | 1[7] |
| 267 | PC aeC32:2 | / | 1[7] |
| 268 | PC aeC34:1 | / | 1[43] |
| 269 | PC(32a:0) | / | 1[43] |
| 270 | PC(38a:5) | / | 1[43] |
| 271 | PC(38a:6) | / | 1[26] |
| 272 | Phe Phe | / | 1[29] |
| 273 | Phe Val Val | / | 1[14] |
| 274 | PPC(16:0/18:2) | / | 1[29] |
| 275 | Pro Pro | / | 1[29] |
| 276 | Propionylglycine methyl ester | / | 1[43] |
| 277 | PS(38a:6) | / | 1[29] |
| 278 | PURPUROGALLIN | / | 1[29] |
| 279 | PYROGALLIN | / | 1[29] |
| 280 | QUERCETIN TETRAMETHYL (5,7,3',4') ETHER | / | 1[29] |
| 281 | Rimiterol | / | 1[29] |
| 282 | Ritodrine glucuronide | / | 1[29] |
| 283 | S-(p-Azidophenacyl)glutathione | / | 1[14] |
| 284 | SM(d18:1/18:1) | / | 1[7] |
| 285 | 1alpha,25-dihydroxy-11beta-phenylvitamin D3 / 1alpha,25-dihydroxy-11beta-phenylcholecalciferol | / | 1[29] |
| 286 | 1alpha,25-dihydroxy-22-oxavitamin D3 3-hemiglutarate/ 1alpha,25-dihydroxy-22-oxacholecalciferol 3-he | / | 1[29] |
| 287 | 1alpha,25-dihydroxy-3-deoxy-3-thiavitamin D3 / 1alpha,25-dihydroxy-3-deoxy-3-thiacholecalciferol | / | 1[29] |
| 288 | 1beta,25-dihydroxy-2beta-(3-h | / | 1[29] |

| | | | | | | | |
|---|---|---|---|---|---|---|---|
| | -trihydroxy-26,27-dimethyl-23,24-tetradehydro-24a-homo-20-epivitamin D3 / (22R)-1a | | | | ydroxypropoxy)vitamin D3 / 1beta,25-dihydroxy-2beta-(3-hydroxypropoxy)c | | |
| 142 | (22S)-1alpha,22,25-trihydroxy-23,24-tetradehydro-24a,24b-dihomo-20-epivitamin D3 / (22S)-1alpha,22,2 | / | 1[29] | 289 | 5-(4-hydroxy-2,5-dimethylphenoxy)-2,2-dimethyl-Pentanoic acid (Gemfibrozil M1) | / | 1[29] |
| 143 | (23R)-1alpha,23,25-trihydroxy-24-oxovitamin D3 / (23R)-1alpha,23,25-trihydroxy-24-oxocholecalciferol | / | 1[29] | 290 | 5H-Oxireno[4,5]furo[3,2-g][1]benzopyran-5-one, 1a,8b-dihydro-3-methoxy- | / | 1[29] |
| 144 | (23R,25R)-1alpha,25-dihydroxyvitamin D3 26,23-lactone / (23R,25R)-1alpha,25-dihydroxycholecalciferol | / | 1[29] | 291 | 3alpha,6alpha,12alpha-Trihydroxy-7-oxo-5beta-cholan-24-oic Acid | / | 1[29] |
| 145 | (24S)-1alpha,24-dihydroxy-22-oxa-20-epivitamin D3 / (24S)-1alpha,24-dihydroxy-22-oxa-20-epicholecalc | / | 1[29] | 292 | N'-5Z,8Z,11Z,14Z-eicosatetraenoyl-N''-diethyl-ethylenediamine | / | 1[29] |
| 146 | (24S)-1alpha,24-dihydroxy-22-oxavitamin D3 / (24S)-1alpha,24-dihydroxy-22-oxacholecalciferol | / | 1[29] | 293 | 2H-1-Benzopyran-6-acetic acid, 7-hydroxy-8-methoxy-2-oxo- | / | 1[29] |
| 147 | 1,3-DIPROPYL-8-CYCLOPENTYLXANTHINE [DPCPX] | / | 1[29] | | | | |

[a]reference number are same as in Supplementary Table 2. Frequency indicates the reported frequency of the metabolite in previous studies included in this systematic review.

**Supplementary Table 6. Differential metabolites between Alzheimer's disease and mild cognitive impairment and their reported frequencies in previous studies**

| Number | Metabolite Name | HMDB ID | Frequency | Number | Metabolite Name | HMDB ID | Frequency[a] |
|---|---|---|---|---|---|---|---|
| 1 | L-Histidine | HMDB0000177 | 2[14,26] | 61 | SULF(d18:1/18:0) | / | 1[14] |
| 2 | L-Tryptophan | HMDB0000929 | 2[27,29] | 62 | 1-Aminocyclohexane carboxylic acid | / | 1[29] |
| 3 | Creatine | HMDB0000064 | 1[34] | 63 | 1-Hexadecylamine | / | 1[29] |
| 4 | 2-Hydroxybutyric acid | HMDB0000008 | 1[15] | 64 | 20alpha-Dihydropred nisolone | / | 1[29] |
| 5 | 4-Pyridoxic acid | HMDB0000017 | 1[29] | 65 | 2-oxo-4-hydroxy-hex anoic acid | / | 1[29] |
| 6 | Pipecolic acid | HMDB0000070 | 1[33] | 66 | 3,6-octadecadiynoic acid | / | 1[29] |
| 7 | Choline | HMDB0000097 | 1[63] | 67 | 3E-undecenoic acid | / | 1[29] |
| 8 | Glycolic acid | HMDB0000115 | 1[29] | 68 | 4-Hydroxytacrine | / | 1[29] |
| 9 | L-Tyrosine | HMDB0000158 | 1[29] | 69 | 5,7-nonadienoic acid | / | 1[29] |
| 10 | L-Phenylalanine | HMDB0000159 | 1[29] | 70 | Trp Gly Phe | / | 1[29] |
| 11 | L-Proline | HMDB0000162 | 1[29] | 71 | 5-hydroxmethylcytos ine | / | 1[60] |
| 12 | L-Acetylcarnitine | HMDB0000201 | 1[15] | 72 | 5-tert-Butyl-4-hydro xymethylfuran-2-car boxylic acid | / | 1[29] |
| 13 | Phenol | HMDB0000228 | 1[29] | 73 | Ala Leu | / | 1[29] |
| 14 | Taurine | HMDB0000251 | 1[33] | 74 | Allocortol | / | 1[29] |
| 15 | Sphinganine | HMDB0000269 | 1[27] | 75 | Arg Asn Gln | / | 1[29] |
| 16 | Urea | HMDB0000294 | 1[29] | 76 | Asn Gly Ser | / | 1[29] |
| 17 | Uracil | HMDB0000300 | 1[33] | 77 | BUFEXAMAC | / | 1[29] |
| 18 | 2-Methylbutyrylgly cine | HMDB0000339 | 1[29] | 78 | C10:1-carnitine | / | 1[26] |
| 19 | 3-Methylindole | HMDB0000466 | 1[29] | 79 | Carbaryl | / | 1[29] |
| 20 | 5-Hydroxy-L-trypto phan | HMDB0000472 | 1[30] | 80 | Chloramphenicol alcohol | / | 1[29] |
| 21 | 3-Methylhistidine | HMDB0000479 | 1[29] | 81 | clavulone I | / | 1[29] |
| 22 | L-Arginine | HMDB0000517 | 1[63] | 82 | Gly His | / | 1[29] |
| 23 | Cytosine | HMDB0000630 | 1[60] | 83 | decenoylcarnitine | / | 1[15] |
| 24 | Deoxycholic acid glycine conjugate | HMDB0000631 | 1[2] | 84 | Diglycolic acid | / | 1[29] |
| 25 | Chenodeoxycholic acid glycine conjugate | HMDB0000637 | 1[2] | 85 | dodecenoylcarnitine | / | 1[15] |
| 26 | Decanoylcarnitine | HMDB0000651 | 1[15] | 86 | dopamine-quinone | / | 1[33] |
| 27 | L-Methionine | HMDB0000696 | 1[26] | 87 | free carnitine | / | 1[15] |
| 28 | Lithocholic acid | HMDB0000698 | 1[2] | 88 | DALBERGIONE, | / | 1[29] |

| | | | | | | | |
|---|---|---|---|---|---|---|---|
| | glycine conjugate | | | | 4-METHOXY-4'-HYDROXY-hydroxyphophinyl-piruvate | | |
| 29 | Indoleacrylic acid | HMDB0000734 | 1[29] | 89 | | / | 1[33] |
| 30 | Pregnenolone sulfate | HMDB0000774 | 1[14] | 90 | LPE (16:0) | / | 1[14] |
| 31 | L-Octanoylcarnitine | HMDB0000791 | 1[15] | 91 | L-Glutamic acid dibutyl ester | / | 1[29] |
| 32 | L-Valine | HMDB0000883 | 1[63] | 92 | LPC (16:1) | / | 1[14] |
| 33 | Traumatic acid | HMDB0000933 | 1[29] | 93 | LPC (22:6) | / | 1[14] |
| 34 | Trehalose | HMDB0000975 | 1[29] | 94 | LPC C 16:0 | / | 1[27] |
| 35 | (S)-Succinyldihydrolipoamide | HMDB0001177 | 1[29] | 95 | LPC C 18：0 | / | 1[27] |
| 36 | Pyridoxamine | HMDB0001431 | 1[29] | 96 | LPC C 18：1 | / | 1[27] |
| 37 | Asymmetric dimethylarginine | HMDB0001539 | 1[29] | 97 | LPC C 18:2 | / | 1[27] |
| 38 | Caffeine | HMDB0001847 | 1[29] | 98 | LPC C 20:4 | / | 1[27] |
| 39 | 27-Nor-5b-cholestane-3a,7a,12a,24,25-pentol | HMDB0002126 | 1[29] | 99 | L-gamma-Cyano-gamma-aminobutyric acid | / | 1[29] |
| 40 | Dodecanoylcarnitine | HMDB0002250 | 1[15] | 100 | LPE (18:2) | / | 1[14] |
| 41 | Terephthalic acid | HMDB0002428 | 1[29] | 101 | Methoxsalen Metabolite | / | 1[29] |
| 42 | 5-Methylcytosine | HMDB0002894 | 1[60] | 102 | Propionylglycine methyl ester | / | 1[29] |
| 43 | Isoacitretin | HMDB0003039 | 1[29] | 103 | N,N-Didemethylchlorpromazine | / | 1[29] |
| 44 | 5-Methoxyindoleacetate | HMDB0004096 | 1[29] | 104 | Ne-Methyl-L-lysine | / | 1[29] |
| 45 | L-Urobilin | HMDB0004159 | 1[29] | 105 | N-methyl-Gabapentin | / | 1[29] |
| 46 | Guaifenesin | HMDB0004998 | 1[29] | 106 | oleylcarnitine | / | 1[15] |
| 47 | MG(18:0/0:0/0:0) | HMDB0011131 | 1[14] | 107 | Phenyl sulfate | / | 1[29] |
| 48 | LysoPC(O-18:0) | HMDB0011149 | 1[14] | 108 | Phenytoin-N-glucuronide | / | 1[29] |
| 49 | MG(16:0/0:0/0:0) | HMDB0011564 | 1[14] | 109 | PPE(16:0/22:6) | / | 1[14] |
| 50 | Methylphenidate | HMDB0014566 | 1[29] | 110 | PPE(18:1/20:4) | / | 1[14] |
| 51 | Isoniazid | HMDB0015086 | 1[29] | 111 | Pro Lys Pro | / | 1[29] |
| 52 | 4-Hydroxybenzeneacetonitrile | HMDB0029757 | 1[29] | 112 | SULBACTAM | / | 1[29] |
| 53 | Clofenotane | HMDB0032127 | 1[29] | 113 | METHYL 7-DESHYDROXYPYROGALLIN-4-CA | / | 1[29] |

| | | | | | | | |
|---|---|---|---|---|---|---|---|
| | | | | | RBOXYLATE | | |
| 54 | Succinic anhydride | HMDB0032523 | 1[29] | 114 | tetradecadienoylcarnitine | / | 1[15] |
| 55 | Tyrosyl-Serine | HMDB0029114 | 1[33] | 115 | tetradecenoylcarnitine | / | 1[15] |
| 56 | Anthracene | METPA1122 | 1[29] | 116 | tripeptide | / | 1[63] |
| 57 | 1,3-Glyceryl dinitrate | / | 1[29] | 117 | 13E,17-octadecadienoic acid | / | 1[29] |
| 58 | 10-hydroxy-11-dodecenoic acid | / | 1[29] | 118 | Tyr Pro | / | 1[29] |
| 59 | 5-[2-(hydroxymethyl)-5-methylphenoxy]-2,2-dimethyl-Pentanoic acid (Gemfibrozil M4) | / | 1[29] | 119 | 1alpha,25-dihydroxy-2beta-(5-hydroxypentoxy)vitamin D3 / 1alpha,25-dihydroxy-2beta-(5-hydroxypentoxy | / | 1[29] |
| 60 | (22R)-1alpha,22,25-trihydroxy-26,27-dimethyl-23,24-tetradehydro-24a-homo-20-epivitamin D3 / (22R)-1a | / | 1[29] | 120 | 1alpha,25-dihydroxy-3-deoxy-3-thiavitamin D3 / 1alpha,25-dihydroxy-3-deoxy-3-thiacholecalciferol | / | 1[29] |

[a]reference number are same as in Supplementary Table 2. Frequency indicates the reported frequency of the metabolite in previous studies included in this systematic review.

**Supplementary Table 7. Metabolites related with Alzheimer's disease replicated in previous prospective studies**

| Metabolite Name | HMDB ID | Frequency[a] |
|---|---|---|
| L-Glutamine | HMDB0000641 | 1[58] |
| Dihydroxyacetone | HMDB0001882 | 1[58] |
| Palmitic acid | HMDB0000220 | 1[64] |
| Linoleic acid | HMDB0000673 | 1[64] |
| Myristic acid | HMDB0000806 | 1[64] |
| Stearic acid | HMDB0000827 | 1[64] |
| Oleic acid | HMDB0000207 | 1[64] |
| S-HDL-free cholesterol | / | 1[58] |
| M-HDL-phospholipids | / | 1[58] |

[a]reference number are same as in Supplementary Table 2. Frequency indicates the reported frequency of the metabolite in previous studies included in this systematic review.

**Supplementary Table 8. Metabolites related with the conversion from mild cognitive impairment to Alzheimer's disease in previous prospective studies**

| Number | Metabolite Name | HMDB ID | Frequency[a] |
|---|---|---|---|
| 1 | Creatine | HMDB0000064 | 1[51] |
| 2 | L-Histidine | HMDB0000177 | 1[33] |
| 3 | L-Lactic acid | HMDB0000190 | 1[35] |
| 4 | Pyruvic acid | HMDB0000243 | 1[35] |
| 5 | Xanthine | HMDB0000292 | 1[33] |
| 6 | 2,4-Dihydroxybutanoic acid | HMDB0000360 | 1[35] |
| 7 | L-Arginine | HMDB0000517 | 1[51] |
| 8 | 4-Aminobutyraldehyde | HMDB0001080 | 1[51] |
| 9 | 5'-Methylthioadenosine | HMDB0001173 | 1[51] |
| 10 | Spermine | HMDB0001256 | 1[51] |
| 11 | Spermidine | HMDB0001257 | 1[51] |
| 12 | Putrescine | HMDB0001414 | 1[51] |
| 13 | D-Ribose 5-phosphate | HMDB0001548 | 1[35] |
| 14 | PC(o-22:0/18:3(6Z,9Z,12Z)) | HMDB0013445 | 1[65] |
| 15 | PC(o-22:0/20:4(8Z,11Z,14Z,17Z)) | HMDB0013448 | 1[65] |
| 16 | PC(o-2:1(13Z)/22:3(10Z,13Z,16Z)) | HMDB0013453 | 1[65] |
| 17 | PC(o-18:0/18:2(9Z,12Z)) | HMDB0013418 | 1[65] |
| 18 | PC(16:0/20:5(5Z,8Z,11Z,14Z,17Z)) | HMDB0007984 | 1[35] |
| 19 | lysoPC (16:0) | / | 1[35] |
| 20 | PC (18:0/20:4) | / | 1[35] |
| 21 | PC (O-18:1/16:0) | / | 1[35] |
| 22 | N1 or N8-acetyl-spermidine | / | 1[51] |
| 23 | SM (OH) C14:1 | / | 1[65] |
| 24 | SM C16:0 | / | 1[65] |
| 25 | SM C20:2 | / | 1[65] |
| 26 | PC (16:0/16:0) | / | 1[35] |

[a]reference number are same as in Supplementary Table 2. Frequency indicates the reported frequency of the metabolite in previous studies included in this systematic review.

**Supplementary Table 9. Pathways altered in mild cognitive impairment and Alzheimer's disease identified by direct extraction and pathway enrichment analysis**

| Groups/Pathway Name | Biosamples | Software | Source[a] | Frequency[b] |
|---|---|---|---|---|
| **AD VS CN** | | | | |
| Alanine, aspartate and glutamate metabolism | brain, CSF, serum, urine | Mummichog, ORA, MetPA | enrichment, article | 3 |
| TCA cycle | brain, CSF, serum, plasma | Mummichog, MetaCore, MetPA | enrichment, article | 3 |
| Purine metabolism | brain, CSF, serum, plasma, urine, saliva | Metaboanalyst, ORA, Mummichog | enrichment, article | 3 |
| Arginine and proline metabolism | brain, CSF, serum, saliva, plasma, urine | Mummichog, ORA | enrichment, article | 2 |
| Cysteine and methionine metabolism | brain, CSF, serum, plasma, urine | Mummichog, ORA | enrichment, article | 2 |
| Glycine, serine and threonine metabolism | brain, CSF, serum, plasma, urine | Mummichog, ORA | enrichment, article | 2 |
| Pantothenate and CoA biosynthesis | brain, CSF, serum, plasma | Mummichog, ORA | enrichment, article | 2 |
| tryptophan metabolism | plasma, serum | MetaCore, MetPA | article | 2 |
| Aminoacyl-tRNA biosynthesis | brain, CSF, serum, plasma, urine | Mummichog | enrichment | 1 |
| Nitrogen metabolism | brain, CSF, serum, plasma, urine | Mummichog | enrichment | 1 |
| Glutathione metabolism | brain, CSF, serum, plasma, urine, saliva | Mummichog | enrichment | 1 |
| beta-Alanine metabolism | brain, CSF, serum, plasma, urine | Mummichog | enrichment | 1 |
| Valine, leucine and isoleucine biosynthesis | brain, CSF, serum, plasma | Mummichog | enrichment | 1 |
| Sphingolipid metabolism | brain, CSF, serum, plasma, saliva | Mummichog | enrichment | 1 |
| Biotin metabolism | brain, CSF, plasma, urine | Mummichog | enrichment | 1 |
| Butanoate metabolism | brain, CSF, plasma | Mummichog | enrichment | 1 |
| Propanoate metabolism | brain, CSF, serum, plasma | Mummichog | enrichment | 1 |
| Cholesterol and sphingolipids transport | plasma, CSF | MetaCore | article | 1 |
| Vitamin d2 metabolism | plasma | MetaCore | article | 1 |
| Polyamine metabolism | plasma | MetaCore | article | 1 |
| Urea cycle | plasma, CSF | MetaCore | article | 1 |
| Intracellular cholesterol transport | plasma, CSF | MetaCore | article | 1 |
| (L)-Arginine metabolism | plasma, CSF | MetaCore | article | 1 |
| cortisone biosynthesis and metabolism | plasma, CSF | MetaCore | article | 1 |
| lipid metabolism | plasma | MetaCore | article | 1 |
| FXR-regulated cholesterol and bile acid | plasma | MetaCore | article | 1 |

| | | | | |
|---|---|---|---|---|
| transport | | | | |
| Lysine metabolism | plasma | MetaCore | article | 1 |
| Bile acid metabolism | plasma, CSF | MetaCore | article | 1 |
| Regulation of CFTR gating | plasma, CSF | MetaCore | article | 1 |
| Role of VDR in regulation of genes involved in osteoporosis | plasma | MetaCore | article | 1 |
| Vitamin D3 metabolic C-23 and C-24 pathways | plasma | MetaCore | article | 1 |
| Triacylglycerol biosynthesis in obesity and diabetes mellitus, type II | plasma | MetaCore | article | 1 |
| Mechanism of action of DGaT1 in obesity and diabetes mellitus, type II | plasma | MetaCore | article | 1 |
| Aminoacyl-tRNA biosynthesis in mitochondria | plasma | MetaCore | article | 1 |
| Triacylglycerol metabolism p.2 | plasma | MetaCore | article | 1 |
| Muscle contraction_nNOS signaling in skeletal muscles | plasma | MetaCore | article | 1 |
| Aminoacyl-tRNA biosynthesis in cytoplasm | plasma | MetaCore | article | 1 |
| Regulation of lipid MB FXR-dependent negative-feedback regulation of bile acid concentration | plasma | MetaCore | article | 1 |
| Cortisol biosynthesis from cholesterol | plasma | MetaCore | article | 1 |
| Development_Activation of astroglia cell proliferation by ACM3 | plasma | MetaCore | article | 1 |
| Fatty Acid Omega Oxidation | plasma | MetaCore | article | 1 |
| Prostaglandin 2 biosynthesis and metabolism | CSF | MetaCore | article | 1 |
| Aspartate and aspargine metabolism | CSF | MetaCore | article | 1 |
| Nicotine metabolism in liver | CSF | MetaCore | article | 1 |
| Cholesterol and sphingolipids transport/transport from Golgi | CSF | MetaCore | article | 1 |
| Glycolysis and gluconeogenesis | CSF | MetaCore | article | 1 |
| FXR-regulated cholesterol and bile acid cellular transport | CSF | MetaCore | article | 1 |
| Saturated fatty acids metabolism | CSF | MetaCore | article | 1 |
| Cortisol BS from cholesterol | CSF | MetaCore | article | 1 |
| arginineand proline metabolism | serum | MetPA | article | 1 |
| histidine metabolism | serum | MetPA | article | 1 |
| Pyruvate metabolism | serum | MetPA | article | 1 |
| phenylalanine metabolism | serum | MetPA | article | 1 |
| **MCI VS CN** | | | | |
| lysine metabolism | plasma | MetaCore | article | 2 |
| tryptophan metabolism | plasma | MetaCore | article | 2 |
| Polyamine metabolism | plasma | MetaCore | article | 2 |
| Urea cycle | CSF, plasma | MetaCore | article | 2 |
| Arginine and proline metabolism | CSF, plasma | Mummichog | enrichment | 1 |
| Butanoate metabolism | CSF, plasma, serum | Mummichog | enrichment | 1 |

| | | | | |
|---|---|---|---|---|
| Alanine, aspartate and glutamate metabolism | CSF, plasma, serum | Mummichog | enrichment | 1 |
| beta-Alanine metabolism | CSF, plasma | Mummichog | enrichment | 1 |
| Glycine, serine and threonine metabolism | CSF, plasma, serum | Mummichog | enrichment | 1 |
| Sphingolipid metabolism | plasma, serum | Mummichog | enrichment | 1 |
| Glutathione metabolism | CSF, plasma | Mummichog | enrichment | 1 |
| Synthesis and degradation of ketone bodies | CSF, plasma, serum | Mummichog | enrichment | 1 |
| Glycolysis or Gluconeogenesis | CSF, serum | Mummichog | enrichment | 1 |
| Phenylalanine metabolism | CSF, plasma, serum | Mummichog | enrichment | 1 |
| D-Arginine and D-ornithine metabolism | plasma | Mummichog | enrichment | 1 |
| Vitamin B6 metabolism | CSF, plasma | Mummichog | enrichment | 1 |
| Pyruvate metabolism | CSF, serum | Mummichog | enrichment | 1 |
| TCA cycle | plasma, CSF | MetaCore | article | 1 |
| saturated fatty acid metabolism | plasma | MetaCore | article | 1 |
| prostaglandin 2 biosynthesis and metabolism | plasma, CSF | MetaCore | article | 1 |
| aminoacyl-tRNA biosynthesis in cytoplasm | plasma | MetaCore | article | 1 |
| neurophysiological process_ delta-type opioid receptor in the nervous system | plasma | MetaCore | article | 1 |
| melatonin signaling | plasma | MetaCore | article | 1 |
| Nicotine MB in liver | CSF | MetaCore | article | 1 |
| Cortisone biosynthesis and metabolism | CSF | MetaCore | article | 1 |
| Role of Diethylhexyl Phthalate and Tributyltin in fat differentiation | CSF | MetaCore | article | 1 |
| Phospholipid metabolism | plasma | MetaCore | article | 1 |
| CHOL & Sphingolipid transport | plasma | MetaCore | article | 1 |
| Sat fatty acid biosynthesis | plasma | MetaCore | article | 1 |
| Regulation of lipid metabolism | plasma | MetaCore | article | 1 |
| Glycolysis & GNG p.2 | plasma | MetaCore | article | 1 |
| Transport of IC CHO | plasma | MetaCore | article | 1 |
| Niacin-HDL metabolism | plasma | MetaCore | article | 1 |
| Plasmalogen biosynthesis | plasma | MetaCore | article | 1 |
| UMP biosynthesis | plasma | MetaCore | article | 1 |
| GABA biosynthesis& metabolism | plasma | MetaCore | article | 1 |
| PG 2 biosynthesis & metabolism | plasma | MetaCore | article | 1 |
| Glycolysis GNG(short map) | plasma | MetaCore | article | 1 |
| L-Arginine metabolism | plasma | MetaCore | article | 1 |
| **AD VS MCI** | | | | |
| Nitrogen metabolism | CSF, serum, plasma | Mummichog, MetaCore | enrichment, article | 2 |
| Aminoacyl-tRNA biosynthesis | CSF, serum, plasma | Mummichog | enrichment | 1 |
| Phenylalanine, tyrosine and tryptophan biosynthesis | CSF, plasma | Mummichog | enrichment | 1 |
| Pyrimidine metabolism | CSF, plasma, DNA of brain tissue | Mummichog | enrichment | 1 |
| Lysine metabolism | plasma | MetaCore | article | 1 |

| | | | | |
|---|---|---|---|---|
| Cholesterol metabolism | plasma | MetaCore | article | 1 |
| Arginine metabolism | plasma | MetaCore | article | 1 |
| Vitamin D3 metabolic C-23 and C-24 pathways | plasma | MetaCore | article | 1 |
| Aminoacyl-tRNA biosynthesis in cytoplasm | plasma | MetaCore | article | 1 |
| Polyamine metabolism | plasma | MetaCore | article | 1 |
| Urea cycle | plasma | MetaCore | article | 1 |
| Bile Acid Biosynthesis | plasma | MetaCore | article | 1 |
| Aminoacyl-tRNA biosynthesis in mitochondrion | plasma | MetaCore | article | 1 |
| Histidine-glutamate-glutamine metabolism | plasma | MetaCore | article | 1 |
| Neurophysiological process_GABAergic neurotransmission | plasma | MetaCore | article | 1 |
| tryptophan metabolism | plasma | MetaCore | article | 1 |
| Proline metabolism | plasma | MetaCore | article | 1 |
| Saturated fatty acid biosynthesis | plasma | MetaCore | article | 1 |
| Catecholamine metabolism | plasma | MetaCore | article | 1 |
| Muscle contraction_nNOS Signaling in Skeletal Muscle | plasma | MetaCore | article | 1 |
| Acetylcholine biosynthesis and metabolism | plasma | MetaCore | article | 1 |
| Development Activation of astroglial cells proliferation by ACM3 | plasma | MetaCore | article | 1 |
| Fatty Acid Omega Oxidation | plasma | MetaCore | article | 1 |
| Nicotine signaling in GABAergic neurons | plasma | MetaCore | article | 1 |
| Neurophysiological process_ ACM1 and ACM2 in neuronal… | plasma | MetaCore | article | 1 |
| Serotonin modulation of dopamine release in nicotine addiction | plasma | MetaCore | article | 1 |
| Transport ACM3 in salivary glands | plasma | MetaCore | article | 1 |
| Development_ACM2 and ACM4 activation of ERK | plasma | MetaCore | article | 1 |
| Prostaglandin 2 biosynthesis and metabolism | CSF | MetaCore | article | 1 |
| Cortisone biosynthesis and metabolism | CSF | MetaCore | article | 1 |
| Regulation of CFTR gating (nomal and CF) | CSF | MetaCore | article | 1 |
| HETE and HPETE biosynthesis and metabolism | CSF | MetaCore | article | 1 |
| Neurophysiological process_Role of CDK5 in presynaptic signaling | CSF | MetaCore | article | 1 |
| Role of Diethylhexyl Phthalate and Tributyltin in fat cell differentiation | CSF | MetaCore | article | 1 |
| Phosphatidylinositol metabolism | CSF | MetaCore | article | 1 |
| Aldosterone biosynthesis and metabolism | CSF | MetaCore | article | 1 |
| Transcription role of VDR in regulation of genes involved in osteoporosis | CSF | MetaCore | article | 1 |
| Tyrosine metabolism p.1(dopamine) | CSF | MetaCore | article | 1 |
| **CN VS CN_AD** | | | | |

| | | | | |
|---|---|---|---|---|
| Fatty acid biosynthesis | serum | Mummichog | enrichment | 1 |
| D-Glutamine and D-glutamate metabolism | plasma | Mummichog | enrichment | 1 |
| Linoleic acid metabolism | serum | Mummichog | enrichment | 1 |
| **MCI VS MCI_AD** | | | | |
| Arginine and proline metabolism | plasma, serum | Mummichog | enrichment | 1 |
| beta-Alanine metabolism | Plasma, CSF | Mummichog | enrichment | 1 |
| Glutathione metabolism | plasma | Mummichog | enrichment | 1 |
| Glycolysis or Gluconeogenesis | serum | Mummichog | enrichment | 1 |
| Pentose phosphate pathway | serum | Mummichog | enrichment | 1 |
| pentose phosphate pathway | serum | MPEA | article | 1 |
| Pyruvate metabolism | serum | Mummichog | enrichment | 1 |
| Glycine, serine and threonine metabolism | plasma, serum | Mummichog | enrichment | 1 |
| Cysteine and methionine metabolism | plasma, serum | Mummichog | enrichment | 1 |
| D-Arginine and D-ornithine metabolism | plasma | Mummichog | enrichment | 1 |
| CHOL sphingolipid transport | plasma | MetaCore | article | 1 |
| Polyamine metabolism | plasma | MetaCore | article | 1 |
| Transport of IC CHOL | plasma | MetaCore | article | 1 |
| Glycolysis & GNG | plasma | MetaCore | article | 1 |
| Prostaglandin 2 biosynthesis and metabolism | plasma | MetaCore | article | 1 |
| Regulation of lipid metabolism | plasma | MetaCore | article | 1 |
| Ascorbate metabolism | plasma | MetaCore | article | 1 |
| Plasmalogen biosynthesis | plasma | MetaCore | article | 1 |
| Lysine metabolism | plasma | MetaCore | article | 1 |
| Tyrosine metabolism | plasma | MetaCore | article | 1 |
| Catecholamine metabolism | plasma | MetaCore | article | 1 |
| L-Arginine metabolism | plasma | MetaCore | article | 1 |
| NAE & PL A2 pathway | plasma | MetaCore | article | 1 |

Abbreviations: ORA, over-representation analysis.

[a]This column gave information on how we obtain this pathway. “Enrichment” indicated that we conducted pathway enrichment analyses using differential metabolites extracted from included studies; “article” means that original articles conducted pathway enrichment analysis and we extracted these pathways’ name directly from literature.

[b]This frequency of pathway includes the frequency reported in previous studies in this systematic review and the frequency of pathway enriched in our analysis based on the extracted metabolites.

**Supplementary Table 10. Pathways altered in Alzheimer's disease identified in brain tissue, CSF, plasma and serum**

| Pathway Name | Biosample type |
|---|---|
| Glycolysis or Gluconeogenesis | CSF |
| Aldosterone biosynthesis and metabolism | CSF |
| Aspartate and aspargine metabolism | CSF |
| Cholesterol and sphingolipids transport/transport from Golgi | CSF |
| Cortisol BS from cholesterol | CSF |
| FXR-regulated cholesterol and bile acid cellular transport | CSF |
| Glycolysis and gluconeogenesis | CSF |
| HETE and HPETE biosynthesis and metabolism | CSF |
| Neurophysiological process_Role of CDK5 in presynaptic signaling | CSF |
| Nicotine MB in liver | CSF |
| Nicotine metabolism in liver | CSF |
| Phosphatidylinositol metabolism | CSF |
| Prostaglandin 2 biosynthesis and metabolism | CSF |
| Regulation of CFTR gating (nomal and CF) | CSF |
| Role of Diethylhexyl Phthalate and Tributyltin in fat cell differentiation | CSF |
| Role of Diethylhexyl Phthalate and Tributyltin in fat differentiation | CSF |
| Saturated fatty acids metabolism | CSF |
| Transcription role of VDR in regulation of genes involved in osteoporosis | CSF |
| Tyrosine metabolism p.1(dopamine) | CSF |
| alpha-Linolenic acid metabolism | plasma |
| D-Arginine and D-ornithine metabolism | plasma |
| D-Glutamine and D-glutamate metabolism | plasma |
| Glycerophospholipid metabolism | plasma |
| Acetylcholine biosynthesis and metabolism | plasma |
| aminoacyl-tRNA biosynthesis in cytoplasm | plasma |
| Aminoacyl-tRNA biosynthesis in mitochondria | plasma |
| Aminoacyl-tRNA biosynthesis in mitochondrion | plasma |
| Arginine metabolism | plasma |
| Ascorbate metabolism | plasma |
| Bile Acid Biosynthesis | plasma |
| Catecholamine metabolism | plasma |
| CHOL & Sphingolipid transport | plasma |
| CHOL sphingolipid transport | plasma |
| Cholesterol metabolism | plasma |
| Cortisol biosynthesis from cholesterol | plasma |
| Development Activation of astroglial cells proliferation by ACM3 | plasma |
| Development_ACM2 and ACM4 activation of ERK | plasma |
| Development_Activation of astroglia cell proliferation by ACM3 | plasma |

| | |
|---|---|
| Fatty Acid Omega Oxidation | plasma |
| FXR-regulated cholesterol and bile acid transport | plasma |
| GABA biosynthesis& metabolism | plasma |
| Glycolysis & GNG | plasma |
| Glycolysis GNG(short map) | plasma |
| Histidine-glutamate-glutamine metabolism | plasma |
| L-Arginine metabolism | plasma |
| lipid metabolism | plasma |
| Lysine metabolism | plasma |
| Mechanism of action of DGaT1 in obesity and diabetes mellitus, type II | plasma |
| melatonin signaling | plasma |
| Muscle contraction_nNOS Signaling in Skeletal Muscle | plasma |
| NAE & PL A2 pathway | plasma |
| neurophysiological process_ delta-type opioid receptor in the nervous system | plasma |
| Neurophysiological process_ ACM1 and ACM2 in neuronal… | plasma |
| Neurophysiological process_GABAergic neurotransmission | plasma |
| Niacin-HDL metabolism | plasma |
| Nicotine signaling in GABAergic neurons | plasma |
| PG 2 biosynthesis & metabolism | plasma |
| Phospholipid metabolism | plasma |
| Plasmalogen biosynthesis | plasma |
| Polyamine metabolism | plasma |
| Proline metabolism | plasma |
| prostaglandin 2 biosynthesis and metabolism | plasma |
| Regulation of lipid MB FXR-dependent negative-feedback regulation of bile acid concentration | plasma |
| Regulation of lipid metabolism | plasma |
| Role of VDR in regulation of genes involved in osteoporosis | plasma |
| Sat fatty acid biosynthesis | plasma |
| saturated fatty acid metabolism | plasma |
| Serotonin modulation of dopamine release in nicotine addiction | plasma |
| Transport ACM3 in salivary glands | plasma |
| Transport of IC CHOL | plasma |
| Triacylglycerol biosynthesis in obesity and diabetes mellitus, type II | plasma |
| Triacylglycerol metabolism | plasma |
| Tyrosine metabolism | plasma |
| UMP biosynthesis | plasma |
| Vitamin d2 metabolism | plasma |
| Vitamin D3 metabolic C-23 and C-24 pathways | plasma |
| Pentose phosphate pathway | serum |
| Fatty acid biosynthesis | serum |

| | |
|---|---|
| arginineand proline metabolism | serum |
| histidine metabolism | serum |
| pyruvatemetabolism | serum |
| Phenylalanine, tyrosine and tryptophan biosynthesis | CSF and plasma |
| Pyrimidine metabolism | CSF and plasma |
| Vitamin B6 metabolism | CSF and plasma |
| (L)-Arginine metabolism | CSF and plasma |
| Bile acid metabolism | CSF and plasma |
| Cholesterol and sphingolipids transport | CSF and plasma |
| cortisone biosynthesis and metabolism | CSF and plasma |
| Intracellular cholesterol transport | CSF and plasma |
| Regulation of CFTR gating | CSF and plasma |
| Urea cycle | CSF and plasma |
| Linoleic acid metabolism | serum and plasma |
| tryptophan metabolism | serum and plasma |
| Pyruvate metabolism | CSF and serum |
| Biotin metabolism | brain, CSF, plasma |
| Phenylalanine metabolism | CSF, plasma, serum |
| Synthesis and degradation of ketone bodies | CSF, plasma, serum |
| Alanine, aspartate and glutamate metabolism | brain, CSF, plasma, serum |
| TCA cycle | brain, CSF, plasma, serum |
| Purine metabolism | brain, CSF, plasma, serum |
| Arginine and proline metabolism | brain, CSF, plasma, serum |
| Cysteine and methionine metabolism | brain, CSF, plasma, serum |
| Glycine, serine and threonine metabolism | brain, CSF, plasma, serum |
| Pantothenate and CoA biosynthesis | brain, CSF, plasma, serum |
| Aminoacyl-tRNA biosynthesis | brain, CSF, plasma, serum |
| Nitrogen metabolism | brain, CSF, plasma, serum |
| Glutathione metabolism | brain, CSF, plasma, serum |
| beta-Alanine metabolism | brain, CSF, plasma, serum |
| Valine, leucine and isoleucine biosynthesis | brain, CSF, plasma, serum |
| Sphingolipid metabolism | brain, CSF, plasma, serum |
| Butanoate metabolism | brain, CSF, plasma, serum |
| Propanoate metabolism | brain, CSF, plasma, serum |

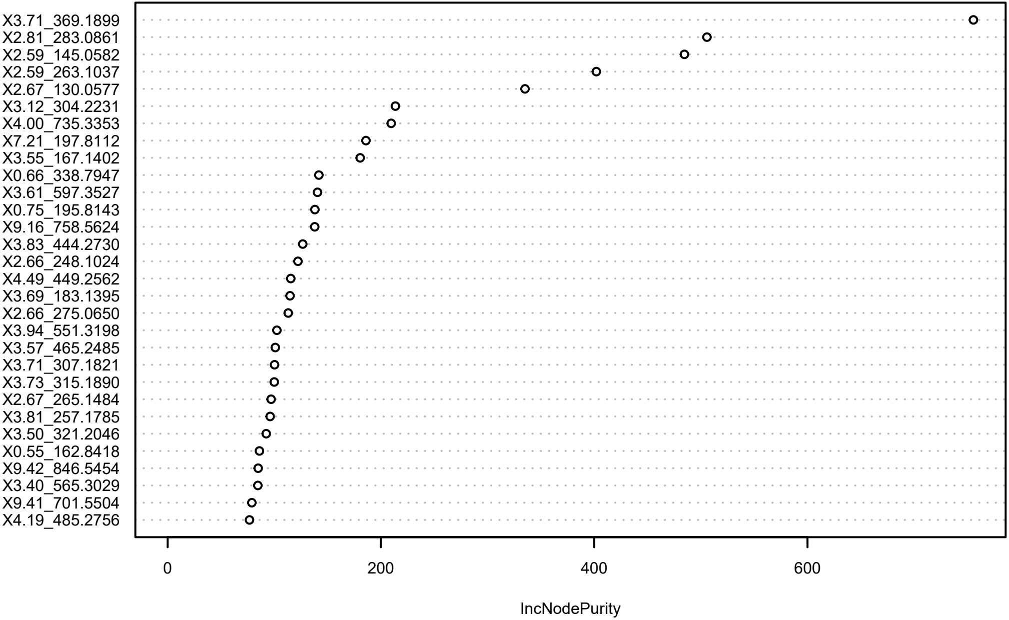


Supplementary Fig. 1

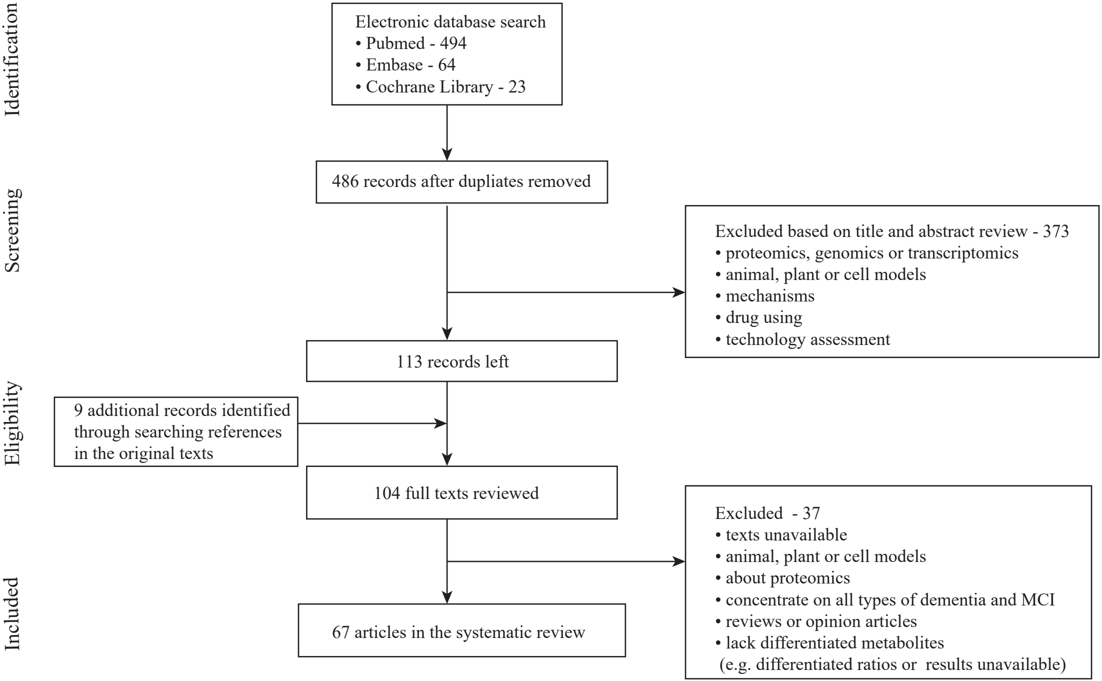


Supplementary Fig. 2

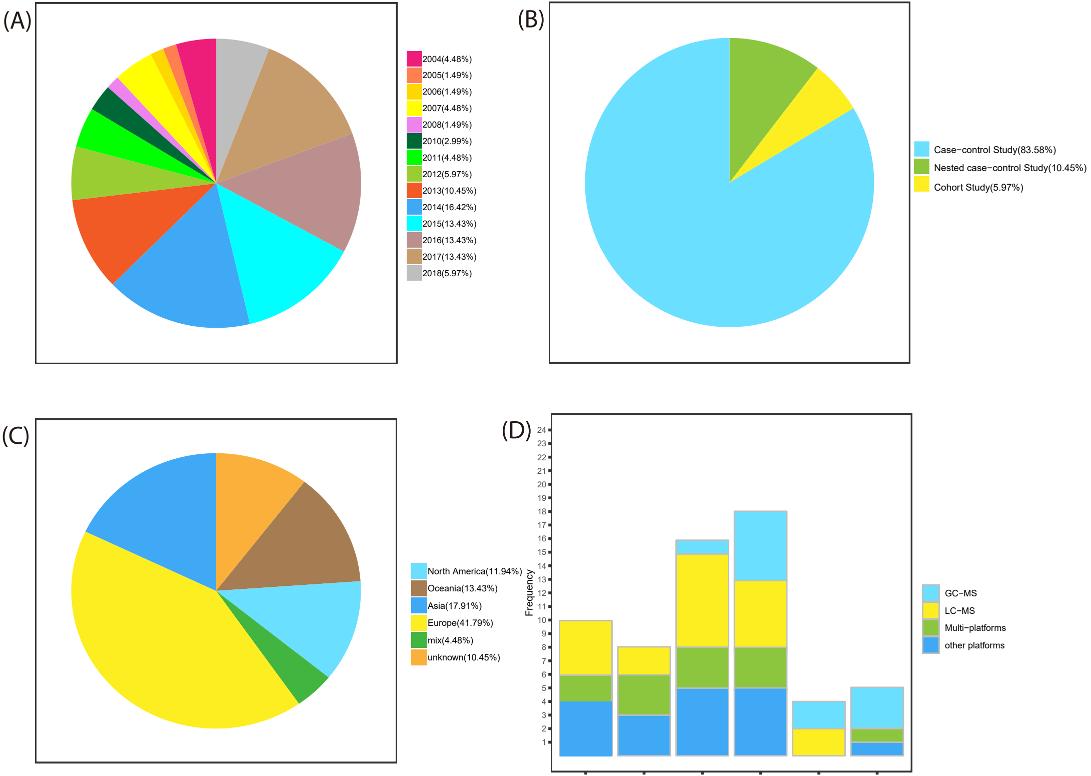


Supplementary Fig. 3
