# Supplementary material for: High-Throughput Metabolomics for Discovering Potential Biomarkers and Identifying Metabolic Mechanisms in Aging and Alzheimer’s Disease
Source: Front Cell Dev Biol. 2021 Feb 25;9:602887. doi: 10.3389/fcell.2021.602887 (PMC7947003; doi:10.3389/fcell.2021.602887)
Supplement: Supplementary file 2 [file Table_1.DOCX]

**Table 1. Top 30 aging-related metabolites in plasma screened by random forest**

| **Categories/Number** | **RT^a^** | **Mass** | **HMDB ID^b^** | **Metabolites’ Name** | **Additive ion** | **ESI Mode** | |
| --- | --- | --- | --- | --- | --- | --- | --- |
| Dipeptides | | | | | | | |
| 1 | 2.81 | 283.0861 | HMDB0028853 | Glycyl-Tyrosine | M + FA-H | ESI- | |
| 2 | 2.66 | 248.1024 | HMDB0029060 | Threoninyl-Glutamate | M+H | ESI+ | |
| 3 | 2.67 | 265.1484 | HMDB0029008 | Phenylalanyl-Valine | M+H | ESI+ | |
| long-chain fatty acids | | | | | | | |
| 4 | 3.71 | 307.1821 | HMDB0000672 | Hexadecanedioic acid | M+Na-2H | ESI- | |
| 5 | 3.81 | 257.1785 | HMDB0000872 | Tetradecanedioic acid | M-H | ESI- | |
| **6** | **3.50** | **321.2046** | **HMDB0000827** | **Stearic acid** | **M+K-2H** | **ESI-** | |
| Triterpenoids | | | | | | | |
| 7 | 4.49 | 449.2562 | HMDB0002385 | Celastrol | M-H | ESI- | |
| 8 | 3.94 | 551.3198 | HMDB0004309 | Triterpenoid | M-H | ESI- | |
| Steroid glucuronide conjugates | | | | | | | |
| 9 | 3.61 | 597.3527 | HMDB0002513 | Lithocholate 3-O-glucuronide | M + FA-H | ESI- | |
| 10 | 3.4 | 565.3029 | HMDB0002577 | Cholic acid glucuronide | M-H20-H | ESI- | |
| Fatty acid esters | | | | | | | |
| 11 | 3.69 | 183.1395 | HMDB0031272 | Ethyl (E)-2-nonenoate | M-H | ESI- | |
| 12 | 3.57 | 465.2487 | HMDB0029886 | Sorbitan oleate | M+K-2H | ESI- | |
| Phosphatidylcholine | | | | | | | |
| 13 | 9.16 | 758.5624 | HMDB0007880 | PC(14:0/20:2(11Z,14Z)) | M+H | ESI+ | |
| **14** | **9.42** | **846.5454** | **HMDB0007989** | **PC(16:0/22:5(4Z,7Z,10Z,13Z,16Z))** | **M+K** | **ESI+** | |
| 1,2-diacylglycerol-3-phosphates | | | | | | | |
| 15 | 9.41 | 701.5504 | HMDB0114824 | 1,2-diacylglycerol-3-phosphates | | ESI+ | |
| 1-acylglycerol-3-phosphates | | | | | | | |
| 16 | 4.19 | 485.2756 | HMDB0114752 | LysoPA(22:4(7Z,10Z,13Z,16Z)/0:0) | M-H | ESI- | |
| Gluco/mineralocorticoids, progestogins and derivatives | | | | | | | |
| **17** | **3.71** | **369.1899** | **HMDB0000315** | **16-a-Hydroxypregnenolone** | **M + K-2H** | **ESI-** | |
| Phenylpropanoic acids | | | | | | | |
| 18 | 2.59 | 145.0582 | HMDB0001955 | 3-Phenylbutyric acid | M-H20-H | ESI- | |
| Hydroxyindoles | | | | | | | |
| 19 | 2.59 | 263.1037 | HMDB0001238 | N-Acetylserotonin | M + FA-H | ESI- | |
| Hypoxanthines | | | | | | | |
| 20 | 2.67 | 130.0577 | HMDB0000897 | 7-Methylguanine | M-2H2O+H | ESI+ | |
| Acylcarnitines | | | | | | |  |
| 21 | 3.12 | 304.2231 | HMDB0061634 | 3-hydroxyoctanoyl carnitine | M+H | ESI+ | |
| Phosphatidylglycerophosphates | | | | | | | |
| 22 | 4.00 | 735.3353 | HMDB0033168 | (15a,20R)-Dihydroxypregn-4-en-3-one 20-[glucosyl-(1->4)-6-acetyl-glucoside] | M+K-2H | ESI- | |
| Medium-chain fatty acids | | | | | | | |
| 23 | 3.55 | 167.1402 | HMDB0000947 | Undecanoic acid | M-H20-H | ESI- | |
| Oligopeptides | | | | | | | |
| 24 | 3.83 | 444.273 | HMDB0012936 | Dynorphin B (10-13) | M-H | ESI- | |
| Purine 2'-deoxyribonucleosides | | | | | | | |
| 25 | 2.66 | 275.065 | HMDB0000071 | Deoxyinosine | M+Na | ESI+ | |
| Prostaglandins and related compounds | | | | | | | |
| 26 | 3.73 | 315.189 | HMDB0060046 | 15d PGD2 | M-H20-H | ESI- | |

^a^Rention time. ^b^the HMDB identifier of the metabolite.

Bold, 3 metabolites related to both aging and AD, including 16-a-Hydroxypregnenolone, stearic acid, and PC(16:0/22:5(4Z,7Z,10Z,13Z,16Z)).
