# Supplementary material for: High-Throughput Metabolomics for Discovering Potential Biomarkers and Identifying Metabolic Mechanisms in Aging and Alzheimer’s Disease
Source: Front Cell Dev Biol. 2021 Feb 25;9:602887. doi: 10.3389/fcell.2021.602887 (PMC7947003; doi:10.3389/fcell.2021.602887)
Supplement: Supplementary file 3 [file Table_2.DOCX]

**Table 2. Aging-related pathways enriched by spearman-correlated metabolites**

| **Pathways’ Name** | **Total No.** | **Hits No.** | ***P*-value** |
| --- | --- | --- | --- |
| Carnitine shuttle^a^ | 15 | 4 | 0.021 |
| Omega-3 fatty acid metabolism^a^ | 5 | 2 | 0.035 |
| Ascorbate (Vitamin C) and Aldarate Metabolism^b^ | 2 | 2 | 0.007 |
| Glutamate metabolism^b^ | 6 | 3 | 0.010 |
| Biopterin metabolism^b^ | 10 | 4 | 0.012 |
| TCA cycle^b^ | 3 | 2 | 0.019 |
| Vitamin B3 (nicotinate and nicotinamide) metabolism^b^ | 15 | 5 | 0.021 |
| Purine metabolism^b^ | 23 | 7 | 0.023 |
| Tyrosine metabolism^b^ | 31 | 9 | 0.026 |
| Aspartate and asparagine metabolism^b^ | 31 | 9 | 0.026 |
| Carnitine shuttle^b^ | 12 | 4 | 0.031 |
| Leukotriene metabolism^b^ | 21 | 6 | 0.044 |
| Arginine and Proline Metabolism^b^ | 17 | 5 | 0.045 |

^a^Pathway enrichment conducted using negative-mode features.

^b^Pathway enrichment conducted using positive-mode features.
