# Supplementary material for: High-Throughput Metabolomics for Discovering Potential Biomarkers and Identifying Metabolic Mechanisms in Aging and Alzheimer’s Disease
Source: Front Cell Dev Biol. 2021 Feb 25;9:602887. doi: 10.3389/fcell.2021.602887 (PMC7947003; doi:10.3389/fcell.2021.602887)
Supplement: Supplementary file 4 [file Table_3.DOCX]

**Table 3. Metabolic biomarkers of Alzheimer’s disease and** **mild cognitive impairment replicated in both case-control and nested case-control studies**

| **Metabolites’**  **Name** | **HMDB ID** | **Biosample’s type** | | |
| --- | --- | --- | --- | --- |
|  |  | **AD VS. CN**  **(reported frequency**^a^**)** | **CN_AD VS. CN (reported frequency)** | |
| Palmitic acid | HMDB0000220 | Brain tissue, serum (6) | Serum (1) | |
| Stearic acid | HMDB0000827 | Brain tissue, serum (2) | Serum (1) | |
| Linoleic acid | HMDB0000673 | Brain tissue, serum (2) | Serum (1) | |
| Glutamine | HMDB0000641 | Brain tissue, serum, urine (3) | | Plasma (1) |
| Oleic acid | HMDB0000207 | Brain tissue, serum (4) | Serum (1) | |
| Myristic acid | HMDB0000806 | Serum (2) | Serum (1) | |
|  |  | **AD VS. MCI**  **(reported frequency)** | **MCI_AD VS. MCI**  **(reported frequency)** | |
| Arginine | HMDB0000517 | Plasma, CSF (1) | CSF (1) | |
| Creatine | HMDB0000064 | Serum (2) | Plasma, CSF (2) | |
| Histidine | HMDB0000177 | Serum (2) | CSF (1) | |

AD VS. CN, AD VS. MCI, the comparison in case-control studies;

CN_AD VS. CN, MCI_AD VS. MCI, the comparison in nested case-control studies;

AD, Alzheimer’s disease; CN, healthy controls; MCI, mild cognitive impairment.

^a^Reported frequency of the metabolite in previous studies included in this systematic review.
